# Species-specific responses of marine bacteria to environmental perturbation

**DOI:** 10.1038/s43705-023-00310-z

**Published:** 2023-09-22

**Authors:** Tito D. Peña-Montenegro, Sara Kleindienst, Andrew E. Allen, A. Murat Eren, John P. McCrow, Juan D. Sánchez-Calderón, Jonathan Arnold, Samantha B. Joye

**Affiliations:** 1 Department of Marine Sciences, University of Georgia, 325 Sanford Dr., Athens, GA 30602-3636 USA; 2 Institute of Bioinformatics, University of Georgia, 120 Green St., Athens, GA 30602-7229 USA; 3 Grupo de Investigación y Desarrollo en Ciencias, Tecnología e Innovación (BioGRID), Sociedad de Doctores e Investigadores de Colombia (SoPhIC), Bogotá, Colombia; 4 Microbial and Environmental Genomics, J. Craig Venter Institute, La Jolla, CA 92037 USA; 5 Integrative Oceanography Division, Scripps Institution of Oceanography, UC San Diego, La Jolla, CA 92037 USA; 6 Helmholtz Institute for Functional Marine Biodiversity at the University of Oldenburg, University of Oldenburg, Oldenburg, 26129 Germany; 7 Josephine Bay Paul Center, Marine Biological Laboratory, Woods Hole, MA USA; 8 Grupo de Investigación en Gestión Ecológica y Agroindustrial (GEA), Programa de Microbiología, Facultad de Ciencias Exactas y Naturales, Universidad Libre, Seccional Barranquilla, Barranquilla, Colombia; 9 Department of Genetics, University of Georgia, 120 Green St., Athens, GA 30602-7223 USA; 10 Present Address: Department of Environmental Microbiology, Institute for Sanitary Engineering, Water Quality and Solid Waste Management (ISWA), University of Stuttgart, Bandtäle 2, 70569 Stuttgart, Germany

**Keywords:** Environmental microbiology, Microbial ecology

## Abstract

Environmental perturbations shape the structure and function of microbial communities. Oil spills are a major perturbation and resolving spills often requires active measures like dispersant application that can exacerbate the initial disturbance. Species-specific responses of microorganisms to oil and dispersant exposure during such perturbations remain largely unknown. We merged metatranscriptomic libraries with pangenomes to generate **C**ore-**A**ccessory Metatranscriptomes (CA-Metatranscriptomes) for two microbial hydrocarbon degraders that played important roles in the aftermath of the Deepwater Horizon oil spill. The *Colwellia* CA-Metatranscriptome illustrated pronounced dispersant-driven acceleration of core (~41%) and accessory gene (~59%) transcription, suggesting an opportunistic strategy. *Marinobacter* responded to oil exposure by expressing mainly accessory genes (~93%), suggesting an effective hydrocarbon-degrading lifestyle. The CA-Metatranscriptome approach offers a robust way to identify the underlying mechanisms of key microbial functions and highlights differences of specialist-vs-opportunistic responses to environmental disturbance.

## Introduction

The ocean is the Earth’s oldest and most dynamic habitat. Microbes form the base of the ocean’s food web and moderate key ecosystem services [1, 2], producing half of the Earth’s oxygen each year, sequestering carbon via the primary fixation of inorganic carbon into biomass, modulating global biogeochemical cycles, and transforming and detoxifying pollutants [3]. Marine microbial populations are taxonomically and functionally diverse [4]. The low abundant “rare biosphere” accounts for a majority of the phylogenetic diversity in microbial populations [5, 6] and represents a tremendous repository of metabolic functionality [7]. Across diverse systems such as the human microbiome [8], soils [9, 10], the gut microbiome of soil fauna [11], aquatic systems [12, 13], and marine sediments [14], disturbances influence microbial community structure and function in ways that impact the ecosystem services that microorganisms provide [15]. Identifying why certain taxa respond to disturbances more effectively than others represents a key step towards achieving predictability of the functional capacity of microbial populations and the ecosystem dynamics they mediate. An improved understanding of the mechanisms by which key members of microbial communities respond to disturbance could provide a unifying framework, bridging biogeochemistry, microbial ecology, and related scientific disciplines [16].

Oil spills are a frequent perturbation to marine – and other – environments, yet much remains to be learned about how these perturbations alter oceanic microbial communities [17]. We explored data generated during a laboratory simulation of an oil spill [18] to identify how key members of the microbial rare biosphere responded to different environmental conditions. In April 2010, a catastrophic explosion on the Deepwater Horizon drilling rig sank the platform, initiating an uncontrolled discharge of 4.9 million barrels of crude oil into the Gulf of Mexico over 84 days. A key active measure to the oil spill was the application of seven million liters of synthetic dispersants (Corexit EC9500A and EC9527A) to surface oil slicks, and to the discharging wellhead at 1500 m water depth [19, 20]. Dispersant application enhanced oil droplet formation and aqueous phase solubilization of oil with a secondary aim of stimulating biodegradation [18]. However, dispersants had negative effects on microbial communities [18, 21, 22]. A more comprehensive understanding of how dispersants impact microorganisms involved in hydrocarbon oxidation may reveal how key microorganisms respond to disturbance, providing key information necessary to predict the trajectory and rate of recovery of baseline microbial communities in the aftermath of major disturbances. The approach presented here – merging metatranscriptomics with pangenomic data – is a robust first step to identify how individual species respond to perturbation and promotes a mechanistic understanding of impact(s) of environmental disturbance. We assessed the response of key oil-degrading taxa to dispersant and/or oil exposure in microcosms and found that patterns of ecological succession in the microcosms mirrored those observed in the deepwater oil plumes during the Deepwater Horizon oil well blowout [23–25]. Indigenous hydrocarbon degraders occupy the rare biosphere of Gulf waters [7], and are primed to respond to hydrocarbon inputs from natural or anthropogenic sources [17]. Seven weeks after the Deepwater Horizon incident began, the community dominated by *Oceanospirillales* shifted to a community characterized by abundant *Cycloclasticus* and *Colwellia* [26–28]. *Colwellia* is a genus of psychrophilic marine bacteria [29], first isolated from the Puerto Rico Trench. *Colwellia* has not been shown to degrade oil or oil components, but a single amplified genome possessed the genes necessary to degrade alkanes and aromatic hydrocarbons [30]; *Colwellia* can also produce unique and relevant glycoproteins and natural surfactants [31, 32]. *Colwellia* spp. responded rapidly in situ during the Deepwater Horizon incident [28, 33], and in experiments where Gulf seawater was amended with oil, dispersed oil, or dispersant [18, 28, 33, 34]. *Marinobacter*, first described in 1992 [35], colonizes a broad range of marine habitats [36–41] and utilizes a remarkable range of electron acceptors and electron donors [42]. *Marinobacter* strains degraded alkanes [43] and polycyclic aromatic hydrocarbons (PAH) under anoxic conditions during the Deepwater Horizon incident [44]. Chemical dispersants inhibit *Marinobacter* sp. [18, 21, 22, 45]. Recently, Rughöft et al. showed reduced growth and hydrocarbon biodegradation in starved cultures of *Marinobacter* sp. TT1 following Corexit EC9500A exposure, suggesting that substrate availability and nutritional status poises its response to environmental perturbation [37]. The ecological fitness of microorganisms such as *Marinobacter* and *Colwellia* may influence their response to environmental perturbation, especially to oil and dispersant exposure.

We examined the transcriptional signatures from lab microcosm experiments [18] to investigate the physiological drivers of niche partitioning in *Colwellia* and *Marinobacter* through assessing Differentially Expressed (DE) genes in the context of their pangenomes. We refer to the mapping of metatranscriptomic signals in the context of core versus accessory genome as the “CA-Metatranscriptome”. Application of this approach revealed distinct modes of response to environmental perturbation and offers a robust and powerful way to track and understand species-specific responses to acute and chronic perturbations in other systems.

## Materials and methods

### Initial experiments

The procedures and protocols for the experiments were presented previously [18, 46, 47]. Briefly, Kleindienst et al. [18] simulated environmental conditions in the oil-rich 1100 m deep water plume that formed during the Deepwater Horizon spill. The experiment tracked the impacts of oil-only (supplied as a water-accommodated fraction, hereafter “WAF”), synthetic dispersant (Corexit 9500; “DISP”), oil-Corexit mixtures (chemically enhanced water-accommodated fraction, “CEWAF”) and chemically enhanced water-accommodated oil fraction with nutrients (hereafter, “CEWAFN”) additions to naturally-occurring microbial populations in deep-water collected from a natural seep site in the Gulf of Mexico (Green Canyon lease block 600, e.g., GC600). The experiment was designed to reveal the impact of distinct exposure regimes resulting from infusions of organic carbon derived from oil, synthetic dispersant, or oil and synthetic dispersant on microbial community evolution and activity over time. Exposure to different organic carbon regimes is known to drive diverging patterns of microbial community composition and activity [48]. Kleindienst et al. [18] observed that exposure to synthetic dispersant alone did not enhance heterotrophic microbial activity or hydrocarbon oxidation rates [18] but it did increase the abundance of *Colwellia*, key players during the Deepwater Horizon oil spill as evidenced by their enrichment in the deep-sea plume [7]. In contrast, exposure to oil, but not synthetic dispersant, increased the abundance of *Marinobacter*. In addition, hydrocarbon oxidation rates were elevated in the presence of WAF. Here, we expand on the findings of Kleindienst et al. [18] by analyzing and interpreting metatranscriptomic data from their experiment and by applying the CA-Metatranscriptome approach to shed light on the mechanisms employed by the key microbial players *Colwellia* and *Marinobacter* to respond to oil and dispersant exposure. Further analyses from the broader metatranscriptomic assessment is presented by Peña-Montenegro et. al (submitted) [47].

### Data workflow and background pangenomes

This work involved the following steps: A) the curation of a pangenome for each of the target organisms (i.e., *Colwellia* and *Marinobacter*); B) the processing of transcriptomic signals and statistical tests to determine DE genes; and C) the generation of CA-Metatranscriptomes.

We followed a previously reported protocol for the pangenomic analysis [49]. All available complete and near-to-complete genomes in NCBI of *Colwellia* (*n* = 77) and *Marinobacter* (*n* = 171) (Supplementary Tables 1 and 2) were processed in Anvi’o (v.7.1) [50]. Anvi’o genome databases were generated with the *anvi-gen-genomes-storage* program to store all genomes and associated annotations. Each pangenome was computed with the *anvi-pan-genome* program to identify gene clusters. Background pangenomes were later used to generate CA-Metatranscriptomes (see below).

### RNA sequencing

RNAseq library preparation, sequencing, and further data processing details are described in Peña-Montenegro et. al (submitted) [47] and all scripts are found on Github (https://github.com/biotemon/K2015). Anvi’o can accept only one reference genome and one or more (meta)transcriptomes to calculate appropriate read recruitment layers for each gene cluster. To fulfill the “one reference genome” requirement, we identified the best genome reference using an all-against-all recruitment screening approach, as published previously [51–53]. All metatranscriptomic libraries were aligned to all available complete and near-to-complete genomes in NCBI of *Colwellia* (*n* = 77) and *Marinobacter* (*n* = 171) (Supplementary Tables 1 and 2). The largest mapping recruitment (i.e., average mapping counts and average mapping rates) was used to identify the best reference genome from NCBI for each species. For *Colwellia*, ~13.4 million reads were recruited for the Metagenome Assembled Genome (MAG) assigned to Candidatus *Colwellia aromaticivorans*, which we refer as *Colwellia* MAG-2 (Accession Number: QOLD00000000.1) henceforth. For *Marinobacter*, ~1.7 million reads were recruited by *Marinobacter* sp. C18 (Accession Number: LQXJ00000000.1) (Supplementary Data 1). SAM files were sorted and indexed into BAM files for later processing in the DE analysis and CA-Metatranscriptome generation.

Differential expression analysis was processed in R (v.4.3.0) as follows. Transcript counts were extracted by the mapping files using *ht-seq* and subsequently normalized using the Transcripts per Million (TPM) approach, defined as the relative abundance of an expressed gene maintaining comparability across samples and treatments [54]. TPM profiles were processed using *DESeq2* (v.1.26.0) using the regularized logarithm transformation [55]. Contrasts with respect to the biotic control (i.e., treatments without amendments) were performed using the negative binomial Wald test with Cook’s distance to control for outliers. Genes with an adjusted *p*-value < 0.05 determined using the Benjamini–Hochberg method were classified as DE genes. Mapping counts and normalized scores for all DE genes are shown in Supplementary Data 2.

### Generating CA-metatranscriptomes

To explore ecological implications of DE genes in the context of the *Colwellia* and *Marinobacter* pangenomes, we followed the workflow outlined in reference [49] and http://merenlab.org/2016/11/08/pangenomics-v2/, with minor modifications. Additional layers including the detection of DE genes in gene clusters were included to the pangenomic databases using the *program anvi-import-misc-data*. We visualized the CA-Metatranscriptome using the program *anvi-display-pan*. Using the *anvi-display-pan* interactive interface we searched for all gene clusters detected in all the genomes to assign them into the bin CORE. All scripts are found on Github (https://github.com/biotemon/K2015). Anvi’o procedures for *Colwellia* and *Marinobacter* CA-Metatranscriptomes are described in Supplementary Data 3 and 4, respectively.

Pathway visualization for Fig. 3 was generated using the iPath 3 module based on KEGG orthology numbers. An interactive version of the metabolic map is available at https://pathways.embl.de/ipath3.cgi?s=xqvYg5kHHCsxGqWy8yJ.

## Results

**Fig. 1:**
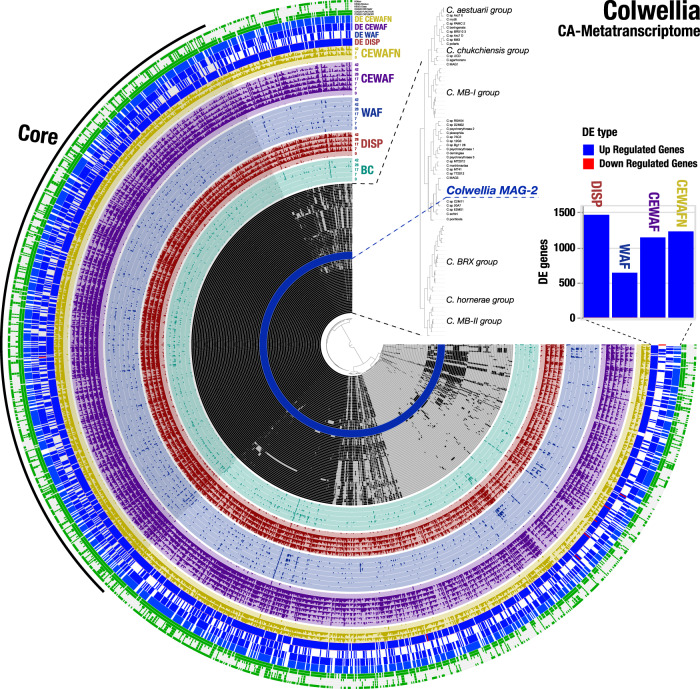
Gene detection of metatranscriptomic reads in the context of the core and accessory genome in *Colwellia*. The 77 inner layers show the presence-absence of 1598 gene clusters with 89,678 genes that were identified in 75 *Colwellia* genomes and three *Colwellia* metagenomic assembled genomes (MAGs). An expanded dendrogram of the reference genomes based on the distribution of gene clusters using Euclidian distance and Ward’s clustering is shown in the top-right. Some genome clusters were highlighted in gray blocks, and they are described in Supplementary Table 1. *Colwellia* MAG-2 (highlighted in blue) was the reference genome that recruited the largest fraction of transcriptomic reads among *Colwellia* genomes. Gene detection profiles of metatranscriptomic reads recruited by *Colwellia* MAG-2 are sorted, and color coded by the corresponding experimental treatments: Biotic control (BC), Dispersants (DISP), Water Accommodated oil Fraction (WAF), Chemically Enhanced Water Accommodated oil Fraction (CEWAF) and Chemically Enhanced Water Accommodated oil Fraction plus Nutrients (CEWAFN). Time is expressed in days. The next four layers show the presence-absence of DE genes with respect to the biotic control treatment that are shown in blue (up-regulation) and red (downregulation). A stacked-bar diagram on the right describes the DE gene counts across treatments following the color code for each of the treatments. The next six layers describe the gene clusters annotations in terms of KOfam, KEGG module, KEGG class, COG20 pathway, COG20 function and COG20 category. A detailed description of these DE genes is shown in Supplementary Data 2.

**Fig. 2:**
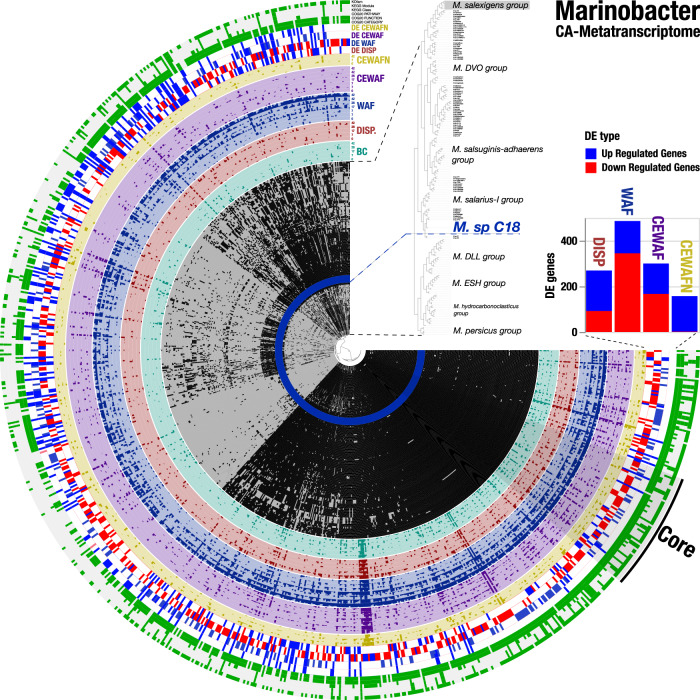
Gene detection of metatranscriptomic reads in the context of the core and accessory genome in *Marinobacter*. The 171 inner layers show the presence-absence of 826 gene clusters with 98,405 genes that were identified in 180 *Marinobacter* genomes. An expanded dendrogram of the reference genomes based on the distribution of gene clusters using Euclidian distance and Ward’s clustering is shown in the top-right. Some genome clusters were highlighted in gray blocks, and they are described in Supplementary Table 2. We defined the following groups based on their relevant representative species: **DVO** including *M*. ***d****aqiaonensis, M*. ***v****ulgaris*, and *M*. ***o****rientalis;* **DLL** including *M*. ***d****aepoensis, M*. ***l****itoralis, and M*. ***l****utaoensis;* and **ESH** including *M*. ***e****xcellens, M*. ***s****hengliensis*, and *M*. ***h****alophilus*. *Marinobacter* sp. C18 (highlighted in blue) was the reference genome that recruited the largest fraction of transcriptomic reads among *Marinobacter* genomes. Gene detection profiles of metatranscriptomic reads recruited by *Marinobacter* sp. C18 are sorted, and color coded by the corresponding experimental treatment: Biotic control (BC), Dispersants (DISP), Water-accommodated oil fraction (WAF), chemically enhanced water accommodated oil fraction (CEWAF) and chemically enhanced water accommodated oil fraction plus nutrients (CEWAFN). Time is expressed in days. The next four layers show the presence-absence of DE genes with respect to the biotic control treatment are shown in blue (up-regulation) and red (down-regulation). A stacked-bar diagram on the right describes the DE genes counts across treatments following the color code for each of the treatments. The next six layers describe the gene clusters annotations in terms of KOfam, KEGG module, KEGG class, COG20 pathway, COG20 function and COG20 category. A detailed description of these DE genes is shown in Supplementary Data 2.

Kleindienst et al. [18] demonstrated differences in the response of *Colwellia* and *Marinobacter* abundance to specific cocktails of organic carbon [46] (Materials and Methods). Four triplicated microcosms (DISP, WAF, CEWAF, CEWAFN) and triplicated biotic controls were sampled over a span of six weeks (at 0, 7, 17, 28, and 42 days, except CEWAFN treatments, which were only sampled at 0, 7, and 42 days) and transcriptomic libraries were generated for each sampling point. For sampling points WAF_t=7d_, WAF_t=42d_, CEWAF_t=7d_, and CEWAF_t=42d_ we generated duplicate transcriptomic libraries. The present study revealed the mechanistic response of the microbial community through assessment of DE genes mapped onto pangenomes. We identified the best reference genome for *Colwellia* and *Marinobacter*, as described above, and mapped metatranscriptomic libraries to all available complete and near to complete genomes from NCBI for these microorganisms (Supplementary Tables 1 and 2, Supplementary Data 1). The oil-only treatment showed the largest number of DE genes for *Marinobacter*, and the smallest number of DE genes for *Colwellia* (red and blue histograms in Figs. 1 and 2); all DE contrasts were calculated relative to the biotic control. In contrast, for *Colwellia*, the largest number of DE genes was observed in dispersant-amended treatments, while the smallest number of DE genes for *Marinobacter* occurred in dispersant-amended treatments. The distribution of upregulated genes ranged from 151 to 257 for *Marinobacter* and from 644 to 1472 for *Colwellia*.

In contrast, the distribution of downregulated genes ranged from 18 to 277 for *Marinobacter* and from 0 to 7 for *Colwellia*. Though both *Colwellia* and *Marinobacter* were present at low abundance initially (*Colwellia* at day 0: 16S rRNA gene abundance = 0.56%, metatranscriptomic abundance = 6.11%; *Marinobacter* at day 0: 16S rRNA gene abundance = 1.37%, metatranscriptomic abundance = 8.00%), and *Colwellia* was less abundant than *Marinobacter*; *Colwellia* exhibited a much stronger up regulation response to dispersants and/or hydrocarbons, while *Marinobacter* exhibited a comparable response regarding up and down regulation in response to dispersants and/or hydrocarbons.

To identify species-level functional traits that exhibited a transcriptional response to the imposed treatment regimes, we co-investigated the transcript abundance, activity, and the gene pool of *Colwellia* and *Marinobacter* populations across the microcosms. To this end, we extended the workflow implemented in *Anvi’o* for metapangenomics [49, 50] with metatranscriptomics, which we refer to as ‘CA-Metatranscriptome’, and we grouped gene clusters that contained at least one DE gene for downstream analyses (Figs. 1 and 2). Additionally, gene clusters were grouped into core and accessory gene clusters, based on their occurrence in all genomes or only a sub-set of genomes, respectively. In general, the core pangenome generates the broad ecological and phenotypic properties of a species [50–53]. In contrast, the accessory (or auxiliary) pangenome is associated with strain-specific, isolate-specific, rare- or common-to-subset fractions of a pangenome [56–59]. Whether the contribution of the accessory pangenome at the expression level is advantageous to phenotypes under selective conditions, especially in a harsh environment posed by oil and dispersants, is still an open question [60]. However, the accessory genome is known to promote adaptability in pathogens and may serve a similar function in other organisms [61]. By merging pangenomics, and metatranscriptomics in the same framework, CA-Metatranscriptomes enabled exploration of microbial in situ niche-contribution to relative transcriptional activity to identify the metabolic determinants of a particular microorganism’s response to environmental perturbation.

The *Colwellia* CA-Metatranscriptome (Fig. 1) included 89,678 gene calls across 1,598 gene clusters. 40.9 % (*n* = 653) of the gene clusters were grouped into the core pangenome, while the rest (*n* = 945 or 59.1%) were present in the accessory pangenome. Most of the gene clusters contained DE genes with functional annotation from KEGG/KOfam (*n* = 69%) and from COGs (*n* = 89.1%). The *Marinobacter* CA-Metatranscriptome (Fig. 2) included 98,405 gene calls across 826 gene clusters. For *Marinobacter*, 60 (7.3%) and 766 (92.7%) gene clusters were associated with the core and accessory CA-Metatranscriptome, respectively. Most of the gene clusters contained DE genes with functional annotation from KEGG/KOfam (*n* = 59.7%) and from COGs (*n* = 86.2%). So, while *Colwellia*’s transcriptomic response was proportionally split between core (~41%, defined as DE clusters in *Colwellia* core pangenome × 100 / total DE clusters in *Colwellia* pangenome) and accessory (~59%) gene clusters, *Marinobacter*’s transcriptomic response was disproportionally dominated by the response of accessory (~93%) gene clusters. Previously, hydrocarbon degrading genes were reported in the accessory genome [62]. Furthermore, core and accessory responses in *Colwellia* did not impair housekeeping functions but stimulated several niche-specific accessory functions (see details below).

*Colwellia* exhibited a robust transcriptomic response in the core CA-Metatranscriptome (Fig. 1, Supplementary Data 2, Supplementary Results). Cell membrane biogenesis and inorganic ion transport categories were upregulated in the CEWAF and CEWAFN treatments. Additional genes related to fatty acid biosynthesis were upregulated in the CEWAFN treatment. Lipid metabolism and coenzyme metabolism were also upregulated in the CEWAF treatment. About 21% of upregulated genes in the core CA-Metatranscriptome of *Colwellia* were assigned to the top three COG categories J (translation), E (amino acid transport), and N (cell motility). In contrast, 23% of upregulated genes in the accessory CA-Metatranscriptome of *Colwellia* were in the top three COG categories T (signaling mechanisms), J and E. A sub-set of functions in the core CA-Metatranscriptome of *Colwellia* were upregulated in the dispersants-only treatment, reflecting a response to Corexit components rather than oil components (Fig. 1, Supplementary Data 2, Materials and Methods). The upregulated genes were involved in oxidative phosphorylation, energy and carbohydrate metabolism, two-component sensing systems, and recombination, suggesting metabolism of dispersant components to generate cellular energy. Furthermore, a collection of genes associated to carboxylic ester hydrolysis, sulfatase and C_1_-C_2_ bond dicarboxylic activity were upregulated with the dispersed treatments suggesting the metabolic potential for DOSS (i.e., dioctyl sodium sulfosuccinate) degradation in *Colwellia*, as suggested in the bulk microcosms by Seidel et al. [46].

Addition of CEWAFN stimulated specific biosynthetic pathways in *Colwellia*’s accessory CA-Metatranscriptome (Fig. 1, Supplementary Data 2, Supplementary Results). Upregulated genes in this treatment included those involved in the synthesis of polyhydroxybutyrate, the most common polyhydroxyalkanoate, the largest known group of natural polyesters, and genes required for fatty acid degradation, such as *alkP* phosphoglycerate mutase, *ssuD* alkanesulfonate monooxygenase) [63]. Genes involved in the degradation of aromatic hydrocarbons such as phenol 2-monooxygenase and catechol 2,3-dioxygenase genes, as well as membrane transport (TonB-dependent receptor) genes, were upregulated in all dispersant-containing treatments. For instance, the *fhuA* (formerly *tonA*)*, fhuE*, and *fepA* genes were all up-regulated in the DISP and CEWAFN treatments. These genes encode for iron-binding proteins involved in iron acquisition with targeted sensitivity towards ferric coprogen, ferric-rhodotorulic acid, ferrientherochelins and colicins [30, 64–66]. Interestingly, previous studies have shown the strong relationship between siderophore production and hydrocarbon (especially PAHs) degradation. Hydrocarbon degradation involves cleaving dioxygenases that contain iron in their active sites [67]. A marine *Vibrio* spp. isolated from the Deepwater Horizon oil spill produced ochrobactins (i.e., amphiphilic siderophores) with positive enhancement of hydrocarbon degradation [68]. Furthermore, recent studies have shown clear correlation between siderophores and hydrocarbon degradation involving glutathione mediated stress responses (especially targeting reactive oxygen species), iron-driven co-metabolism, and surface area membrane activity [69–71]. Taken together, these results illustrate a sophisticated, niche specific response for *Colwellia* at the genus level.

*Marinobacter* was more transcriptionally active in the first week of the experiment (Fig. 2, Supplementary Data 2, Supplementary Results, Peña-Montenegro et al. submitted [47]), consistent with patterns of oil biodegradation when substrate was not limiting [18, 46]. The core of the *Marinobacter* CA-Metatranscriptome exhibited upregulation in response to the oil-only treatment, where 30% of these genes subscribed to repair protein genes (COG category L), chaperon protein genes (COG category O) and signal transduction associated genes (COG category T). Additionally, we observed increased transcription of a group of housekeeping genes in the COG categories H (coenzyme metabolism), and E (amino acid transport). The reduced contribution of the core CA-Metatranscriptome relative to the accessory CA-Metatranscriptome to oil-only treatments, and the even further limited response to dispersant-amended treatments suggest highly specific adaptations of *Marinobacter* sp. C18 to oil exposure and suggest that these traits are not shared with other *Marinobacter* species.

In the accessory CA-Metatranscriptome of *Marinobacter* in the WAF treatment (Fig. 2, Supplementary Data 2, Supplementary Results), upregulated genes included essential genes involved in carbon and lipid metabolism, stress associated chaperone genes, fatty acid, and aromatic carbohydrate degradation genes. The latter genes may reflect metabolism of aromatic and xenobiotic intermediates found in the oil-derived carbon pool. Genes involved in chemotaxis, sensor kinases, ion homeostasis, and type IV pilus assembly were additionally upregulated. These physiological characteristics enable *Marinobacter* to respond efficiently to oil exposure, allowing these microorganisms to sense and move towards available substrate. *Marinobacter aquaeoli*, *Pseudomonas fluoresencens*, and *Shewanella oneidensis*, which are rapid responders to environmental perturbations, showed similar genomic features as the *Marinobacter* sp. C18 accessory CA-Metatranscriptome. Interestingly, these genomic features were mapped to transposable genomic sequences, which are typically associated to the accessory pangenome [42].

**Fig. 3:**
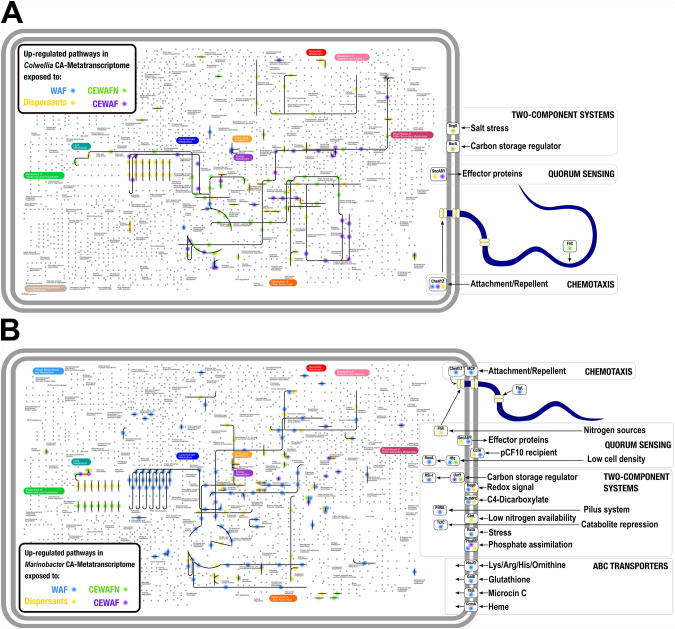
Up-regulation maps of *Colwellia* MAG-2 and *Marinobacter* sp. C18 in simulated *Deepwater Horizon* microcosms. Bold lines indicate up-regulated reactions or components in **A** *Colwellia* MAG-2 and **B** *Marinobacter* sp. C18, under the exposure of WAF (blue), CEWAF (purple), CEWAFN (green), and Dispersants (yellow). Pathway visualization was generated using the iPath 3 module based on KEGG orthology numbers. An interactive version of the metabolic map is available at https://pathways.embl.de/ipath3.cgi?s=xqvYg5kHHCsxGqWy8yJ.

To compare the architecture of metabolic reaction pathways that were upregulated across the treatments, we mapped KEGG orthology numbers into the iPath 3 module [72] (Fig. 3). Fatty acid biosynthesis routes from acetate to medium-chain fatty acyl-CoA molecules were upregulated for *Marinobacter* (in the WAF treatment) and *Colwellia* (in the dispersants treatment). Additionally, routes involving the TCA cycle (i.e., citrate – isocitrate – *cis*-aconitate conversion EC 4.2.1.3), and concomitant glycine biosynthesis of via alanine–glyoxylate transamination (EC 2.6.1.44), occurred in *Marinobacter* in the WAF treatment, and *Colwellia* in the CEWAFN treatment. Additional overlapping upregulated reactions between *Colwellia* and *Marinobacter* occurred in the oxidative phosphorylation routes and in purine metabolism. These observations matched unique functional trends over time for the *Colwellia* and *Marinobacter* communities suggesting different functional interactions in the WAF versus dispersant-amended treatments.

## Discussion

*Colwellia* signatures in the CA-Metatranscriptomes indicated opportunistic behavior in response to the chemical exposure regime and exposure time. Opportunitrophs typically show the plasticity to utilize a broad spectrum of substrates, including harmful compounds found in crude oil, without becoming exclusively dependent on one source [73–75]. In addition, the opportunistic behavior is not only defined by their energy and carbon source, but also by ability to thrive in the face of variable spatial, temporal and ecological stressors [42, 76, 77]. *Colwellia* MAG-2 DE genes varied across the dispersants-only, CEWAF and CEWAFN treatments in distinct patterns with a smaller contribution from the accessory CA-Metatranscriptome in comparison to *Marinobacter*. Therefore, *Colwellia* showed a broader niche breath, resembling a more opportunitropic lifestyle.

Although the upregulation of cytochrome P450 alkane hydroxylase genes in the *Colwellia* MAG-2 CA-Metatranscriptome was not observed in any treatment, we did observe a complex network of signaling, biogenesis, and regulation of cytochrome genes responding to oil-only (such as *cyoB*, and *ccmF*) versus dispersed treatments (such as *cyoC*, *ccmABEI*, *cccA*, and *ccsA*), suggesting WAF-dependent activation of carbohydrate oxidation. For instance, the role of the cytochrome ubiquinol oxidase (Cyo) in the modulation of hydrocarbon degradation via activation of cytochrome P450 hydroxylase genes has been documented [78] Furthermore, multiple alkane utilization pathways can coexist, even involving unknown putative monoxygenases. Currently, the coordination mechanism is unclear [79]. Thus, the identification of predicted cytochrome P450 alkane hydroxylase genes is needed to better understand the mechanism of alkane hydroxylases in *Colwellia*.

Nutrient-dependent expression shifts in *Colwellia* MAG-2 were observed in genes involved in lipid metabolism (i.e., *fadEM*, *phaC*), hydrocarbon degradation (i.e., *xylE, alkP*) and TonB-dependent receptor genes. Our observations suggest that TonB-dependent receptors could be involved in the uptake of nutrients and downstream nutrient-associated benefits to adapt to oil/dispersant exposure. Previous studies have concluded that expression of TonB dependent receptor genes is key for opportunistic adaptations to harsh environments [30, 64]. Most importantly, a large proportion of the *Colwellia* MAG-2 transcriptomic responses were associated with the core CA-Metatranscriptome, reflecting an opportunistic response at genus level (Fig. 1). These results show that *Colwellia*, at the genus level, do not dominate a particular niche. Instead, they employ genomic plasticity as a strategy to survive under conditions of Corexit exposure.

The response of *Marinobacter* illustrated the profile of a specialized and well-adapted oil degrader. In this context, a specialized oil degrader resembles the behavior of r strategists and copiotrophs. These organisms would be able to grow fast and adapt to transient, unstable and uncrowded patches of rich resources, until they die or become dormant due to substrate exhaustion [42]. Exposure to WAF led *Marinobacter* sp. C18 to up-regulate the transcription of β-oxidation genes (*fadAH, dctMP*), likely indicating increased hydrocarbon degradation activity [80]. Additionally, a wide variety of DE genes in *Marinobacter* sp. C18 were involved in interactions with its environment, such as chemotaxis genes (*cheCY, mcp, motAB*), flagellar genes (*flgLJ*, *flaA1, fliD*), and genes for type IV pilus assembly (*pilBCF, tadD*). These observations are consistent with the description of extracellular sensing and/or aggregate formation linked to hydrocarbon degradation [38, 39, 81]. The majority of WAF-specific responses were adaptations at the species level, i.e., the response was generated by the accessory CA-Metatranscriptome of *Marinobacter* sp. C18, rather than at the genus level, i.e., generated by the core CA-Metatranscriptome of *Marinobacter* sp. C18 (Fig. 2). These results match the lifestyle of a microorganism seeking an adaptive advantage in a changing environment; which usually correlates with a small core and an open pangenome [60]. *Marinobacter* is well-adapted to respond swiftly and efficiently to dissolved oil exposures in its environment.

Nutrient availability may limit a microorganism’s ability to respond to substrate enrichment or allow a microorganism to accelerate its metabolic response and/or tolerate otherwise stressful conditions. The application of oil, dispersants and nutrients – i.e., the CEWAFN treatment – shifted the upregulation map for *Colwellia* MAG-2 and *Marinobacter* sp. C18 relative to other treatments and over time (Fig. 3). In the WAF treatment, a diverse and complex response in *Marinobacter* sp. C18 was evident by DE genes involving chemotaxis, membrane two-component sensors, ABC transporters, secretion systems, quorum sensing components, fatty acid and aromatic hydrocarbon degradation routes, butyrate, propionate and glutamate assimilation, downstream biosynthesis and metabolism of nucleotides, as well as cofactors and vitamins utilized for biosynthesis. These upregulated metabolisms reflected an inherent ability to sense and metabolize hydrocarbons.

In contrast, *Colwellia* MAG-2 exhibited striking changes in the reaction map, including utilization of glutamine for the biosynthesis of inosine, a precursor for adenosine and nucleotides, consumption of L-glutamate for the biosynthesis of heme cofactors, possibly involved in the biosynthesis of P450 cytochromes and nitrogen metabolism via transcription of [Fe-S] cluster assembly proteins in the WAF treatment. Sequence alignments in PAH degrading *Mycobacterium* isolates displayed the following operon structure: a cytochrome P450 monooxygenase (CYP151 – *pipA*, CYP150 or CYP51), a ferredoxin (*fdx*), and a glutamine synthetase (*glnA*) [82]. The possibility of a dispersant-inducible operon with a similar arrangement in *Colwellia* is hypothesized based on the significant co-expression of these genes.

*Colwellia*’s response resembled a modularized network tuning reactions to specific changes in the environment (Fig. 3). Most of the altered reaction modules were not associated with membrane or signaling systems that would respond to external stimuli. Responses at the expression level involving internal metabolic modules in *Colwellia* have been documented [65, 66] and support our hypothesis that *Colwellia* acts as an individualistic opportunitroph following exposure of oil and dispersants. In contrast, the complex interaction with the surrounding environment observed in *Marinobacter* suggests community-driven, environment-dependent metabolic alterations combined with a rapid and effective ability to respond to hydrocarbon exposure at a species level.

Previous studies either provide support against [37, 40] or, interestingly, in favor of [41, 42, 75] the perception of *Marinobacter* as an opportunitroph. Our transcriptomic datasets and CA-Metatranscriptomic analysis show different expression responses across the metabolic map and suggest very different responses in a specialist-vs-opportunistic framework between *Marinobacter* and *Colwellia* (Fig. 3). In the CEWAFN treatment, *Colwellia* exhibited upregulation for the biosynthesis of glycerophospholipids, and the metabolism of amino acids, suggesting cell membrane and chaperone maintenance to combat chemical stress. Oil and dispersants are known to induce cytotoxic effects in the bacterial membrane [83], including changes in (glycerophospho)lipids [84]. For instance, the impairment of glycerophospholipids in the bacterial membrane was reported to be linked to global inhibition of dehydrogenase activity, causing reduced ATP levels and increased reactive oxidative species stress. Downstream consequences included increased amino acid utilization, possibly compensating for increased environmental susceptibility and the reduced ability to trigger membrane adaptation and maintenance [85].

Although the response of *Marinobacter* and *Colwellia* were similar in the CEWAFN treatment with regard to fatty acid biosynthesis, *Marinobacter* sp. C18 showed specific upregulation of the carbon-storage regulator CsrA, cell density sensors, and molybdenum cofactor biosynthesis. This similar response indicates that nutrients improved the fitness of *Marinobacter* in this treatment. CsrA has been described as a conserved global regulator to control central carbon pathways, motility, biofilm formation, and pathogenicity [86]. *Marinobacter*’s response to nutrients aligned with previous studies [87] where the upregulation of CsrA matched the upregulation of fatty acid biosynthesis pathway, nutrient uptake systems, maintenance of the cell envelope integrity, and regulation of iron uptake. Interestingly, *Colwellia* MAG-2 showed CsrA upregulation in treatments without nutrients, highlighting its ability to fine-tune catabolic and anabolic pathways so as to optimize energy use in response to changing conditions.

The CA-Metatranscriptome approach provided valuable insight to the complex interactions between members of the microbial community and their surrounding environment that occur in response to perturbation and confirms the hypothesis of Goyal (2018) that accessory genome components promote response and adaptability in microbial populations [88]. A complex suite of responses in *Colwellia* and *Marinobacter* CA-Metatranscriptomes revealed unique aspects of their metabolic capabilities that explained treatment-specific transcript abundances and activities. *Colwellia* exhibited an opportunistic response to organic carbon enrichment, while *Marinobacter* displayed a fine-tuned response to oil pollution. Genomic and transcriptomic plasticity promoted the success of one versus the other across the treatment regime and underscores the role of specialist-vs-opportunistic components of marine microbiomes, revealing how and why specific organisms respond to particular conditions through employment of metabolic cassettes associated with core versus accessory pangenomes.

The assessment of species-level gene content variation (i.e., the accessory pangenome) in microbial populations is needed to better understand the ecological and metabolic plasticity and how accessory genes facilitate adaptation to environmental perturbations. Previous studies have documented the core and accessory pangenome [56–59], but it is unclear whether results from the comparison of species-level pangenomes acquired from different habitats and samples reflect the ecological fitness of natural populations to respond to environmental perturbations. The present results provide further evidence that accessory genes are associated to niche-specific adaptations (i.e., oil-only responses in the *Marinobacter* accessory CA-Metatranscriptome) while core genes aligned with essential functions (i.e., a broad range response in the *Colwellia* core CA-Metatranscriptome). Interestingly, core genes can have higher recombination rates than accessory genes, as recently reported [89], which may suggest a more effective selection in *Colwellia* by environmental perturbations.

Most of the observed responses to WAF in *Marinobacter* arose from species level responses, illustrating that distinct ecotypes thrived under oil-infused conditions. In contrast, for *Colwellia*, both core and accessory CA-Metatranscriptome showed transcriptomic signals, mostly in response to dispersant addition. *Colwellia* and *Marinobacter* in dispersant-amended and WAF treatments, respectively, exhibited functional responses that followed different treatment-dependent trajectories along the metabolic map (Fig. 3). The CA-Metatranscriptome data underscores the fact that the decrease of *Marinobacter* in dispersant treatments stems from metabolic impairment – namely that dispersants inhibited *Marinobacter’s* metabolism, which decreased its abundance. In contrast, dispersants stimulated *Colwellia’s* metabolism and promoted its growth and biomass accumulation. Competitive interactions between *Marinobacter* and *Colwellia* may have further contributed to the observed selection against *Marinobacter* in dispersant treatments, but the previously reported patterns derive primarily from physiological effects that became clear in the metatranscriptomic approach of the current study.

Recent advances underscore the importance of species-specific functional responses in modulating ecological and functional signatures on the ecosystem level. Indeed, the response and subsequent recovery of microbial communities to perturbation is a key determinant of ecosystem function across a broad range of disturbances [90, 91]. Unraveling the ways through which species-specific responses to perturbation are manifest in functional change is challenging [92], though possible if focusing on a single disturbance [93]. The approach applied to the data herein reveal that unique species-specific drivers enable community-level responses to disturbance, and the CA-Metatranscriptome approach provides a blueprint for assessing microbial responses to environmental perturbation across Earth’s ecosystems, paving the way to assess how and why key microbial players respond to disturbance in a particular fashion.

### Supplementary information

Supplemental Materials

Data Set 3

Data Set 4

Data Set 2

Data Set 1

## Data Availability

All scripts are found on Github (https://github.com/biotemon/K2015). Anvi’o procedures for *Colwellia* and *Marinobacter* CA-Metatranscriptome curation are explicit in Supplementary Data 3 and 4, respectively. Raw sequencing reads generated for this study can be found in the Sequence Read Archive under the BioProject PRJNA640753. We made available Anvi’o CA-Metatranscriptome files, and Anvi’o summarized profiles in the Open Science Framework repository at 10.17605/OSF.IO/FU9BW.
