# Supplementary material for: Species-specific responses of marine bacteria to environmental perturbation
Source: ISME Commun. 2023 Sep 22;3:99. doi: 10.1038/s43705-023-00310-z (PMC10516948; doi:10.1038/s43705-023-00310-z)
Supplement: Supplementary file 1 — Supplemental Materials [file 43705_2023_310_MOESM1_ESM.pdf]

Supporting Information for

Species-specific responses of marine bacteria to environmental perturbation

Authors:

Tito D. Peña-Montenegro, Sara Kleindienst, Andrew E. Allen, A. Murat Eren, John P. McCrow, Juan D. Sánchez-Calderón, Jonathan Arnold, Samantha B. Joye*

***Corresponding author:** Samantha B. Joye

Email: mjoye@uga.edu

This PDF file includes:

Supplementary Results

Supplementary Table 1

Supplementary Table 2

Other supporting materials for this manuscript include the following:

Supplementary Data 1 to 4

Supplementary Results

For *Colwellia*, examples of genes involved in cell membrane biogenesis categories that were upregulated in CEWAF±nutrient treatments included the outer membrane protein genes *bamE* (**Supplementary Data 1**, local_id: C_CORE_719) and *ompA* (C_CORE_425), the lipoprotein signal peptidase *lpsA* gene (C_CORE_767), the lipoprotein-releasing ABC transporting *lolE* gene (C_CORE_1116, C_CORE_1118), mureine biosynthesis genes *murG* (C_CORE_133)*, murC* (C_CORE_134)*,* and *ddlA* (C_CORE_135)*,* and the flagellar protein gene *motY* (C_CORE_425). Examples of upregulation of genes related to inorganic ion transport genes include the N_2O reductase associated genes *nosD* (C_CORE_188) *nosL* (C_CORE_185) *nosZ* (C_CORE_189), TonB-dependent receptor gene *cirA* (C_CORE_114), sulfite reductase *cysI* gene (C_CORE_214), arylsulfatase A *aslA* gene (C_CORE_1151), and Na^+/H^+ antiporter genes (C_CORE_702, C_CORE_704, C_CORE_706). Fatty acid, phospholipid and isoprenoid biosynthesis genes were upregulated in the CEWAF(±nutrient) treatment, such as: *caiA* gene (C_CORE_120), *fabG* gene (C_CORE_547, C_CORE_759), *fabD* gene (C_CORE_884), *fabB* gene (C_CORE_887, C_CORE_1043) and *acc*B gene (C_CORE_739). Upregulation of coenzyme metabolism genes in the CEWAF treatment was focused on heme, folate, ubiquinone and biotin biosynthesis, as evidenced in *folA* (C_CORE_952), *folC* (C_CORE_686) and *folD* (C_CORE_525) genes.

Examples of genes that were upregulated in response to dipersants only (not oil) included the cytochrome c peroxidase *mauG* gene (C_CORE_411), nitrite reductase *nirD* gene (C_CORE_484), flagellin related protein genes *flgL* (C_CORE_638) and *flgA* (C_CORE_653), a IS1182 transposase gene (C_CORE_1110) and phage related proteins genes (C_CORE_838, C_CORE_851).

Upregulated DE genes in the CEWAF+nutrient treatment included *alkP* phosphoglycerate mutase gene (C_ACC_3190), *ssuD* alkanesulfonate monooxygenase gene (C_ACC_2939), phenol 2-monooxygenase *pheA* gene (C_ACC_2950), catechol 2,3-dioxygenase *xylE* gene (C_ACC_1810), polyhydroxyalkanoate synthase *phaC* gene (C_ACC_2206), TonB-dependent receptor genes (C_ACC_1223, C_ACC_1878, C_ACC_2013, C_ACC_2651). Additional genes

involved in fatty acid β oxidation were upregulated in the CEWAF+nutrient treatment such as acetyl-CoA C-acyltransferase *fadI* (C_ACC_28), acyl-CoA dehydrogenase *fadE* (C_ACC_1412), 3-hydroxyacyl-CoA *fadB* (C_ACC_1897), and acyl-CoA thioesterase *fadM* (C_ACC_3216).

A group of genes was upregulated in the oil-only treatment, but not in the any of the dispersed treatments, including: cytochrome c oxidase subunit I *cyoB* gene (C_ACC_370), cytochrome c oxidase subunit III *ccoP* gene (C_ACC_1348), cytochrome c oxidase subunit IV *ccoQ* gene (C_ACC_1349), the superoxide dismutase *sodA* gene (C_ACC_421), and the flagellar P-ring protein *flgL* gene (C_CORE_642).

For *Marinobacter*, upregulated genes in the accessory CA-Metatranscriptome involved in carbon and lipid metabolism included the C4-dicarboxylate transporter genes *dctM* (M_ACC_70), *dctP* (M_ACC_2186), the formate dehydrogenase gene *fdoG* (M_ACC_1859), and glycolate oxidase genes *glcD* (M_ACC_370, M_ACC_3929), *glcF* (M_ACC_372). Additional upregulated genes included some involved stress response (alkyl peroxiredoxin *bcp* gene (M_ACC_2737, M_ACC_3290, M_ACC_3406), genes involved in fatty acid degradation such as phosphoglycerate mutase *alkP* (M_ACC_397), the acetyl-CoA acyltransferase *fadA* gene (M_ACC_1907), the 2,4-dienoyl-CoA reductase *fadH* gene (M_ACC_2116), alkanesulfonate monooxygenase *ssuD* (M_ACC_2004), phenol/toluene 2-monooxygenase *dmpL* (M_ACC_2099). Upregulated genes related to chemotaxis-sensor kinases included *cheC* (M_ACC_3758), *cheY* (M_ACC_1669, M_ACC_2295, M_ACC_3796), the methyl-accepting chemotaxis *mcp* gene (M_ACC_464, M_ACC_486, M_ACC_1472, M_ACC_3455, M_ACC_3901), *motA* (M_ACC_3891, M_ACC_3138), *motB* (M_ACC_3890, M_ACC_4512), the polysaccharide biosynthesis gene *flaA1* (M_ACC_3765, M_ACC_3778), the flagellar hook-associated genes *flgJ* (M_ACC_1508), *flgL* (M_ACC_3753, M_ACC_3754), *fliD* (M_ACC_3751), the type IV pilus assembly genes *pilB* (M_ACC_3791), *pilC* (M_ACC_3792), *pilF* (M_ACC_3140. M_ACC_3400, M_ACC_3755), *tadD* (M_ACC_3803), and the OmpR family regulators *phoR* (M_ACC_41), and *phoQ* (M_ACC_347).

Upregulated genes in the core CA-Metatranscriptome of *Marinobacter* included the cyclopropane-fatty-acyl-phospholipid synthase *cfa* gene (M_CORE_152), and the N-succinylglutamate 5-semialdehyde dehydrogenase *astD* gene (M_CORE_355), involved in fatty acid metabolism. In the cell cycle control (D category) we found the stage V sporulation protein R gene *spoVR* (M_CORE_454). The 3-demethoxyubiquinol 3-hydroxylase *ubiF* (M_CORE_14) and the 2-polyprenylphenol 6-hydroxylase *ubiH* (M_CORE_206) genes, involved in ubiquinone metabolism were found in the coenzyme metabolism H category.

We inspected for differential expression of genes potentially involved in DOSS degradation. Seidel et al. (2016) model [1] proposed a two-step degradation pathway including first (reaction R1) the hydrolysis into MOSS, and later a decomposition step releasing either (reaction R2A) an $-H_2SO_3$, (reaction R2B) $-CO_2$ or (reaction R2C) $-C_8H_{18}O$ radicals. For reactions R1 and R2C, we found carboxylic ester hydrolase coding genes upregulated in the dispersant treatments for *Colwellia,* including C_ACC_758,C_CORE_874 and C_CORE_1031, and for *Marinobacter* only M_ACC_4587. For reaction R2A, the arylsulfatase A (*aslA*) gene (C_CORE_1151) was upregulared in *Colwellia.* No upregulation was observed in *Marinobacter* associated to R2A reaction genes. For reaction R2B, we inspected for upregulation in C_1-C_2 bond decarboxylase coding genes, where only accessory genes in *Colwellia* were observed, including: C_ACC_740, C_ACC_1167, C_ACC_1165, and C_ACC_1597.

Supplementary Tables

Supplementary Table 1. List of *Colwellia* reference genomes. We performed an all-vs-all screening for the best possible genome reference used for DE analysis. *Colwellia* MAG1 was the only sequence not available in NCBI since authors opted to store the MAG sequence in a different repository. *Colwellia* MAG1 was included because of its relevant description as a potential oil degrader found in a chronically polluted port [2].

Supplementary Table 1. List of *Colwellia* genomes screened for a reference.

**Local Name**	**Group in Figure 1**	**Accession Number**	**NCBI Name**	**Assembly Accession Number**	**Assembly Status**
C_MAG1		https://figshare.com/ndownloader/files/28624371	*Colwellia* POLA0515-13_bin_34	-	MAG – Contig
C_MAG2		QOLD01000001.1	Candidatus *Colwellia aromaticivorans* Bin11-node4	-	MAG – Contig
C_MAG3		JADGDH010000001.1	*Colwellia* sp. isolate BB5	-	MAG – Contig
C_aestuarii_1	C. aestuarii	NZ_MUZV01000001	*Cognaticolwellia aestuarii* (Formerly *Colwellia aestuarii*) KCTC_12480	GCF_002000025.1	Scaffold
C_aestuarii_2	C. aestuarii	NZ_NBOD01000001	*Cognaticolwellia aestuarii* (Formerly *Colwellia aestuarii*) CGMCC1.6965	GCF_002104435.1	Scaffold
C_agarivorans		NZ_MUZU01000001	*Pseudocolwellia agarivorans* (Formerly *Colwellia agarivorans*) QM50	GCF_002000085.1	Scaffold
C_beringensis		NZ_CP020465	*Cognaticolwellia beringensis* (Formerly *Colwellia* beringensis) NB097-1	GCF_002076895.1	Complete Genome
C_chukchiensis_1	C. chukchiensis	NZ_NBOC01000001	*Colwellia chukchiensis* CGMCC 1.9127-1	GCF_002104455.1	Contig
C_chukchiensis_2	C. chukchiensis	NZ_FOBI01000050	*Colwellia chukchiensis* CGMCC 1.9127-2	GCF_900109795.1	Scaffold
C_demingiae		NZ_VOLT01000010	*Colwellia demingiae* ACAM_459	GCF_007954275.1	Contig
C_echini		NZ_PJAI02000001	*Colwellia echini* A3	GCF_002843355.2	Scaffold
C_hornerae_1	C. hornerae	NZ_VOLP01000098	*Colwellia hornerae* IC037	GCF_007954305.1	Contig
C_hornerae_2	C. hornerae	NZ_VOLQ01000088	*Colwellia hornerae* IC036	GCF_007954345.1	Contig
C_hornerae_3	C. hornerae	NZ_VOLR01000097	*Colwellia hornerae* ACAM_607	GCF_007954355.1	Contig
C_marinimaniae		NZ_BDQM01000001	*Colwellia marinimaniae* MTCD1	GCF_002207865.1	Contig
C_mytili		NZ_NBOF01000001	*Cognaticolwellia mytili* (Formerly *Colwellia mytili*) KCTC_52417	GCF_002104475.1	Scaffold
C_piezophila		NZ_KB905160	*Colwellia piezophila* ATCC_BAA-637	GCF_000378625.1	Scaffold
C_polaris		NZ_NBOE01000001	*Colwellia polaris* MCCC_1C00015	GCF_002104515.1	Scaffold
C_ponticola		NZ_SZVP01000001	*Colwellia ponticola* OISW-25	GCF_005885605.1	Contig
C_psychrerythraea_1		NC_003910	*Colwellia psychrerythraea* GAB14E	GCF_000764185.1	Contig
C_psychrerythraea_2		NZ_JQEC01000001	*Colwellia psychrerythraea* ND2E	GCF_000764225.1	Contig
C_psychrerythraea_3		NZ_JQED01000001	*Colwellia psychrerythraea* 34H	GCF_000012325.1	Complete Genome

C_sp_12G3		NZ_PJAQ01000068	*Colwellia* sp. 12G3	GCF_002836775.1	Contig
C_sp_20A7		NZ_CP047130	*Colwellia* sp. 20A7	GCF_009832865.1	Complete Genome
C_sp_6M3		NZ_JACGWF010000261	*Colwellia* sp. 6M3	GCF_014077595.1	Contig
C_sp_75C3		NZ_PJAU01000033	*Colwellia* sp. 75C3	GCF_002836255.1	Contig
C_sp_Arc7_6		NZ_CP034660	*Colwellia* sp. Arc7-635	GCF_003971255.1	Complete Genome
C_sp_Arc7_D		NZ_CP028924	*Colwellia* sp. Arc7-D	GCF_003061515.1	Complete Genome
C_sp_BRX10_1	C. BRX	NZ_JACGWC010000138	*Colwellia* sp. BRX10-1	GCF_014077545.1	Contig
C_sp_BRX10_2	C. BRX	NZ_JACGWB010000170	*Colwellia* sp. BRX10-2	GCF_014077515.1	Contig
C_sp_BRX10_3		NZ_JACGWA010000032	*Colwellia* sp. BRX10-3	GCF_014077505.1	Contig
C_sp_BRX10_4	C. BRX	NZ_JACGVZ010000116	*Colwellia* sp. BRX10-4	GCF_014077465.1	Contig
C_sp_BRX10_5	C. BRX	NZ_JACGVY010000150	*Colwellia* sp. BRX10-5	GCF_014077435.1	Contig
C_sp_BRX10_6	C. BRX	NZ_JACGVX010000176	*Colwellia* sp. BRX10-6	GCF_014077455.1	Contig
C_sp_BRX10_7	C. BRX	NZ_JACGVW010000153	*Colwellia* sp. BRX10-7	GCF_014077415.1	Contig
C_sp_BRX10_9	C. BRX	NZ_JACGVV010000122	*Colwellia* sp. BRX10-9	GCF_014077405.1	Contig
C_sp_BRX8_2	C. BRX	NZ_JACGVU010000264	*Colwellia* sp. BRX8-2	GCF_014077375.1	Contig
C_sp_BRX8_3	C. BRX	NZ_JACGVT010000149	*Colwellia* sp. BRX8-3	GCF_014077355.1	Contig
C_sp_BRX8_4	C. BRX	NZ_JACGVS010000082	*Colwellia* sp. BRX8-4	GCF_014077345.1	Contig
C_sp_BRX8_5	C. BRX	NZ_JACGVR010000104	*Colwellia* sp. BRX8-5	GCF_014077325.1	Contig
C_sp_BRX8_6	C. BRX	NZ_JACGVQ010000105	*Colwellia* sp. BRX8-6	GCF_014077305.1	Contig
C_sp_BRX8_7	C. BRX	NZ_JACGVP010000140	*Colwellia* sp. BRX8-7	GCF_014077275.1	Contig
C_sp_BRX8_9	C. BRX	NZ_JACGVN010000090	*Colwellia* sp. BRX8-9	GCF_014077245.1	Contig
C_sp_BRX9_1	C. BRX	NZ_JACGVM010000100	*Colwellia* sp. BRX9-1	GCF_014077195.1	Contig
C_sp_Bg11_12	C. MB-II	NZ_JACGUZ010000001	*Colwellia* sp. Bg11-12	GCF_014076955.1	Contig
C_sp_Bg11_28		NZ_PJBA01000006	*Colwellia* sp. Bg11-28	GCF_002836245.1	Contig
C_sp_C1TZA3	C. MB-I	NZ_VOLS01000100	*Colwellia* sp. C1TZA3	GCF_007954265.1	Contig
C_sp_C2M11		NZ_JAHKQD010000039	*Colwellia* sp. C2M11	GCF_018860915.1	Scaffold

C_sp_D2M02		NZ_JAHKPW010000070	*Colwellia* sp. D2M02	GCF_018860755.1	Scaffold
C_sp_E2M01		NZ_JAHKPR010000037	*Colwellia* sp. E2M01	GCF_018860585.1	Scaffold
C_sp_MB02u_1	C. MB-I	NZ_JACGVL010000001	*Colwellia* sp. MB02u-1	GCF_014077215.1	Contig
C_sp_MB02u_10	C. MB-I	NZ_JACGVK010000001	*Colwellia* sp. MB02u-10	GCF_014077185.1	Contig
C_sp_MB02u_11	C. MB-II	NZ_JACGUY010000001	*Colwellia* sp. MB02u-11	GCF_014076925.1	Contig
C_sp_MB02u_12	C. MB-I	NZ_JACGVJ010000001	*Colwellia* sp. MB02u-12	GCF_014077155.1	Contig
C_sp_MB02u_14	C. MB-II	NZ_JACGUX010000001	*Colwellia* sp. MB02u-14	GCF_014076915.1	Contig
C_sp_MB02u_18	C. MB-I	NZ_JACGVI010000001	*Colwellia* sp. MB02u-18	GCF_014077125.1	Contig
C_sp_MB02u_19	C. MB-I	NZ_JACGVH010000001	*Colwellia* sp. MB02u-19	GCF_014077115.1	Contig
C_sp_MB02u_6	C. MB-I	NZ_JACGVG010000001	*Colwellia* sp. MB02u-6	GCF_014077095.1	Contig
C_sp_MB02u_7	C. MB-II	NZ_JACGUW010000001	*Colwellia* sp. MB02u-7	GCF_014076905.1	Contig
C_sp_MB02u_9	C. MB-I	NZ_JACGVF010000001	*Colwellia* sp. MB02u-9	GCF_014077085.1	Contig
C_sp_MB3u_22	C. MB-II	NZ_JACGUV010000001	*Colwellia* sp. MB3u-22	GCF_014076885.1	Contig
C_sp_MB3u_28	C. MB-II	NZ_JACGUU010000001	*Colwellia* sp. MB3u-28	GCF_014076795.1	Contig
C_sp_MB3u_4	C. MB-I	NZ_JACGVE010000001	*Colwellia* sp. MB3u-4	GCF_014077065.1	Contig
C_sp_MB3u_41	C. MB-II	NZ_JACGUT010000001	*Colwellia* sp. MB3u-41	GCF_014076845.1	Contig
C_sp_MB3u_43	C. MB-I	NZ_JACGVD010000001	*Colwellia* sp. MB3u-43	GCF_014076995.1	Contig
C_sp_MB3u_45	C. MB-I	NZ_JACGVC010000001	*Colwellia* sp. MB3u-45	GCF_014077005.1	Contig
C_sp_MB3u_55	C. BRX	NZ_JACGUS010000001	*Colwellia* sp. MB3u-55	GCF_014076785.1	Contig
C_sp_MB3u_64	C. MB-II	NZ_JACGUR010000001	*Colwellia* sp. MB3u-64	GCF_014076835.1	Contig
C_sp_MB3u_70	C. MB-I	NZ_JACGVB010000001	*Colwellia* sp. MB3u-70	GCF_014077045.1	Contig
C_sp_MB3u_8	C. MB-I	NZ_JACGVA010000001	*Colwellia* sp. MB3u-8	GCF_014076985.1	Contig
C_sp_MT2012		NZ_LJVG01000014	*Colwellia* sp. MT2012	GCF_001432325.1	Contig
C_sp_MT41		NZ_CP013145	*Colwellia* sp. MT41	GCF_001444365.1	Complete Genome
C_sp_PAMC_1	C. BRX	NZ_CP014944	*Colwellia* sp. PAMC 20917	GCF_001767295.1	Complete Genome
C_sp_PAMC_2		NZ_CP014943	*Colwellia* sp. PAMC 21821	GCF_002077175.1	Complete Genome

C_sp_RSH04		NZ_QXIO01000001	*Colwellia* sp. RSH04	GCF_003545765.1	Scaffold
C_sp_TT2012		NZ_LJYX01000006	*Colwellia* sp. TT2012	GCF_001440345.1	Contig
C_sp_UCD		NZ_MPHL01000001	*Colwellia* sp. UCD-KL20	GCF_001957175.1	Contig

Supplementary Table 2. List of *Marinobacter* genomes screened for a reference. We performed an all-vs-all screening for the best possible genome reference used for DE analysis.

Supplementary Table 2. List of *Marinobacter* genomes screened for a reference.

Local Name	Group in Figure 2	Accession Number	NCBI Name	Assembly Accession Number	Assembly Status
M_adhaerens_1	M. salsuginis-adhaerens	NZ_LXRF01000001	*Marinobacter adhaerens* PBVC038	GCF_001717765.1	Contig
M_adhaerens_2		NZ_JABEVQ010000010	*Marinobacter adhaerens* KG14	GCF_013393465.1	Scaffold
M_adhaerens_3	M. salsuginis-adhaerens	NZ_CP076686	*Marinobacter adhaerens* HP15-B	GCF_018972165.1	Complete Genome
M_adhaerens_4	M. salsuginis-adhaerens	NZ_JAHWRS010000001	*Marinobacter adhaerens* DP2N14-4	GCF_019335285.1	Contig
M_adhaerens_5	M. salsuginis-adhaerens	NZ_JAHXRN010000098	*Marinobacter adhaerens* CS1	GCF_019392275.1	Contig
M_adhaerens_6	M. salsuginis-adhaerens	NC_017506	*Marinobacter adhaerens* HP15	GCF_000166295.1	Complete Genome
M_alexandrii		NZ_SWKM01000011	*Marinobacter alexandrii* LZ-8	GCF_005871205.1	Scaffold
M_algicola		NZ_ABCP01000104	*Marinobacter algicola* DG893	GCF_000170835.1	Contig
M_antarcticus		NZ_FRAQ01000011	*Marinobacter antarcticus* CGMCC_1.10835	GCF_900142385.1	Contig
M_aromaticivorans		NZ_NIHD01000001	*Marinobacter aromaticivorans* D15-8P	GCF_002806975.1	Contig
M_bohaiensis		NZ_QGEH01000010	*Marinobacter bohaiensis* T17	GCF_003258515.1	Contig
M_caseinilyticus		NZ_WUQJ01000001	*Marinobacter caseinilyticus* M3-13	GCF_011074955.1	Scaffold
M_changyiensis		NZ_VZZZ01000010	*Marinobacter changyiensis* CLL7-20	GCF_009193265.1	Contig
M_confluentis_1		NZ_SRPF01000001	*Marinobacter confluentis* HJM-18	GCF_004785685.1	Scaffold
M_confluentis_2		NZ_VMHO01000010	*Marinobacter confluentis* KCTC_42705	GCF_008795935.1	Contig
M_daepoensis_1	M. DLL	NZ_ATWI01000001	*Marinobacter daepoensis* TN21-5	GCF_017255165.1	Contig
M_daepoensis_2	M. DLL	NZ_JAFKDB010000008	*Marinobacter daepoensis* DSM_16072	GCF_000421165.1	Scaffold
M_daqiaonensis_1	M. DVO	NZ_JAAFYR010000006	*Marinobacter daqiaonensis* YCSA40	GCF_012070355.1	Scaffold
M_daqiaonensis_2	M. DVO	NZ_FOYW01000007	*Marinobacter daqiaonensis* CGMCC_1.9167	GCF_900115285.1	Scaffold
M_excellens_1	M. ESH	NZ_JYNR01000001	*Marinobacter excellens* HL-55	GCF_000934705.1	Contig
M_excellens_2	M. ESH	NZ_LOCO01000001	*Marinobacter excellens* LAMA_842	GCF_001574445.1	Scaffold

M_flavimaris_1		NZ_PSSW01000001	*Marinobacter flavimaris* LMG_23834	GCF_002933295.1	Scaffold
M_flavimaris_2		NZ_QRDH01000001	*Marinobacter flavimaris* KCTC_12185	GCF_003363485.1	Contig
M_fonticola		NZ_CP043042	*Marinobacter fonticola* CS412	GCF_008122265.1	Complete Genome
M_fuscus	M. DLL	NZ_PXNP01000001	*Marinobacter fuscus* NH169-3	GCF_003007675.1	Scaffold
M_gelidimuriae		NZ_KB889827	*Marinobacter gelidimuriae* BF04_CF-4	GCF_000372805.1	Scaffold
M_gudaonensis		NZ_FOYV01000009	*Marinobacter gudaonensis* CGMCC_1.6294	GCF_900115175.1	Scaffold
M_guineae		NZ_KZ319339	*Marinobacter guineae* M3B	GCF_002744735.1	Scaffold
M_halodurans		NZ_SJDL01000001	*Marinobacter halodurans* YJ-S3-2	GCF_004327985.1	Contig
M_halophilus_1	M. ESH	NZ_PXNN01000001	*Marinobacter halophilus* JCM 30472	GCF_003007685.1	Scaffold
M_halophilus_2	M. ESH	NZ_BMFE01000001	*Marinobacter halophilus* CGMCC_1.12481	GCF_014637865.1	Scaffold
M_halotolerans		NZ_VMHP01000001	*Marinobacter halotolerans* NBRC_110910	GCF_008795985.1	Contig
M_hydrocarbonoclasticus_01	M_hydrocarbonoclasticus	NC_017067	*Marinobacter hydrocarbonoclasticus* STW2	GCF_001895225.1	Scaffold
M_hydrocarbonoclasticus_02	M. ESH	NZ_MPKY01000001	*Marinobacter hydrocarbonoclasticus* 114E_o	GCF_003315555.1	Scaffold
M_hydrocarbonoclasticus_03	M_hydrocarbonoclasticus	NZ_QNSA01000001	*Marinobacter hydrocarbonoclasticus* 105B	GCF_003337515.1	Scaffold
M_hydrocarbonoclasticus_04	M_hydrocarbonoclasticus	NZ_QPJI01000001	*Marinobacter hydrocarbonoclasticus* 114E	GCF_003337655.1	Scaffold
M_hydrocarbonoclasticus_05	M_hydrocarbonoclasticus	NZ_QPJB01000001	*Marinobacter hydrocarbonoclasticus* DSM_50418	GCF_003634635.1	Scaffold
M_hydrocarbonoclasticus_06	M_hydrocarbonoclasticus	NZ_RBJB01000002	*Marinobacter hydrocarbonoclasticus* NI9	GCF_006516615.1	Contig
M_hydrocarbonoclasticus_07	M_hydrocarbonoclasticus	NZ_VEWS01000001	*Marinobacter hydrocarbonoclasticus* NCT7M	GCF_009650625.1	Contig
M_hydrocarbonoclasticus_08	M_hydrocarbonoclasticus	NZ_WBMP01000001	*Marinobacter hydrocarbonoclasticus* MN29-10	GCF_017303195.1	Contig
M_hydrocarbonoclasticus_09	M_hydrocarbonoclasticus	NZ_JAEMWY010000001	*Marinobacter hydrocarbonoclasticus* DP3N21-9	GCF_019335185.1	Contig
M_hydrocarbonoclasticus_10	M_hydrocarbonoclasticus	NZ_JAHWRU010000001	*Marinobacter hydrocarbonoclasticus* ATCC_49840	GCF_000284615.1	Complete Genome
M_hydrocarbonoclasticus_11	M_hydrocarbonoclasticus	NC_008740	*Marinobacter hydrocarbonoclasticus* VT8	GCF_000015365.1	Complete Genome
M_lipolyticus_1		NZ_QTKT01000010	*Marinobacter lipolyticus* AESS41	GCF_018424605.1	Contig

M_lipolyticus_2		NZ_KE007306	*Marinobacter lipolyticus* SM19	GCF_000397065.2	Scaffold
M_litoralis_1	M. DLL	NZ_QMDL01000001	*Marinobacter litoralis* Sw-45	GCF_003336705.1	Contig
M_litoralis_2	M. DLL	NZ_JAELVS010000001	*Marinobacter litoralis* CARE-V18	GCF_016427555.1	Contig
M_lutaoensis_1	M. DLL	NZ_MSCW01000001	*Marinobacter lutaoensis* T5054	GCF_001981305.1	Contig
M_lutaoensis_2	M. DLL	NZ_JABWTC010000100	*Marinobacter lutaoensis* KAZ22	GCF_013371285.1	Scaffold
M_manganoxydans		NZ_AGTR01000088	*Marinobacter manganoxydans* MnI7-9	GCF_000235625.1	Contig
M_maritimus		NZ_VCGW01000010	*Marinobacter maritimus* CK47	GCF_007671675.1	Contig
M_maroccanus	M. salsuginis-adhaerens	NZ_PSSX01000001	*Marinobacter maroccanus* N4	GCF_002933275.1	Contig
M_mobilis	M. DLL	NZ_FNNE01000026	*Marinobacter mobilis* CGMCC_1.7059	GCF_900106945.1	Scaffold
M_nanhaiticus		NZ_KB822689	*Marinobacter nanhaiticus* D15-8W	GCF_000364845.1	Scaffold
M_nitratireducens		NZ_ANIE01000001	*Marinobacter nitratireducens* AK21	GCF_000708045.1	Contig
M_orientalis_1	M. DVO	NZ_SRZX01000001	*Marinobacter orientalis* W62-1	GCF_004792665.1	Contig
M_orientalis_2	M. DVO	NZ_JABCKY010000001	*Marinobacter orientalis* W62-2	GCF_012956055.1	Scaffold
M_oulmenensis	M. DLL	NZ_JACHFE010000001	*Marinobacter oulmenensis* DSM_22359	GCF_014201735.1	Scaffold
M_pelagius_1		NZ_QNRO01000001	*Marinobacter pelagius* 114J	GCF_003315345.1	Scaffold
M_pelagius_2		NZ_FOUR01000016	*Marinobacter pelagius* CGMCC_1.6775	GCF_900114925.1	Contig
M_persicus_1	M. persicus	NZ_PTIU01000001	*Marinobacter persicus* UTICA-S1B9	GCF_002934305.1	Contig
M_persicus_2	M. persicus	NZ_PTIV01000001	*Marinobacter persicus* UTICA-S1B3	GCF_002934325.1	Scaffold
M_persicus_3	M. persicus	NZ_PTIT01000001	*Marinobacter persicus* UTICA-S1B6	GCF_002934485.1	Scaffold
M_persicus_4	M. persicus	NZ_BMYN01000001	*Marinobacter persicus* KCTC_23561	GCF_014652295.1	Scaffold
M_persicus_5	M. persicus	NZ_FOSC01000024	*Marinobacter persicus* IBRC-M_10445	GCF_900114155.1	Scaffold
M_piscensis		NZ_VCGX01000010	*Marinobacter piscensis* Abdou3	GCF_007671655.1	Contig
M_profundi		NZ_KZ319367	*Marinobacter profundi* PWS21	GCF_002744715.1	Scaffold

M_psychrophilus		NZ_CP011494	*Marinobacter psychrophilus* 20041	GCF_001043175.1	Complete Genome
M_salarius_1	M. salarius-I	NZ_CP007152	*Marinobacter salarius* R9SW1	GCF_000831005.1	Complete Genome
M_salarius_2		NZ_CP020931	*Marinobacter salarius* SMR5	GCF_002116735.1	Complete Genome
M_salarius_3		NZ_CP021333	*Marinobacter salarius* HL2708#2	GCF_003986605.1	Complete Genome
M_salarius_4	M. salarius-I	NZ_JAEMOR010000096	*Marinobacter salarius* AT3901	GCF_016461905.1	Contig
M_salarius_5		NZ_JAEMOQ010000098	*Marinobacter salarius* NP2017	GCF_016461915.1	Scaffold
M_salarius_6	M. salarius-I	NZ_QTNR01000010	*Marinobacter salarius* DSW18	GCF_018424725.1	Contig
M_salarius_7	M. salarius-I	NZ_FOTV01000053	*Marinobacter salarius* DSM_26291 (heterotypic synonym *Marinobacter* sp. TT-1)	GCF_900114695.1	Contig
M_salexigens_1	M. salexigens	NZ_NIHC01000001	*Marinobacter salexigens* HJR7	GCF_002806945.1	Contig
M_salexigens_2	M. salexigens	NZ_JAHKPV010000001	*Marinobacter salexigens* D2M19	GCF_018860765.1	Scaffold
M_salicampi		NZ_JAAMPF010000001	*Marinobacter salicampi* ISL-40	GCF_011074795.1	Contig
M_salinus		NZ_CP017715	*Marinobacter salinus* Hb8	GCF_001854125.1	Complete Genome
M_salsuginis_1	M. salsuginis-adhaerens	NZ_BGZH01000001	*Marinobacter salsuginis* 5N-3	GCF_009617755.1	Contig
M_salsuginis_2	M. salsuginis-adhaerens	NZ_BGZI01000001	*Marinobacter salsuginis* NBRC_109114	GCF_009617795.1	Contig
M_salsuginis_3	M. salsuginis-adhaerens	NZ_CP059071	*Marinobacter salsuginis* ECT2AJA-044	GCF_017798185.1	Chromosome
M_salsuginis_4	M. salsuginis-adhaerens	NZ_AOML01000115	*Marinobacter salsuginis* SD_14B	GCF_004936695.1	Contig
M_santoriniensis		NZ_APAT01000001	*Marinobacter santoriniensis* NKSG1	GCF_000347775.1	Contig
M_sediminum		NZ_JAEMQH010000001	*Marinobacter sediminum* R65	GCF_016820575.1	Scaffold
M_segnicrescens	M. DVO	NZ_FOHZ01000081	*Marinobacter segnicrescens* CGMCC_1.6489	GCF_900111555.1	Scaffold
M_shengliensis_1	M. ESH	NZ_PXNO01000001	*Marinobacter shengliensis* SL013A34A2	GCF_003007715.1	Contig
M_shengliensis_2	M. ESH	NZ_SWKL01000011	*Marinobacter shengliensis* subsp. *alexandrii* LZ-6	GCF_005871095.1	Scaffold
M_similis		NZ_CP007151	*Marinobacter similis* A3d10	GCF_000830985.1	Complete Genome
M_sp_1_3A	M. salexigens	NZ_VCGZ01000010	*Marinobacter* sp. 1-3A	GCF_016597775.1	Scaffold

M_sp_1_4A	M. salexigens	NZ_VCGY01000010	*Marinobacter* sp. 1-4A	GCF_016597725.1	Contig
M_sp_3_2	M. salsuginis-adhaerens	NZ_RJUQ01000011	*Marinobacter* sp. 3-2	GCF_003751355.1	Scaffold
M_sp_AC		NZ_MBPP01000001	*Marinobacter* sp. AC-23	GCF_001858325.1	Contig
M_sp_ANT		NZ_NXGV01000006	*Marinobacter* sp. ANT_B65	GCF_002407605.1	Contig
M_sp_Arc7		NZ_CP031848	*Marinobacter* sp. Arc7-DN-1	GCF_003441595.1	Complete Genome
M_sp_bablab_1	M. DLL	NZ_JADARC010000001	*Marinobacter* sp. bablab_jr003	GCF_016464245.1	Contig
M_sp_bablab_2	M_hydrocarbonoclasticus	NZ_JACDWW010000008	*Marinobacter* sp. bablab_jr008	GCF_016464145.1	Contig
M_sp_bablab_3	M. DLL	NZ_JACDWR010000001	*Marinobacter* sp. bablab_jr015	GCF_016463955.1	Contig
M_sp_BSs20148		NC_018268	*Marinobacter* sp. BSs20148	GCF_000283275.1	Complete Genome
M_sp_BW6	M. salsuginis-adhaerens	NZ_VIRN01000043	*Marinobacter* sp. BW6	GCF_008107725.1	Contig
M_sp_C18		NZ_LQXJ01000001	*Marinobacter* sp. C18	GCF_001924925.1	Contig
M_sp_C1S70	M_hydrocarbonoclasticus	NZ_AXBW01000001	*Marinobacter* sp. C1S70	GCF_000475355.1	Contig
M_sp_CAU		NZ_JAHVAO010000001	*Marinobacter* sp. CAU_1620	GCF_019264345.1	Scaffold
M_sp_CHFG3		NZ_JAERVO010000010	*Marinobacter* sp. CHFG3-1-5	GCF_016798325.1	Contig
M_sp_CP1		NZ_CP011929	*Marinobacter* sp. CP1	GCF_001266795.1	Complete Genome
M_sp_DS40M8		NZ_AOMK01001183	*Marinobacter* sp. DS40M8	GCF_004936715.1	Contig
M_sp_DSM_11874	M_hydrocarbonoclasticus	NZ_JACHEV010000001	*Marinobacter* sp. DSM_11874	GCF_014201575.1	Scaffold
M_ sp_DSM_26671		NZ_FONF01000075	*Marinobacter* sp. DSM_26671<br>(heterotypic synonym *Marinobacter* sp. TK-36)	GCF_900112835.1	Scaffold
M_sp_DY40	M. salexigens	NZ_VCHA01000010	*Marinobacter* sp. DY40_1A1	GCF_016597815.1	Contig
M_sp_EC		NZ_LR701485	*Marinobacter* sp. EC-HK377 isolate MBHK15	GCF_902498775.1	Scaffold
M_sp_EhC06	M. salsuginis-adhaerens	NZ_LXYO01000001	*Marinobacter* sp. EhC06	GCF_001650915.1	Contig
M_sp_EhN04	M. salsuginis-adhaerens	NZ_LXYN01000001	*Marinobacter* sp. EhN04	GCF_001650765.1	Contig
M_sp_ELB17		NZ_AAXY01000075	*Marinobacter* sp. ELB17	GCF_000169375.1	Contig

M_sp_EN3	M_hydrocarbonoclasticus	NZ_AXCC01000001	*Marinobacter* sp. EN3	GCF_000475315.1	Contig
M_sp_ES	M. DLL	NZ_AXBV01000001	*Marinobacter* sp. ES-1	GCF_000475255.1	Contig
M_sp_es_042	M. salsuginis-adhaerens	NZ_LT897781	*Marinobacter* sp. es.042	GCF_900188315.1	Chromosome
M_sp_es_048	M. salsuginis-adhaerens	NZ_FYFA01000002	*Marinobacter* sp. es.048	GCF_900188435.1	Contig
M_sp_EVN1	M_hydrocarbonoclasticus	NZ_AXCB01000001	*Marinobacter* sp. EVN1	GCF_000475375.1	Contig
M_sp_F3R08		NZ_JAHKPI010000025	*Marinobacter* sp. F3R08	GCF_018860425.1	Scaffold
M_sp_F3R11		NZ_QOCH01000013	*Marinobacter* sp. F3R11	GCF_003318275.1	Scaffold
M_sp_F4206		NZ_JAHXKI010000001	*Marinobacter* sp. F4206	GCF_019392195.1	Contig
M_sp_F4218		NZ_JAHZIL010000001	*Marinobacter* sp. F4218	GCF_019443085.1	Contig
M_sp_HL_58		NZ_JMLY01000001	*Marinobacter* sp. HL-58	GCF_000686085.1	Contig
M_sp_JB02H27		NZ_VMHN01000010	*Marinobacter* sp. JB02H27	GCF_008795955.1	Contig
M_sp_JB05H06		NZ_JAESIE010000010	*Marinobacter* sp. JB05H06	GCF_016757015.1	Contig
M_sp_JH2	M. DLL	NZ_CP037934	*Marinobacter* sp. JH2	GCF_004353225.1	Complete Genome
M_sp_JSM		NZ_WSSA01000002	*Marinobacter* sp. JSM 1782161	GCF_011421605.1	Contig
M_sp_LPB0319		NZ_CP071247	*Marinobacter* sp. LPB0319	GCF_017301335.1	Complete Genome
M_sp_LQ44	M. ESH	NZ_CP014754	*Marinobacter* sp. LQ44	GCF_001447155.2	Complete Genome
M_sp_LV10MA510		NZ_PDJA01000001	*Marinobacter* sp. LV10MA510-1	GCF_002563885.1	Contig
M_sp_LV10R510_11A		NZ_LT907980	*Marinobacter* sp. LV10R510-11A	GCF_900215155.1	Chromosome
M_sp_LV10R510_8		NZ_PJNC01000001	*Marinobacter* sp. LV10R510-8	GCF_002846515.1	Contig
M_sp_LV10R520		NZ_PDJM01000001	*Marinobacter* sp. LV10R520-4	GCF_002563815.1	Contig
M_sp_lvr2a5a20		NZ_SODW01000001	*Marinobacter* sp. lvr2a5a20	GCF_004365955.1	Contig
M_sp_MC3	M. salsuginis-adhaerens	NZ_JAEMVF010000001	*Marinobacter* sp. MC3	GCF_016758285.1	Contig
M_sp_MCTG268	M. salarius-I	NZ_JQMK01000001	*Marinobacter* sp. MCTG268	GCF_000744695.1	Contig

M_sp_MW3	M. salsuginis-adhaerens	NZ_JAEMVG010000001	*Marinobacter* sp. MW3	GCF_016758325.1	Contig
M_sp_N1		NZ_LR733270	*Marinobacter* sp. N1	GCF_902506385.1	Scaffold
M_sp_NP_4		NZ_CP034142	*Marinobacter* sp. NP-4(2019)	GCF_003994855.1	Complete Genome
M_sp_NP_6		NZ_RPOA01000001	*Marinobacter* sp. NP-6	GCF_003997005.1	Scaffold
M_sp_P4B1	M. DLL	NZ_CM003615	*Marinobacter* sp. P4B1	GCF_001447135.1	Contig
M_sp_PJ_16		NZ_SZYH01000001	*Marinobacter* sp. PJ-16	GCF_005298175.1	Scaffold
M_sp_PT19DW	M. salsuginis-adhaerens	NZ_PZZO01000001	*Marinobacter* sp. PT19DW	GCF_003046275.1	Scaffold
M_sp_R17		NZ_RHGZ01000001	*Marinobacter* sp. R17	GCF_003789045.1	Contig
M_sp_THAF197a	M. ESH	NZ_CP045324	*Marinobacter* sp. THAF197a	GCF_009363275.1	Complete Genome
M_sp_THAF39	M. ESH	NZ_CP045367	*Marinobacter* sp. THAF39	GCF_009363515.1	Complete Genome
M_sp_UBA1922		DDFS01000001.1	*Marinobacter* sp. UBA1922	-	MAG – Contig
M_sp_UBA2498		DDOI01000001.1	*Marinobacter* sp. UBA2498	-	MAG – Contig
M_sp_UBA2504		DDOC01000001.1	*Marinobacter* sp. UBA2504	-	MAG – Contig
M_sp_UBA2678	M. salarius-I	DEME01000001.1	*Marinobacter* sp. UBA2678	-	MAG – Contig
M_sp_UBA2688		DELU01000001.1	*Marinobacter* sp. UBA2688	-	MAG – Contig
M_sp_UBA2698		DELK01000001.1	*Marinobacter* sp. UBA2698	-	MAG – Contig
M_sp_UBA3182		DEXO01000001.1	*Marinobacter* sp. UBA3182	-	MAG – Contig
M_sp_UBA3604	M. salarius-I	DFMB01000001.1	*Marinobacter* sp. UBA3604	-	MAG – Contig
M_sp_UBA3607	M. DLL	DFLY01000001.1	*Marinobacter* sp. UBA3607	-	MAG – Contig

M_sp_UBA4153	M. salsuginis-adhaerens	DFVT01000001.1	*Marinobacter* sp. UBA4153	-	MAG – Contig
M_sp_UBA5687	M_hydrocarbonoclasticus	DIHV01000001.1	*Marinobacter* sp. UBA5687	-	MAG – Contig
M_sp_UBA6499	M_hydrocarbonoclasticus	DJMD01000001.1	*Marinobacter* sp. UBA6499	-	MAG – Contig
M_sp_UBA6604	M_hydrocarbonoclasticus	DJIC01000001.1	*Marinobacter* sp. UBA6604	-	MAG – Contig
M_sp_UBA7807	M. DLL	DLCD01000001.1	*Marinobacter* sp. UBA7807	-	MAG – Contig
M_sp_UBA856	M. DLL	DBGK01000001.1	*Marinobacter* sp. UBA856	-	MAG – Contig
M_sp_X15	M. DLL	NZ_MEIY01000001	*Marinobacter* sp. X15-166B	GCF_001752365.1	Scaffold
M_sp_YJ		NZ_SJDL01000001	*Marinobacter* sp. YJ_S3_2	GCF_004327985.1	Contig
M_sp_YWL01	M. salsuginis-adhaerens	NZ_LORG01000001	*Marinobacter* sp. YWL01	GCF_001601275.1	Contig
M_sp_ZYF650		NZ_VTUU01000001	*Marinobacter* sp. ZYF650	GCF_008370345.1	Contig
M_subterrani		NZ_LFBU01000001	*Marinobacter subterrani* JG233	GCF_001045555.1	Contig
M_vinifirmus	M. DLL	NZ_NEFY01000001	*Marinobacter vinifirmus* FB1	GCF_002258215.1	Contig
M_vulgaris_1	M. DVO	NZ_QFWX01000001	*Marinobacter vulgaris* F01-1	GCF_003344045.1	Scaffold
M_vulgaris_2	M. DVO	NZ_VMBE01000001	*Marinobacter vulgaris* F01-2	GCF_007559285.1	Contig
M_zhanjiangensis	M. DVO	NZ_BMXV01000001	*Marinobacter zhanjiangensis* KCTC_22280	GCF_014651935.1	Scaffold
M_zhejiangensis	M. DLL	NZ_FOUE01000011	*Marinobacter zhejiangensis* CGMCC_1.7061	GCF_900114775.1	Scaffold

Supplementary Data Files

Supplementary Data 1. Mapping coverage and mapping rates of metatranscriptomic libraries recovered by *Colwellia* and *Marinobacter* reference genomes.

Supplementary Data 2. Differentially expressed genes and corresponding available annotation in *Colwellia* and *Marinobacter* CA-Metatranscriptomes.

Supplementary Data 3. Anvio code for *Colwellia* CA-Metatranscriptome.

Supplementary Data 4. Anvio code for *Marinobacter* CA-Metatranscriptome.

References

1. Seidel M, Kleindienst S, Dittmar T, Joye SB, Medeiros PM. Biodegradation of crude oil and dispersants in deep seawater from the Gulf of Mexico: Insights from ultra-high resolution mass spectrometry. *Deep Sea Research Part II: Topical Studies in Oceanography* 2016; **129**: 108–118.
2. Sieradzki ET, Morando M, Fuhrman JA. Metagenomics and Quantitative Stable Isotope Probing Offer Insights into Metabolism of Polycyclic Aromatic Hydrocarbon Degraders in Chronically Polluted Seawater. *mSystems* 2021; **6**: e00245-21.
