# Supplementary material for: Species-specific responses of marine bacteria to environmental perturbation
Source: ISME Commun. 2023 Sep 22;3:99. doi: 10.1038/s43705-023-00310-z (PMC10516948; doi:10.1038/s43705-023-00310-z)
Supplement: Supplementary file 2 — Data Set 3 [file 43705_2023_310_MOESM2_ESM.pdf]

In [ ]:

```
#1
#import sys
#!{sys.executable} -m pip install altair vega_datasets
```

In [1]:

```
#2
import altair as alt
from altair_saver import save
from IPython.display import Image
import numpy as np
import os
import pandas as pd
import re
from scipy import stats
from copy import copy
```

In [2]:

```
#3
def isNaN(num):
    return num != num

def unique(list1):
    x = np.array(list1)
    print(np.unique(x))
```

# *Colwellia* CA-Metatranscriptome

Following http://merenlab.org/2019/03/14/ncbi-genome-download-magic/

Download *Colwellia* genomes from NCBI.

In [3]:

```
#4
!ncbi-genome-download bacteria -l 'all' -g 'Colwellia' -m metadata.txt
```

Process metadata.txt to download NCBI genomes

In [4]:

```
#5
!anvi-script-process-genbank-metadata -m metadata.txt --output-dir NCBI_genomes --output-fasta-txt fasta.txt
 --exclude-gene-calls-from-fasta-txt
```

```
Input metadata file ..........................: metadata.txt
Output directory .............................: /Users/tito_miniconda/JOYE_LAB_ANVIO_PROJECTS/SK_BACKUP/p28_pange
nomes/Colwellia/NCBI_genomes
Num entries in metadata ......................: 69
Output FASTA descriptor ......................: Colwellia.txt[38;5;0m ETA: 0s
```

In [5]:

```
#6
!anvi-run-workflow -w contigs --get-default-config default_config.txt
```

```
WARNING
===============================================
If you publish results from this workflow, please do not forget to cite
snakemake (doi:10.1093/bioinformatics/bts480)


WARNING
===============================================
We are initiating parameters for the contigs workflow

Default config file ..........................: Stored for workflow 'contigs' as
                                                'default_config.txt'.
```

Before running the next line, make sure you add in the `fasta.txt` file those genomes that were not automatically downloaded in the previous line (e.g. SAGs, MAGs, or unpublished genomes).

Here we include three additional MAGs **Colwellia MAG1** Sieradzki et al 2021, **Colwellia MAG2** Campeão, et al 2019, **Colwellia MAG3** Borchert et al 2021

Here it is needed to run the next lines to reduce probability of errors from Anvio

In [24]:

```
#7
```

```
!mkdir GENOMES_OK

#!ls NCBI_genomes | grep fa | awk '{ print "awk %{ if ($0 ~ /^>/) print $0; else {gsub(/[RKWYMSVB]/, %%N%% , $0)

!bash fix1.sh

!mv fasta.txt fasta.txt_v0

!echo -e "name\tpath" > fasta.txt

!ls GENOMES_OK | sed -e 's/\.fa//g' | awk '{print $0 "\t/Users/tito_miniconda/JOYE_LAB_ANVIO_PROJECTS/SK_BACKUP
/p28_pangenomes/Colwellia/GENOMES_OK/\" $0 \".fa\"}' >> fasta.txt
```

Run this, if anvio is newly installed.

```
anvi-setup-scg-taxonomy
```

and

```
anvi-setup-ncbi-cogs
```

Then, the following line was run.

```
anvi-run-workflow -w contigs -c default_config.txt
```

Now creating `external_genomes.txt` file

In [25]:

```
#8
!rm external_genomes.txt
!echo -e 'name\tcontigs_db_path' > external_genomes.txt
!ls ./02_CONTIGS/ | grep '.db' | sed -e "s/-contigs.db//" | awk '{print $0"\t/Users/tito_miniconda/
JOYE_LAB_ANVIO_PROJECTS/SK_BACKUP/p28_pangenomes/Colwellia/02_CONTIGS/\"$0\"-contigs.db\"}' >> external_genomes.txt
```

```
rm: external_genomes.txt: No such file or directory
```

Now creating the database of Colwellia genomes.

In [3]:

```
#9
!anvi-gen-genomes-storage -e external_genomes.txt -o COLWELLIA_GENOMES.db
```

```
                                                                      ETA: None
WARNING
===============================================
Good news! Anvi'o found all these functions that are common to all of your
genomes and will use them for downstream analyses and is very proud of you:
'KEGG_Class, Transfer_RNAs, KEGG_Module, KOfam, COG20_PATHWAY, COG20_CATEGORY,
COG20_FUNCTION'.

Internal genomes .............................: 0 have been initialized.
External genomes .............................: 78 found. 9m ETA: 0s
                                                                     [0m
JUST FYI
===============================================
Some of your genomes had gene calls identified by gene callers other than the
anvi'o default, 'prodigal', and will not be processed. Use the `--debug` flag if
this sounds important and you would like to see more of this message.

                                                                          [0m
* C_MAG1 is stored with 3,174 genes (128 of which were partial)
* C_MAG2 is stored with 3,287 genes (74 of which were partial)          [0m
* C_MAG3 is stored with 3,077 genes (395 of which were partial)         [0m
* C_aestuarii_1 is stored with 3,751 genes (25 of which were partial)   [0m
* C_aestuarii_2 is stored with 3,758 genes (30 of which were partial)   [0m
* C_agarivorans is stored with 4,042 genes (33 of which were partial)   [0m
* C_beringensis is stored with 3,913 genes (1 of which were partial)    [0m
* C_chukchiensis_1 is stored with 3,590 genes (31 of which were partial) [0m
* C_chukchiensis_2 is stored with 3,601 genes (58 of which were partial) [0m
* C_demingiae is stored with 4,447 genes (30 of which were partial)     [0m
* C_echini is stored with 3,484 genes (84 of which were partial)        [0m
* C_hornerae_1 is stored with 3,805 genes (64 of which were partial)    [0m
* C_hornerae_2 is stored with 3,818 genes (102 of which were partial)   [0m
* C_hornerae_3 is stored with 3,807 genes (64 of which were partial)    [0m
* C_marinimaniae is stored with 4,146 genes (516 of which were partial) [0m
* C_mytili is stored with 3,943 genes (25 of which were partial)        [0m
* C_piezophila is stored with 4,525 genes (38 of which were partial)    [0m
* C_polaris is stored with 3,786 genes (23 of which were partial)       [0m
* C_ponticola is stored with 3,339 genes (239 of which were partial)    [0m
* C_psychrerythraea_1 is stored with 4,518 genes (0 of which were partial)0m
* C_psychrerythraea_2 is stored with 4,690 genes (59 of which were partial)m
* C_psychrerythraea_3 is stored with 4,413 genes (30 of which were partial)m
* C_sp_12G3 is stored with 4,214 genes (28 of which were partial)       [0m
* C_sp_20A7 is stored with 3,933 genes (0 of which were partial)        [0m
* C_sp_6M3 is stored with 3,886 genes (234 of which were partial)       [0m
```

```
 * C_sp_75C3 is stored with 4,500 genes (38 of which were partial)        [0m
 * C_sp_Arc7_6 is stored with 4,068 genes (1 of which were partial)       [0m
 * C_sp_Arc7_D is stored with 3,727 genes (1 of which were partial)       [0m
 * C_sp_BRX10_1 is stored with 3,994 genes (123 of which were partial)    [0m
 * C_sp_BRX10_2 is stored with 4,019 genes (164 of which were partial)    [0m
 * C_sp_BRX10_3 is stored with 3,378 genes (40 of which were partial)     [0m
 * C_sp_BRX10_4 is stored with 4,102 genes (147 of which were partial)    [0m
 * C_sp_BRX10_5 is stored with 3,988 genes (121 of which were partial)    [0m
 * C_sp_BRX10_6 is stored with 3,779 genes (179 of which were partial)    [0m
 * C_sp_BRX10_7 is stored with 4,008 genes (133 of which were partial)    [0m
 * C_sp_BRX10_9 is stored with 3,974 genes (130 of which were partial)    [0m
 * C_sp_BRX8_2 is stored with 4,064 genes (230 of which were partial)     [0m
 * C_sp_BRX8_3 is stored with 4,020 genes (134 of which were partial)     [0m
 * C_sp_BRX8_4 is stored with 3,896 genes (78 of which were partial)      [0m
 * C_sp_BRX8_5 is stored with 3,995 genes (78 of which were partial)      [0m
 * C_sp_BRX8_6 is stored with 4,020 genes (140 of which were partial)     [0m
 * C_sp_BRX8_7 is stored with 3,964 genes (125 of which were partial)     [0m
 * C_sp_BRX8_9 is stored with 4,056 genes (70 of which were partial)      [0m
 * C_sp_BRX9_1 is stored with 3,919 genes (82 of which were partial)      [0m
 * C_sp_Bg11_12 is stored with 3,792 genes (64 of which were partial)     [0m
 * C_sp_Bg11_28 is stored with 4,575 genes (9 of which were partial)      [0m
 * C_sp_C1TZA3 is stored with 3,531 genes (99 of which were partial)      [0m
 * C_sp_C2M11 is stored with 3,549 genes (41 of which were partial)       [0m
 * C_sp_D2M02 is stored with 3,447 genes (29 of which were partial)       [0m
 * C_sp_E2M01 is stored with 3,381 genes (31 of which were partial)       [0m
 * C_sp_MB02u_1 is stored with 3,478 genes (41 of which were partial)     [0m
 * C_sp_MB02u_10 is stored with 3,816 genes (101 of which were partial)   [0m
 * C_sp_MB02u_11 is stored with 4,159 genes (126 of which were partial)   [0m
 * C_sp_MB02u_12 is stored with 3,476 genes (43 of which were partial)    [0m
 * C_sp_MB02u_14 is stored with 3,926 genes (56 of which were partial)    [0m
 * C_sp_MB02u_18 is stored with 3,481 genes (43 of which were partial)    [0m
 * C_sp_MB02u_19 is stored with 3,482 genes (48 of which were partial)    [0m
 * C_sp_MB02u_6 is stored with 3,370 genes (19 of which were partial)     [0m
 * C_sp_MB02u_7 is stored with 4,160 genes (138 of which were partial)    [0m
 * C_sp_MB02u_9 is stored with 3,572 genes (65 of which were partial)     [0m
 * C_sp_MB3u_22 is stored with 4,179 genes (152 of which were partial)    [0m
 * C_sp_MB3u_28 is stored with 4,024 genes (42 of which were partial)     [0m
 * C_sp_MB3u_4 is stored with 3,529 genes (51 of which were partial)      [0m
 * C_sp_MB3u_41 is stored with 4,032 genes (44 of which were partial)     [0m
 * C_sp_MB3u_43 is stored with 3,490 genes (55 of which were partial)     [0m
 * C_sp_MB3u_45 is stored with 3,465 genes (41 of which were partial)     [0m
 * C_sp_MB3u_55 is stored with 4,012 genes (84 of which were partial)     [0m
 * C_sp_MB3u_64 is stored with 4,154 genes (119 of which were partial)    [0m
 * C_sp_MB3u_70 is stored with 3,865 genes (63 of which were partial)     [0m
 * C_sp_MB3u_8 is stored with 3,854 genes (41 of which were partial)      [0m
 * C_sp_MT2012 is stored with 3,825 genes (132 of which were partial)     [0m
 * C_sp_MT41 is stored with 3,660 genes (0 of which were partial)         [0m
 * C_sp_OISW is stored with 3,339 genes (239 of which were partial)       [0m
 * C_sp_PAMC_1 is stored with 3,991 genes (2 of which were partial)       [0m
 * C_sp_PAMC_2 is stored with 4,085 genes (1 of which were partial)       [0m
 * C_sp_RSH04 is stored with 3,649 genes (55 of which were partial)       [0m
 * C_sp_TT2012 is stored with 3,987 genes (237 of which were partial)     [0m
 * C_sp_UCD is stored with 3,900 genes (76 of which were partial)         [0m

The new genomes storage ......................: COLWELLIA_GENOMES.db (v7,
                                                 signature: hashdd453a5a)
Number of genomes ............................: 78 (internal: 0, external: 78)
Number of gene calls .........................: 300,653
Number of partial gene calls .................: 6,712
```

Now we can run the pangenomic analysis

```
anvi-pan-genome -g COLWELLIA_GENOMES.db --project-name "Colwellia" --output-dir COLWELLIA --num-threads 6 --minbit 0.5 --mcl-inflation 10 --use-ncbi-blast --enforce-hierarchical-clustering
```

To display the pangenome.

```
anvi-display-pan -p COLWELLIA/Colwellia-PAN.db -g COLWELLIA_GENOMES.db
```

# Selection of a genomic reference

The anvio pangenome analysis doesn't offer to generate a *consensus* reference from a set of genomes. Therefore, if we have 27 metatranscriptomic libraries and 73 genomes in the pangenome, this translates into 27 x 73 = 1971 mapping procedures (and ultimately 1971 BAM files), which is not feasible to plot or to process in a single figure. Therefore we need to chose only one genome as mapping reference for the metapangenomic analysis. We are going to choose the genome that recruits the largest amount of reads from our transcriptomic libraries.

Anvio requires that contig names are the same in the contig database as well as BAM files. To prevent future errors, we are going to export fasta files from the contig databases and they are going to be used for downstream processes.

```
In [ ]: #10
#!ls 02_CONTIGS/*contigs.db | cut -d'/' -f2 | sed -e 's/-contigs.db//'| awk '{print "!anvi-export-contigs
   -c 02_CONTIGS/\" $0 \"-contigs.db -o \" $0 \"_contigs.fa\"}'
#!mkdir NCBI_genomes_v2
#!mv *.fa NCBI_genomes_v2
#Next two lines copy a list of output files from a previous matching of mapping files
#!mkdir 98_SCREENING
#!cp ../Colwellia/98_SCREENING/* ./980_SCREENING
```

```
mkdir 04_MAPPING
```

Then we submit the following script:

```
#!/bin/bash
#PBS -N test
#PBS -q joye_q
#PBS -m abe
#PBS -M montenegrotito@yahoo.com
#PBS -l nodes=1:ppn=1:AMD
#PBS -l walltime=10:00:00
#PBS -l mem=20gb

cd $PBS_O_WORKDIR

module load Bowtie2/2.3.4.1-foss-2016b

bowtie2-build NCBI_genomes_v2/Colwellia_MAG1-contigs.fa 04_MAPPING/Colwellia_MAG1_index
bowtie2-build NCBI_genomes_v2/Colwellia_MAG2-contigs.fa 04_MAPPING/Colwellia_MAG2_index
bowtie2-build NCBI_genomes_v2/Colwellia_MAG3-contigs.fa 04_MAPPING/Colwellia_MAG3_index
bowtie2-build NCBI_genomes_v2/Colwellia_SAG-contigs.fa 04_MAPPING/Colwellia_SAG_index
bowtie2-build NCBI_genomes_v2/Colwellia_ponticola_GCF_005885605_1-contigs.fa
04_MAPPING/Colwellia_ponticola_GCF_005885605_1_index
bowtie2-build NCBI_genomes_v2/Colwellia_sp__6M3_GCF_014077595_1-contigs.fa
04_MAPPING/Colwellia_sp__6M3_GCF_014077595_1_index
bowtie2-build NCBI_genomes_v2/Colwellia_sp__BRX10_1_GCF_014077545_1-contigs.fa
04_MAPPING/Colwellia_sp__BRX10_1_GCF_014077545_1_index
bowtie2-build NCBI_genomes_v2/Colwellia_sp__BRX10_2_GCF_014077515_1-contigs.fa
04_MAPPING/Colwellia_sp__BRX10_2_GCF_014077515_1_index
bowtie2-build NCBI_genomes_v2/Colwellia_sp__BRX10_3_GCF_014077505_1-contigs.fa
04_MAPPING/Colwellia_BRX10_3_GCF_014077505_1_index
bowtie2-build NCBI_genomes_v2/Colwellia_sp__BRX10_4_GCF_014077465_1-contigs.fa
04_MAPPING/Colwellia_sp__BRX10_4_GCF_014077465_1_index
bowtie2-build NCBI_genomes_v2/Colwellia_sp__BRX10_5_GCF_014077435_1-contigs.fa
04_MAPPING/Colwellia_sp__BRX10_5_GCF_014077435_1_index
bowtie2-build NCBI_genomes_v2/Colwellia_sp__BRX10_6_GCF_014077455_1-contigs.fa
04_MAPPING/Colwellia_sp__BRX10_6_GCF_014077455_1_index
bowtie2-build NCBI_genomes_v2/Colwellia_sp__BRX10_7_GCF_014077415_1-contigs.fa
04_MAPPING/Colwellia_sp__BRX10_7_GCF_014077415_1_index
bowtie2-build NCBI_genomes_v2/Colwellia_sp__BRX10_9_GCF_014077405_1-contigs.fa
04_MAPPING/Colwellia_sp__BRX10_9_GCF_014077405_1_index
bowtie2-build NCBI_genomes_v2/Colwellia_sp__BRX8_2_GCF_014077375_1-contigs.fa
04_MAPPING/Colwellia_sp__BRX8_2_GCF_014077375_1_index
bowtie2-build NCBI_genomes_v2/Colwellia_sp__BRX8_3_GCF_014077355_1-contigs.fa
04_MAPPING/Colwellia_sp__BRX8_3_GCF_014077355_1_index
bowtie2-build NCBI_genomes_v2/Colwellia_sp__BRX8_4_GCF_014077345_1-contigs.fa
04_MAPPING/Colwellia_BRX8_4_GCF_014077345_1_index
bowtie2-build NCBI_genomes_v2/Colwellia_sp__BRX8_5_GCF_014077325_1-contigs.fa
04_MAPPING/Colwellia_sp__BRX8_5_GCF_014077325_1_index
bowtie2-build NCBI_genomes_v2/Colwellia_sp__BRX8_6_GCF_014077305_1-contigs.fa
04_MAPPING/Colwellia_sp__BRX8_6_GCF_014077305_1_index
bowtie2-build NCBI_genomes_v2/Colwellia_sp__BRX8_7_GCF_014077275_1-contigs.fa
04_MAPPING/Colwellia_sp__BRX8_7_GCF_014077275_1_index
bowtie2-build NCBI_genomes_v2/Colwellia_sp__BRX8_9_GCF_014077245_1-contigs.fa
04_MAPPING/Colwellia_sp__BRX8_9_GCF_014077245_1_index
bowtie2-build NCBI_genomes_v2/Colwellia_sp__BRX9_1_GCF_014077195_1-contigs.fa
04_MAPPING/Colwellia_sp__BRX9_1_GCF_014077195_1_index
bowtie2-build NCBI_genomes_v2/Colwellia_sp__Bg11_12_GCF_014076955_1-contigs.fa
04_MAPPING/Colwellia_sp__Bg11_12_GCF_014076955_1_index
bowtie2-build NCBI_genomes_v2/Colwellia_sp__C2M11_GCF_018860915_1-contigs.fa
04_MAPPING/Colwellia_sp__C2M11_GCF_018860915_1_index
bowtie2-build NCBI_genomes_v2/Colwellia_sp__D2M02_GCF_018860755_1-contigs.fa
```

```
04_MAPPING/Colwellia_sp__D2M02_GCF_018860755_1_index
bowtie2-build NCBI_genomes_v2/Colwellia_sp__E2M01_GCF_018860585_1-contigs.fa
04_MAPPING/Colwellia_sp__E2M01_GCF_018860585_1_index
bowtie2-build NCBI_genomes_v2/Colwellia_sp__MB02u_10_GCF_014077185_1-contigs.fa
04_MAPPING/Colwellia_sp__MB02u_10_GCF_014077185_1_index
bowtie2-build NCBI_genomes_v2/Colwellia_sp__MB02u_11_GCF_014076925_1-contigs.fa
04_MAPPING/Colwellia_MB02u_11_GCF_014076925_1_index
bowtie2-build NCBI_genomes_v2/Colwellia_sp__MB02u_12_GCF_014077155_1-contigs.fa
04_MAPPING/Colwellia_sp__MB02u_12_GCF_014077155_1_index
bowtie2-build NCBI_genomes_v2/Colwellia_sp__MB02u_14_GCF_014076915_1-contigs.fa
04_MAPPING/Colwellia_sp__MB02u_14_GCF_014076915_1_index
bowtie2-build NCBI_genomes_v2/Colwellia_sp__MB02u_18_GCF_014077125_1-contigs.fa
04_MAPPING/Colwellia_sp__MB02u_18_GCF_014077125_1_index
bowtie2-build NCBI_genomes_v2/Colwellia_sp__MB02u_19_GCF_014077115_1-contigs.fa
04_MAPPING/Colwellia_sp__MB02u_19_GCF_014077115_1_index
bowtie2-build NCBI_genomes_v2/Colwellia_sp__MB02u_1_GCF_014077215_1-contigs.fa
04_MAPPING/Colwellia_sp__MB02u_1_GCF_014077215_1_index
bowtie2-build NCBI_genomes_v2/Colwellia_sp__MB02u_6_GCF_014077095_1-contigs.fa
04_MAPPING/Colwellia_sp__MB02u_6_GCF_014077095_1_index
bowtie2-build NCBI_genomes_v2/Colwellia_sp__MB02u_7_GCF_014076905_1-contigs.fa
04_MAPPING/Colwellia_sp__MB02u_7_GCF_014076905_1_index
bowtie2-build NCBI_genomes_v2/Colwellia_sp__MB02u_9_GCF_014077085_1-contigs.fa
04_MAPPING/Colwellia_sp__MB02u_9_GCF_014077085_1_index
bowtie2-build NCBI_genomes_v2/Colwellia_sp__MB3u_22_GCF_014076885_1-contigs.fa
04_MAPPING/Colwellia_sp__MB3u_22_GCF_014076885_1_index
bowtie2-build NCBI_genomes_v2/Colwellia_sp__MB3u_28_GCF_014076795_1-contigs.fa
04_MAPPING/Colwellia_sp__MB3u_28_GCF_014076795_1_index
bowtie2-build NCBI_genomes_v2/Colwellia_sp__MB3u_41_GCF_014076845_1-contigs.fa
04_MAPPING/Colwellia_sp__MB3u_41_GCF_014076845_1_index
bowtie2-build NCBI_genomes_v2/Colwellia_sp__MB3u_43_GCF_014076995_1-contigs.fa
04_MAPPING/Colwellia_sp__MB3u_43_GCF_014076995_1_index
bowtie2-build NCBI_genomes_v2/Colwellia_sp__MB3u_45_GCF_014077005_1-contigs.fa
04_MAPPING/Colwellia_sp__MB3u_45_GCF_014077005_index
bowtie2-build NCBI_genomes_v2/Colwellia_sp__MB3u_4_GCF_014077065_1-contigs.fa
04_MAPPING/Colwellia_sp__MB3u_4_GCF_014077065_1_index
bowtie2-build NCBI_genomes_v2/Colwellia_sp__MB3u_55_GCF_014076785_1-contigs.fa
04_MAPPING/Colwellia_sp__MB3u_55_GCF_014076785_1_index
bowtie2-build NCBI_genomes_v2/Colwellia_sp__MB3u_64_GCF_014076835_1-contigs.fa
04_MAPPING/Colwellia_sp__MB3u_64_GCF_014076835_1_index
bowtie2-build NCBI_genomes_v2/Colwellia_sp__MB3u_70_GCF_014077045_1-contigs.fa
04_MAPPING/Colwellia_sp__MB3u_70_GCF_014077045_1_index
bowtie2-build NCBI_genomes_v2/Colwellia_sp__MB3u_8_GCF_014076985_1-contigs.fa
04_MAPPING/Colwellia_sp__MB3u_8_GCF_014076985_1_index
```

Next, for each of the transcriptomic libraries we submit a mapping script. This script is in `99_SCRIPTS` as *t-sub.sh_map_MT_2_refgenomes* STDOUT files are located in 98_SCREENING`

In the next lines we are going to process the STDOUT files into a table.

In [28]:

```
# Run in R
#!mkdir 99_SCRIPTS
#!cp /Users/tito_miniconda/JOYE_LAB_ANVIO_PROJECTS/SK_BACKUP/p28_pangenomes/Colwellia/99_SCRIPTS/t-sub.sh_*
   ./99_SCRIPTS/
!ls 98_SCREENING/
```

```
map_OIL11_e.txt map_OIL31_e.txt map_OIL50_e.txt map_OIL70_e.txt map_OIL87_e.txt
map_OIL14_e.txt map_OIL32_e.txt map_OIL53_e.txt map_OIL78_e.txt map_OIL8_e.txt
map_OIL17_e.txt map_OIL34_e.txt map_OIL5_e.txt  map_OIL81_e.txt map_OIL90_e.txt
map_OIL25_e.txt map_OIL37_e.txt map_OIL61_e.txt map_OIL82_e.txt
map_OIL28_e.txt map_OIL44_e.txt map_OIL64_e.txt map_OIL84_e.txt
map_OIL29_e.txt map_OIL47_e.txt map_OIL67_e.txt map_OIL85_e.txt
```

In [29]:

```
map_e_files = ['map_OIL11_e.txt', 'map_OIL32_e.txt', 'map_OIL53_e.txt', 'map_OIL81_e.txt', 'map_OIL14_e.txt', 'map_OIL34_e.txt', 'map_OIL61_e.txt',
'map_OIL82_e.txt', 'map_OIL17_e.txt', 'map_OIL37_e.txt', 'map_OIL64_e.txt', 'map_OIL84_e.txt', 'map_OIL25_e.txt', 'map_OIL44_e.txt', 'map_OIL67_e.txt',
'map_OIL85_e.txt', 'map_OIL28_e.txt', 'map_OIL47_e.txt', 'map_OIL70_e.txt', 'map_OIL87_e.txt', 'map_OIL29_e.txt', 'map_OIL5_e.txt', 'map_OIL78_e.txt',
'map_OIL90_e.txt', 'map_OIL31_e.txt', 'map_OIL50_e.txt', 'map_OIL8_e.txt']
row_names = ['od_0','od_1_2','d_2','o_4_1','d_0','d_1','bc_3','o_4_2','odn_0','odn_1','o_3','od_4_1','bc_1','bc_2','od_3','od_4_2','o_1_1','o_2','d_3',
'd_4','o_1_2','bc_0','bc_4','odn_4','od_1_1','od_2','o_0']
data_mapped = []
data_rates = []
totalreads_vec = []
for e_file in map_e_files:
#file = map_e_files[0]
root = r'/Users/tito_miniconda/JOYE_LAB_ANVIO_PROJECTS/SK_BACKUP/p28_pangenomes/Colwellia/98_SCREENING/'
location = root + e_file
with open(location) as f:
```

```
        lines = f.readlines()
    lines = [x.strip() for x in lines]
    j = 0

    mapped_vec = []
    rates_vec = []
    for i in lines:
        if j == 0:
            fields = i.split(' ')
            total_reads = int(fields[0]) * 2
        if j == 3:
            fields = i.split(' ')
            l1 = int(fields[0]) * 2
        if j == 4:
            fields = i.split(' ')
            l2 = int(fields[0]) * 2
        if j == 7:
            fields = i.split(' ')
            l3 = int(fields[0]) * 2
        if j == 12:
            fields = i.split(' ')
            l4 = int(fields[0])
        if j == 13:
            fields = i.split(' ')
            l5 = int(fields[0])
            mapped = l1 + l2 + l3 + l4 + l5
            mapped_vec.append(mapped)
            rate = mapped * 100 / total_reads
            rates_vec.append(rate)
        if j == 14:
            j = -1
        j = j +1

    totalreads_vec.append(total_reads)
    data_mapped.append(mapped_vec)
    data_rates.append(rates_vec)
```

In [30]:

```
#12
location_script =  r'/Users/tito_miniconda/JOYE_LAB_ANVIO_PROJECTS/SK_BACKUP/p28_pangenomes/Colwellia/99_SCRIPTS/temp.txt'
with open(location_script) as f:
    lines = f.readlines()

lines = [x.strip() for x in lines]

species = []
for i in lines:
    if re.match(r"^bowtie2", i):
        fields = i.split(' ')
        fields0 = fields[5].split('/')
        fields1 = fields0[1].split('_in')
        species.append(fields1[0])
```

In [31]:

```
#13
# Creating pandas DataFrames
mapped_df = pd.DataFrame(data_mapped, columns = species, index=row_names)
maprates_df = pd.DataFrame(data_rates, columns = species, index=row_names)
```

Based on the average of mapped reads per library:

In [32]:

```
#14
mapped_df.mean(axis = 0).sort_values(ascending=False).head(10)
```

Out[32]:

```
Colwellia_MAG2                                  495256.629630
Colwellia_psychrerythraea_34H_GCF_000012325     220714.518519
Colwellia_demingiae_GCF_007954275               219478.074074
Colwellia_sp__Bg11_28_GCF_002836245             218717.333333
Colwellia_psychrerythraea_GCF_000764225         160240.111111
Colwellia_MAG3                                  151193.703704
Colwellia_sp__12G3_GCF_002836775                139843.666667
Colwellia_sp__20A7_GCF_009832865                139278.777778
Colwellia_psychrerythraea_GCF_000764185         136558.703704
Colwellia_sp__75C3_GCF_002836255                134185.666667
dtype: float64
```

```
Colwellia_MAG2                                  495256.629630
Colwellia_psychrerythraea_34H_GCF_000012325     220714.518519
```

```
Colwellia_demingiae_GCF_007954275              219478.074074
Colwellia_sp__Bg11_28_GCF_002836245            218717.333333
Colwellia_psychrerythraea_GCF_000764225        160240.111111
Colwellia_MAG3                                 151193.703704
Colwellia_sp__12G3_GCF_002836775               139843.666667
Colwellia_sp__20A7_GCF_009832865               139278.777778
Colwellia_psychrerythraea_GCF_000764185        136558.703704
Colwellia_sp__75C3_GCF_002836255               134185.666667
dtype: float64
```

Based on the average of the greatest (maximum) number of mapped reads in a library :

In [33]:

```
#15
mapped_df.max(axis = 0).sort_values(ascending=False).head(10)
```

Out[33]:

```
Colwellia_MAG2                                 2219912
Colwellia_psychrerythraea_34H_GCF_000012325     840718
Colwellia_sp__Bg11_28_GCF_002836245             834618
Colwellia_demingiae_GCF_007954275               802817
Colwellia_psychrerythraea_GCF_000764225         591947
Colwellia_MAG3                                  520897
Colwellia_psychrerythraea_GCF_000764185         485183
Colwellia_sp__20A7_GCF_009832865                483193
Colwellia_sp__C2M11_GCF_018860915_1             466352
Colwellia_sp__12G3_GCF_002836775                465563
dtype: int64
```


```
Colwellia_MAG2                                 2219912
Colwellia_psychrerythraea_34H_GCF_000012325     840718
Colwellia_sp__Bg11_28_GCF_002836245             834618
Colwellia_demingiae_GCF_007954275               802817
Colwellia_psychrerythraea_GCF_000764225         591947
Colwellia_MAG3                                  520897
Colwellia_psychrerythraea_GCF_000764185         485183
Colwellia_sp__20A7_GCF_009832865                483193
Colwellia_sp__C2M11_GCF_018860915_1             466352
Colwellia_sp__12G3_GCF_002836775                465563
dtype: int64
```


Based on the average of mapping rates per library:

In [34]:

```
#16
maprates_df.mean(axis = 0).sort_values(ascending=False).head(10)
```

Out[34]:

```
Colwellia_MAG2                                 4.146107
Colwellia_psychrerythraea_34H_GCF_000012325    1.895749
Colwellia_demingiae_GCF_007954275              1.892606
Colwellia_sp__Bg11_28_GCF_002836245            1.878863
Colwellia_psychrerythraea_GCF_000764225        1.403293
Colwellia_MAG3                                 1.339042
Colwellia_sp__20A7_GCF_009832865               1.250246
Colwellia_sp__12G3_GCF_002836775               1.249385
Colwellia_psychrerythraea_GCF_000764185        1.224811
Colwellia_sp__75C3_GCF_002836255               1.198097
dtype: float64
```


```
Colwellia_MAG2                                 4.146107
Colwellia_psychrerythraea_34H_GCF_000012325    1.895749
Colwellia_demingiae_GCF_007954275              1.892606
Colwellia_sp__Bg11_28_GCF_002836245            1.878863
Colwellia_psychrerythraea_GCF_000764225        1.403293
Colwellia_MAG3                                 1.339042
Colwellia_sp__20A7_GCF_009832865               1.250246
Colwellia_sp__12G3_GCF_002836775               1.249385
Colwellia_psychrerythraea_GCF_000764185        1.224811
Colwellia_sp__75C3_GCF_002836255               1.198097
dtype: float64
```


The largest yield of reads recruitment is obtained using the metagenomic assembled genome of ***Candidatus Colwellia aromaticivorans***

Now I export the dataframes to prepare an excel sheet.

In [35]:

```
#17
mapped_df.to_csv('00_Colwellia_mapping_reads.csv')
maprates_df.to_csv('01_Colwellia_mapping_reads_rates.csv')
```

# anvi BAM profile and downstream analysis

Now that we identified the best possible genomic reference, we can proceed to map. First we need the right contigs to do the mapping.

In [45]:

```
#18
!anvi-export-contigs -c 02_CONTIGS/C_MAG2-contigs.db -o C_MAG2_contigs_in_db.fa
###incluster###scp C_MAG2_contigs_in_db.fa tdp56207@sapelo2.gacrc.uga.edu:/scratch/tdp56207/SK/p28/Colwellia
###incluster###sbatch run
```

```
Export mode ..................................: contigs
Output FASTA .................................: C_MAG2_contigs_in_db.fa
```

Check and run `runcell19.sh`

```
#!ls *.tgz | awk '{print "tar xzvf " $0}'



tar xzvf OIL11-RAW.bam.tgz
tar xzvf OIL14-RAW.bam.tgz
tar xzvf OIL17-RAW.bam.tgz
tar xzvf OIL25-RAW.bam.tgz
tar xzvf OIL28-RAW.bam.tgz
tar xzvf OIL29-RAW.bam.tgz
tar xzvf OIL31-RAW.bam.tgz
tar xzvf OIL32-RAW.bam.tgz
tar xzvf OIL34-RAW.bam.tgz
tar xzvf OIL37-RAW.bam.tgz
tar xzvf OIL44-RAW.bam.tgz
tar xzvf OIL47-RAW.bam.tgz
tar xzvf OIL5-RAW.bam.tgz
tar xzvf OIL50-RAW.bam.tgz
tar xzvf OIL53-RAW.bam.tgz
tar xzvf OIL61-RAW.bam.tgz
tar xzvf OIL64-RAW.bam.tgz
tar xzvf OIL67-RAW.bam.tgz
tar xzvf OIL70-RAW.bam.tgz
tar xzvf OIL78-RAW.bam.tgz
tar xzvf OIL8-RAW.bam.tgz
tar xzvf OIL81-RAW.bam.tgz
tar xzvf OIL82-RAW.bam.tgz
tar xzvf OIL84-RAW.bam.tgz
tar xzvf OIL85-RAW.bam.tgz
tar xzvf OIL87-RAW.bam.tgz
tar xzvf OIL90-RAW.bam.tgz



#!ls *.bam | awk '{print "anvi-init-bam "$0" -o " $0 ".bam" }' | sed -e 's/-RAW.bam.bam/.bam/'


anvi-init-bam OIL11-RAW.bam -o OIL11.bam
anvi-init-bam OIL14-RAW.bam -o OIL14.bam
anvi-init-bam OIL17-RAW.bam -o OIL17.bam
anvi-init-bam OIL25-RAW.bam -o OIL25.bam
anvi-init-bam OIL28-RAW.bam -o OIL28.bam
anvi-init-bam OIL29-RAW.bam -o OIL29.bam
anvi-init-bam OIL31-RAW.bam -o OIL31.bam
anvi-init-bam OIL32-RAW.bam -o OIL32.bam
anvi-init-bam OIL34-RAW.bam -o OIL34.bam
anvi-init-bam OIL37-RAW.bam -o OIL37.bam
anvi-init-bam OIL44-RAW.bam -o OIL44.bam
anvi-init-bam OIL47-RAW.bam -o OIL47.bam
anvi-init-bam OIL5-RAW.bam -o OIL5.bam
anvi-init-bam OIL50-RAW.bam -o OIL50.bam
anvi-init-bam OIL53-RAW.bam -o OIL53.bam
```

```
anvi-init-bam OIL61-RAW.bam -o OIL61.bam
anvi-init-bam OIL64-RAW.bam -o OIL64.bam
anvi-init-bam OIL67-RAW.bam -o OIL67.bam
anvi-init-bam OIL70-RAW.bam -o OIL70.bam
anvi-init-bam OIL78-RAW.bam -o OIL78.bam
anvi-init-bam OIL8-RAW.bam -o OIL8.bam
anvi-init-bam OIL81-RAW.bam -o OIL81.bam
anvi-init-bam OIL82-RAW.bam -o OIL82.bam
anvi-init-bam OIL84-RAW.bam -o OIL84.bam
anvi-init-bam OIL85-RAW.bam -o OIL85.bam
anvi-init-bam OIL87-RAW.bam -o OIL87.bam
anvi-init-bam OIL90-RAW.bam -o OIL90.bam



#!ls *.bam | awk '{print "anvi-init-bam "$0" -o " $0 ".bam" }' | sed -e 's/-RAW.bam.bam/.bam/'

anvi-profile -i OIL11.bam --sample-name od_0 -c 02_CONTIGS/C_MAG2-contigs.db
anvi-profile -i OIL14.bam --sample-name d_0  -c 02_CONTIGS/C_MAG2-contigs.db
anvi-profile -i OIL17.bam --sample-name odn_0    -c 02_CONTIGS/C_MAG2-contigs.db
anvi-profile -i OIL25.bam --sample-name bc_1    -c 02_CONTIGS/C_MAG2-contigs.db
anvi-profile -i OIL28.bam --sample-name o_1_1    -c 02_CONTIGS/C_MAG2-contigs.db
anvi-profile -i OIL29.bam --sample-name o_1_2    -c 02_CONTIGS/C_MAG2-contigs.db
anvi-profile -i OIL31.bam --sample-name od_1_1    -c 02_CONTIGS/C_MAG2-contigs.db
anvi-profile -i OIL32.bam --sample-name od_1_2 -c 02_CONTIGS/C_MAG2-contigs.db
anvi-profile -i OIL34.bam --sample-name d_1    -c 02_CONTIGS/C_MAG2-contigs.db
anvi-profile -i OIL37.bam --sample-name odn_1    -c 02_CONTIGS/C_MAG2-contigs.db
anvi-profile -i OIL44.bam --sample-name bc_2    -c 02_CONTIGS/C_MAG2-contigs.db
anvi-profile -i OIL47.bam --sample-name o_2    -c 02_CONTIGS/C_MAG2-contigs.db
anvi-profile -i OIL50.bam --sample-name od_2    -c 02_CONTIGS/C_MAG2-contigs.db
anvi-profile -i OIL53.bam --sample-name d_2 -c 02_CONTIGS/C_MAG2-contigs.db
anvi-profile -i OIL5.bam --sample-name bc_0    -c 02_CONTIGS/C_MAG2-contigs.db
anvi-profile -i OIL61.bam --sample-name bc_3    -c 02_CONTIGS/C_MAG2-contigs.db
anvi-profile -i OIL64.bam --sample-name o_3    -c 02_CONTIGS/C_MAG2-contigs.db
anvi-profile -i OIL67.bam --sample-name od_3    -c 02_CONTIGS/C_MAG2-contigs.db
anvi-profile -i OIL70.bam --sample-name d_3    -c 02_CONTIGS/C_MAG2-contigs.db
anvi-profile -i OIL78.bam --sample-name bc_4    -c 02_CONTIGS/C_MAG2-contigs.db
anvi-profile -i OIL81.bam --sample-name o_4_1 -c 02_CONTIGS/C_MAG2-contigs.db
anvi-profile -i OIL82.bam --sample-name o_4_2    -c 02_CONTIGS/C_MAG2-contigs.db
anvi-profile -i OIL84.bam --sample-name od_4_1    -c 02_CONTIGS/C_MAG2-contigs.db
anvi-profile -i OIL85.bam --sample-name od_4_2    -c 02_CONTIGS/C_MAG2-contigs.db
anvi-profile -i OIL87.bam --sample-name d_4    -c 02_CONTIGS/C_MAG2-contigs.db
anvi-profile -i OIL8.bam --sample-name o_0    -c 02_CONTIGS/C_MAG2-contigs.db
anvi-profile -i OIL90.bam --sample-name odn_4    -c 02_CONTIGS/C_MAG2-contigs.db
```

Don't delete bam files yet. Sorted bam files will be needed in cell 28.

In [5]:

```
#20
!anvi-merge OIL*ANVIO_PROFILE/PROFILE.db -c 02_CONTIGS/C_MAG2-contigs.db -o SAMPLES-MERGED
```

```
                                                                                        [0m
WARNING
===============================================
Anvi'o just set the normalization values for each sample based on how many
mapped reads they contained. This information will only be used to calculate the
normalized coverage table. Here are those values: od_0: 0.32, d_0: 0.17, odn_0:
0.23, bc_1: 0.61, o_1_1: 0.04, o_1_2: 0.07, od_1_1: 0.00, od_1_2: 0.00, d_1:
0.00, odn_1: 0.00, bc_2: 0.39, o_2: 0.09, bc_0: 0.22, od_2: 0.00, d_2: 0.00,
bc_3: 0.22, o_3: 0.12, od_3: 0.01, d_3: 0.00, bc_4: 1.00, o_0: 0.21, o_4_1:
0.13, o_4_2: 0.31, od_4_1: 0.01, od_4_2: 0.02, d_4: 0.00, odn_4: 0.00

profiler_version ............................: 35        ;5;0m..) ETA: \u221e:\u221e:\u221e
output_dir ..................................: /Users/tito_miniconda/JOYE_LAB_ANVIO_PROJECTS/SK_BACKUP/p28_pange
nomes/Colwellia/SAMPLES-MERGED
sample_id ...................................: SAMPLES_MERGED
description .................................: None
profile_db ..................................: /Users/tito_miniconda/JOYE_LAB_ANVIO_PROJECTS/SK_BACKUP/p28_pange
nomes/Colwellia/SAMPLES-MERGED/PROFILE.db
merged ......................................: True
contigs_db_hash .............................: hashbebc968d
num_runs_processed ..........................: 27
merged_sample_ids ...........................: bc_0, bc_1, bc_2, bc_3, bc_4,
                                               d_0, d_1, d_2, d_3, d_4, o_0,
                                               o_1_1, o_1_2, o_2, o_3, o_4_1,
                                               o_4_2, od_0, od_1_1, od_1_2,
                                               od_2, od_3, od_4_1, od_4_2,
```

```
                                                odn_0, odn_1, odn_4
Common layer additional data keys ............: default
total_reads_mapped ...........................: 12151, 4325, 6755, 11919, 2636,
                                                15130, 1088679, 1220834, 929224,
                                                888653, 12508, 67812, 40265,
                                                29471, 22531, 20886, 8633, 8200,
                                                2219912, 1942150, 1611464,
                                                423890, 454428, 136710, 11375,
                                                1340964, 840424
cmd_line .....................................: /Users/tito_miniconda/opt/miniconda3/envs/anvio-7/bin/anvi-merge
 OIL11.bam-ANVIO_PROFILE/PROFILE.db OIL14.bam-ANVIO_PROFILE/PROFILE.db OIL17.bam-ANVIO_PROFILE/PROFILE.db OIL25.b
am-ANVIO_PROFILE/PROFILE.db OIL28.bam-ANVIO_PROFILE/PROFILE.db OIL29.bam-ANVIO_PROFILE/PROFILE.db OIL31.bam-ANVIO
_PROFILE/PROFILE.db OIL32.bam-ANVIO_PROFILE/PROFILE.db OIL34.bam-ANVIO_PROFILE/PROFILE.db OIL37.bam-ANVIO_PROFIL
E/PROFILE.db OIL44.bam-ANVIO_PROFILE/PROFILE.db OIL47.bam-ANVIO_PROFILE/PROFILE.db OIL5.bam-ANVIO_PROFILE/PROFIL
E.db OIL50.bam-ANVIO_PROFILE/PROFILE.db OIL53.bam-ANVIO_PROFILE/PROFILE.db OIL61.bam-ANVIO_PROFILE/PROFILE.db OIL
64.bam-ANVIO_PROFILE/PROFILE.db OIL67.bam-ANVIO_PROFILE/PROFILE.db OIL70.bam-ANVIO_PROFILE/PROFILE.db OIL78.bam-A
NVIO_PROFILE/PROFILE.db OIL8.bam-ANVIO_PROFILE/PROFILE.db OIL81.bam-ANVIO_PROFILE/PROFILE.db OIL82.bam-ANVIO_PROF
ILE/PROFILE.db OIL84.bam-ANVIO_PROFILE/PROFILE.db OIL85.bam-ANVIO_PROFILE/PROFILE.db OIL87.bam-ANVIO_PROFILE/PROF
ILE.db OIL90.bam-ANVIO_PROFILE/PROFILE.db -c 02_CONTIGS/C_MAG2-contigs.db -o SAMPLES-MERGED
clustering_performed .........................: True
                                                                         ;5;0m..) ETA: \u221e:\u221e:\u221e
WARNING
===============================================
Codon frequencies were not profiled, hence, these tables will be empty in the
merged profile database.


                                                                                [0m0m..) ETA: \u221e:\u221e:\u221
e
* Anvi'o hierarchical clustering of contigs...

New items order ..............................: "tnf:euclidean:ward" (type
                                                newick) has been added to the
                                                database...
New items order ..............................: "tnf-cov:euclidean:ward" (type
                                                newick) has been added to the
                                                database...
New items order ..............................: "cov:euclidean:ward" (type
                                                newick) has been added to the
                                                database...


* Additional data and layer orders...

Auxiliary Data ...............................: Found:        [0m
                                                /Users/tito_miniconda/JOYE_LAB_ANVIO_PROJECTS/SK_BACKUP/p28_pange
nomes/Colwellia/SAMPLES-MERGED/AUXILIARY-DATA.db
                                                (v. 2)
Profile Super ................................: Initialized with all 193 splits:
                                                /Users/tito_miniconda/JOYE_LAB_ANVIO_PROJECTS/SK_BACKUP/p28_pange
nomes/Colwellia/SAMPLES-MERGED/PROFILE.db
                                                (v. 35)
                                                                         [0m
Layer orders added
===============================================
* std_coverage
* mean_coverage
* mean_coverage_Q2Q3
* max_normalized_ratio
* relative_abundance
* detection
* abundance
* variability
                                                                         209m ETA: Nonem
Data groups added
===============================================
* default (w/4 items)

* Happy \u2618
```

In [6]:

```
#21
!anvi-export-gene-coverage-and-detection -p SAMPLES-MERGED/PROFILE.db -c 02_CONTIGS/C_MAG2-contigs.db
 -O gene_cov_n_detection.txt
```

```
Auxiliary Data ...............................: Found:        [0m
                                                SAMPLES-MERGED/AUXILIARY-DATA.db
                                                (v. 2)
Profile Super ................................: Initialized with all 193 splits:
                                                SAMPLES-MERGED/PROFILE.db (v.
                                                35)
Gene coverages ...............................: gene_cov_n_detection.txt-GENE-COVERAGES.txt
Gene detection ...............................: gene_cov_n_detection.txt-GENE-DETECTION.txt
```

Here we need to go to display pangenome and we generate a single bin called "EVERYTHING" and then we summarize it

```
anvi-display-pan -p COLWELLIA/Colwellia-PAN.db -g COLWELLIA_GENOMES.db
`
```

In [9]:

```
#22
!anvi-summarize -p COLWELLIA/Colwellia-PAN.db -g COLWELLIA_GENOMES.db -C EVERYTHING -o SUMMARY
#Decompress SUMMARY/Colwellia_gene_clusters_summary.txt.gz
```

```
Genomes storage ...............................................: Initialized[48;5;239m ETA: 0s
                                                                 (storage hash:
                                                                 hashdd453a5a)
Num genomes in storage ........................................: 78
Num genomes will be used ......................................: 78
Pan DB ........................................................: Initialized:
                                                                 COLWELLIA/Colwellia-PAN.db
                                                                 (v. 14)
Gene cluster homogeneity estimates ............................: Functional:
                                                                 [YES]; Geometric:
                                                                 [YES]; Combined:
                                                                 [YES]
                                                                                  [0m
* Gene clusters are initialized for all 31747 gene clusters in the database.

                                                                                  [0m[38;5;0m ETA: 0s
Misc data reported for layers .................................: default
                                                                                  [0m
Misc data reported for items ..................................: default
                                                                                  [0m
HTML Output ...................................................: /Users/tito_miniconda/JOYE_LAB_ANVIO_PROJECTS/SK_B
ACKUP/p28_pangenomes/Colwellia/SUMMARY/index.html
```

The following R script was used to incorporate gene coverage and gene detection profiles from the metatranscriptomes into the pangenome.

```
#!/usr/bin/env Rscript
rm(list=ls());
graphics.off();
setwd("/Users/tito_miniconda/JOYE_LAB_ANVIO_PROJECTS/SK_BACKUP/p28_pangenomes/Colwellia")

#Libraries
library("dplyr")

#Input data

#Gene clusters from the pangenome
gene_clusters_df <- read.table(file = 'SUMMARY/Colwellia_gene_clusters_summary.txt', header =
TRUE, sep = "\t", quote = "")

#Gene coverage profiles from the metatranscriptomic profiles
gene_coverages_df <- read.table(file='gene_cov_n_detection.txt-GENE-COVERAGES.txt', header=TRUE,
sep="\t", quote="")

#Gene detection profiles from the metatranscriptomic profiles
gene_detection_df <- read.table(file = 'gene_cov_n_detection.txt-GENE-DETECTION.txt',
header=TRUE, sep="\t", quote="")

#Let's remember that gene_clusters_df includes entries for all of the genomes stored in the
pangenome.
#At some point we need to subset those gene_callers_id entries that belong to the reference
genome of the
#transcriptomic merged profiles.

#'C_psychrerythraea_1' was the tag utilized for Colwellia psychrerythraea 34H

ref_genome =  'C_MAG2'

#Creating zero dataframes to store processed data
gene_cluster_names <- gene_clusters_df[,colnames(gene_clusters_df) %in% c('gene_cluster_id')]
gene_cluster_names <- unique(gene_cluster_names)
samples_names <- colnames(gene_coverages_df)
samples_names <- samples_names[2:length(samples_names)]
num_cols = length(samples_names)
num_rows = length(gene_cluster_names)
out_coverage_df = data.frame(matrix(0,ncol = num_cols, nrow = num_rows))
colnames(out_coverage_df ) <- samples_names
rownames(out_coverage_df ) <- gene_cluster_names
out_detection_df <- out_coverage_df

#Loop to calculate the maximum coverage and maximum detection observed on each gene cluster.
```

```
for (gene_cluster in unique(gene_clusters_df$gene_cluster_id)){
  df <- gene_clusters_df[gene_clusters_df$gene_cluster_id == gene_cluster, ]
  df2 <- df[df$genome_name == ref_genome,]
  if(length(df2$gene_callers_id) > 0){
    for(my_gene_callers_id in df2$gene_callers_id){
      #For coverages
      df3 <- gene_coverages_df[gene_coverages_df$key == my_gene_callers_id, ]
      x1 <- as.vector(df3)
      x2 <- x1[2:length(x1)]
      #For detections
      df4 <- gene_detection_df[gene_detection_df$key == my_gene_callers_id, ]
      x3 <- as.vector(df4)
      x4 <- x3[2:length(x3)]

      for(i in 1:length(x2)){
        #For coverages
        the_max = max(x2[i],out_coverage_df[gene_cluster,i])
        out_coverage_df[gene_cluster,i] <- the_max
        #For detections
        the_max2 = max(x4[i],out_detection_df[gene_cluster,i])
        out_detection_df[gene_cluster,i] <- the_max2
}}}}

#Adding prefixes and merging dataframes into one
colnames(out_coverage_df) <- paste('cov',colnames(out_coverage_df),sep='_')
colnames(out_detection_df) <- paste('det',colnames(out_detection_df),sep='_')

out_coverage_df <- tibble::rownames_to_column(out_coverage_df, "gene_cluster_id")
out_detection_df <- tibble::rownames_to_column(out_detection_df, "gene_cluster_id")

output_df <- merge(out_coverage_df, out_detection_df,by="gene_cluster_id")

#Export data frame into an anvio friendly table
write.table(output_df, "gene_clusters_additional_data.txt", quote=FALSE, sep="\t",
na="", row.names=FALSE)
```

```
#23
#Next line removes the columns of coverage. Detection columns are enough to be uploaded to anvio.

!cat gene_clusters_additional_data.txt | awk '{print $1,"\t",$29,"\t",$30,"\t",$31,"\t",$32,"\t",$33,"\t",
$34,"\t",$35,"\t",$36, "\t", $37, "\t", $38, "\t", $39, "\t", $40, "\t", $41, "\t", $42,"\t", $43, "\t",
$44, "\t", $45, "\t", $46, "\t", $47, "\t", $48, "\t", $49, "\t",$50, "\t", $51, "\t", $52, "\t", $53, "\t",
$54, "\t", $55}' | tr -d \" \" >
!gene_clusters_additional_data_v2.txt
```

Now let's import our table into the Pangenomic anvio database

In [11]:

```
#24
!anvi-import-misc-data gene_clusters_additional_data_v2.txt -p COLWELLIA/Colwellia-PAN.db
--target-data-table items --just-do-it
```

```
                                                                                [0m
* gene_clusters_additional_data_v2.txt successfully loaded


New data for 'items' in data group 'default'
===============================================
Data key "det_bc_0" ..........................: Predicted type: float
Data key "det_bc_1" ..........................: Predicted type: float
Data key "det_bc_2" ..........................: Predicted type: float
Data key "det_bc_3" ..........................: Predicted type: float
Data key "det_bc_4" ..........................: Predicted type: float
Data key "det_d_0" ...........................: Predicted type: float
Data key "det_d_1" ...........................: Predicted type: float
Data key "det_d_2" ...........................: Predicted type: float
Data key "det_d_3" ...........................: Predicted type: float
Data key "det_d_4" ...........................: Predicted type: float
Data key "det_o_0" ...........................: Predicted type: float
Data key "det_o_1_1" .........................: Predicted type: float
Data key "det_o_1_2" .........................: Predicted type: float
Data key "det_o_2" ...........................: Predicted type: float
Data key "det_o_3" ...........................: Predicted type: float
Data key "det_o_4_1" .........................: Predicted type: float
Data key "det_o_4_2" .........................: Predicted type: float
```

```
Data key "det_od_0" ..........................: Predicted type: float
Data key "det_od_1_1" ........................: Predicted type: float
Data key "det_od_1_2" ........................: Predicted type: float
Data key "det_od_2" ..........................: Predicted type: float
Data key "det_od_3" ..........................: Predicted type: float
Data key "det_od_4_1" ........................: Predicted type: float
Data key "det_od_4_2" ........................: Predicted type: float
Data key "det_odn_0" .........................: Predicted type: float
Data key "det_odn_1" .........................: Predicted type: float
Data key "det_odn_4" .........................: Predicted type: float

                                                                         209m ETA: None
NEW DATA
===============================================
Database .....................................: pan
Data group ...................................: default
Data table ...................................: items
New data keys ................................: det_bc_0, det_bc_1, det_bc_2,
                                                det_bc_3, det_bc_4, det_d_0,
                                                det_d_1, det_d_2, det_d_3,
                                                det_d_4, det_o_0, det_o_1_1,
                                                det_o_1_2, det_o_2, det_o_3,
                                                det_o_4_1, det_o_4_2, det_od_0,
                                                det_od_1_1, det_od_1_2,
                                                det_od_2, det_od_3, det_od_4_1,
                                                det_od_4_2, det_odn_0,
                                                det_odn_1, det_odn_4.
```

In [12]:

```
#25
#Import state

!anvi-import-state -p COLWELLIA/Colwellia-PAN.db -s pan-state.json -n v1
```

```
Done .........................................: State "v1" is added to the
                                                database
```

## Filtering pangenome by mapping recovery

First we want to collapse or split those gene clusters that did not recover transcriptomic reads on *any* library. To do this:

1. Create a bin called `no_signals` and select everything. Tip: Choose a yellow color for this.
2. Create a bin called `T_signals`. Tip: Choose a black color for this.
3. Having the can `T_signals` selected, use the expression `Det X` $> 0$, where `X` is any of the treatments, and then click on append to selected bin. You will have to loop manually through each treatments.
4. Save the bin collection as `bins_by_recovery`.

This would look like this before splitting.

In [57]:

```
Image(filename='Figure_1.jpg')
```

Out[57]:

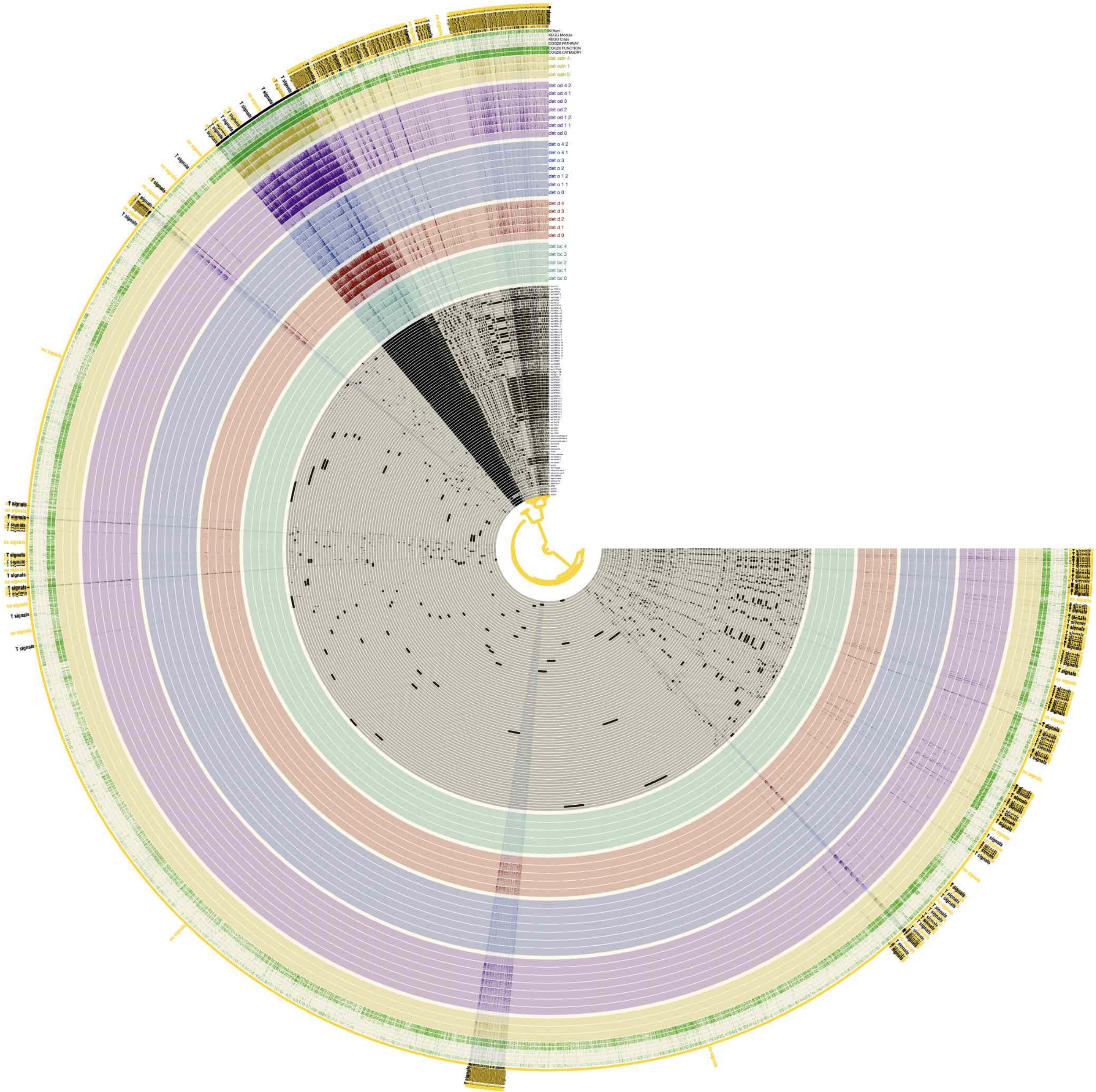


We are going to proceed to split by recovery:

In [14]:

```
#26
!anvi-split -p COLWELLIA/Colwellia-PAN.db -g COLWELLIA_GENOMES.db -C bins_by_recovery -o SPLIT_PANs
```

```
Genomes storage ...............................................: Initialized[48;5;239m ETA: 0s
                                                                  (storage hash:
                                                                  hashdd453a5a)
Num genomes in storage ........................................: 78
Num genomes will be used ......................................: 78
Pan DB ........................................................: Initialized:
                                                                  COLWELLIA/Colwellia-PAN.db
                                                                  (v. 14)
Gene cluster homogeneity estimates ............................: Functional:
                                                                  [YES]; Geometric:
                                                                  [YES]; Combined:
                                                                  [YES]
                                                                                  [0m
* Gene clusters are initialized for all 31747 gene clusters in the database.

                                                                           [0m
WARNING
==============================================================
Anvi'o is about to start splitting your bins into individual, self-contained
anvi'o profiles. This is quite a tricky operation, and even if it finishes
successfully, you must double check everyting in the resulting profiles to make
sure things worked as expected. Although we are doing our best to test all
```

```
these, variation between projects make it impossible to be 100% sure.

Collections .................................: The collection 'DEFAULT' that
                                               describes 2731 splits in a
                                               single bin was successfully
                                               added to the to the database at
                                               'SPLIT_PANs/T_signals/PAN.db'.
                                               Here is a full list of the bin
                                               names in this collection:
                                               ALL_SPLITS.
New items order .............................: "frequency:euclidean:ward" (type
                                               newick) has been added to the
                                               database...
                                                                         [0m
WARNING
===============================================
Clustering for "frequency:euclidean:ward" is already in the database. It will be
replaced with the new content.

New items order .............................: "frequency:euclidean:ward" (type
                                               newick) has been added to the
                                               database...
New items order .............................: "presence-absence:euclidean:ward"
                                               (type newick) has been added to
                                               the database...

WARNING
===============================================================
It seems you have more than 20,000 splits in this particular bin. This is the
soft limit for anvi'o to attempt to create a hierarchical clustering of your
splits (which becomes the center tree in all anvi'o displays). If you want a
hierarchical clustering to be done anyway, you can re-run the splitting process
only for this bin by adding these parameters to your run: '--bin-id no_signals
--enforce-hierarchical-clustering'. If you feel like you are lost, don't
hesitate to get in touch with anvi'o developers.

Collections .................................: The collection 'DEFAULT' that
                                               describes 29016 splits in a
                                               single bin was successfully
                                               added to the to the database at
                                               'SPLIT_PANs/no_signals/PAN.db'.
                                               Here is a full list of the bin
                                               names in this collection:
                                               ALL_SPLITS.
Num bins processed ..........................................: 2
Output directory ............................................: /Users/tito_miniconda/JOYE_LAB_ANVIO_PROJECTS/SK_B
ACKUP/p28_pangenomes/Colwellia/SPLIT_PANs
```

From now on we are interested on the fraction of the pangenome that recovered transcriptomic signals.

```
anvi-display-pan -p SPLIT_PANs/T_signals/PAN.db -g COLWELLIA_GENOMES.db
```

# Differentially Expressed Genes Layers

## Counting mapped reads per gene using htseq

This section I am following https://metagenomics-workshop.readthedocs.io/en/latest/annotation/quantification.html. First we need to extract the a GFF file from the annotation of anvio.

In [4]:

```
#27
!anvi-get-sequences-for-gene-calls -c 02_CONTIGS/C_MAG2-contigs.db -o ref_genome.gff --export-gff3
```

```
Contigs DB ..................................: Initialized:  [0m
                                               02_CONTIGS/C_MAG2-contigs.db (v.
                                               20)

WARNING
===============================================
You did not provide any gene caller ids. As a result, anvi'o will give you back
sequences for every 3353 gene call stored in the contigs database.

                                                                         [0m
THE MORE YOU KNOW \U0001f308
===============================================
Someone asked the Contigs Superclass to initialize only a subset of contig
sequences. Usually this is a good thing and means that some good code somewhere
is looking after you. Just FYI, this class will only know about 101 contig
sequences instead of all the things in the database.

Output ......................................: ref_genome.gff
```

```
In [60]: #28
#Convert gff to gtf format which is compatible with htseq
!cat ref_genome.gff | grep -v "#" | grep "ID=" | cut -f1 -d ';' | sed 's/ID=//g' | cut -f1,4,5,7,9 | awk -v OFS=
 '\\t' '{print $1, \"PRODIGAL\",\"CDS\",$2,$3,\".\",$4,\".\",\"gene_id \" $5}' >  ref_genome.gtf\n"
```

Load htseq in docker.

```
docker pull lbmc/htseq:0.13.5
docker run --oom-kill-disable -v /Users/tito_miniconda/Data:/opt/Data -i -t lbmc/htseq:0.13.5
bash
```

The following lines were run in `runcell29.sh` :

```
!htseq-count -r pos -t CDS -f bam --minaqual 2
/Volumes/Transcend/SK/p28_pangenome/Colwellia/sorted_bam/OIL11.sorted.bam ref_genome.gtf >
od_0_mapcounts.txt
!htseq-count -r pos -t CDS -f bam --minaqual 2
/Volumes/Transcend/SK/p28_pangenome/Colwellia/sorted_bam/OIL14.sorted.bam ref_genome.gtf >
d_0_mapcounts.txt
!htseq-count -r pos -t CDS -f bam --minaqual 2
/Volumes/Transcend/SK/p28_pangenome/Colwellia/sorted_bam/OIL17.sorted.bam ref_genome.gtf >
odn_0_mapcounts.txt
!htseq-count -r pos -t CDS -f bam --minaqual 2
/Volumes/Transcend/SK/p28_pangenome/Colwellia/sorted_bam/OIL25.sorted.bam ref_genome.gtf >
bc_1_mapcounts.txt
!htseq-count -r pos -t CDS -f bam --minaqual 2
/Volumes/Transcend/SK/p28_pangenome/Colwellia/sorted_bam/OIL28.sorted.bam ref_genome.gtf >
o_1_1_mapcounts.txt
!htseq-count -r pos -t CDS -f bam --minaqual 2
/Volumes/Transcend/SK/p28_pangenome/Colwellia/sorted_bam/OIL29.sorted.bam ref_genome.gtf >
o_1_2_mapcounts.txt
!htseq-count -r pos -t CDS -f bam --minaqual 2
/Volumes/Transcend/SK/p28_pangenome/Colwellia/sorted_bam/OIL31.sorted.bam ref_genome.gtf >
od_1_1_mapcounts.txt
!htseq-count -r pos -t CDS -f bam --minaqual 2
/Volumes/Transcend/SK/p28_pangenome/Colwellia/sorted_bam/OIL32.sorted.bam ref_genome.gtf >
od_1_2_mapcounts.txt
!htseq-count -r pos -t CDS -f bam --minaqual 2
/Volumes/Transcend/SK/p28_pangenome/Colwellia/sorted_bam/OIL34.sorted.bam ref_genome.gtf >
d_1_mapcounts.txt
!htseq-count -r pos -t CDS -f bam --minaqual 2
/Volumes/Transcend/SK/p28_pangenome/Colwellia/sorted_bam/OIL37.sorted.bam ref_genome.gtf >
odn_1_mapcounts.txt
!htseq-count -r pos -t CDS -f bam --minaqual 2
/Volumes/Transcend/SK/p28_pangenome/Colwellia/sorted_bam/OIL44.sorted.bam ref_genome.gtf >
bc_2_mapcounts.txt
!htseq-count -r pos -t CDS -f bam --minaqual 2
/Volumes/Transcend/SK/p28_pangenome/Colwellia/sorted_bam/OIL47.sorted.bam ref_genome.gtf >
o_2_mapcounts.txt
!htseq-count -r pos -t CDS -f bam --minaqual 2
/Volumes/Transcend/SK/p28_pangenome/Colwellia/sorted_bam/OIL5.sorted.bam ref_genome.gtf >
bc_0_mapcounts.txt
!htseq-count -r pos -t CDS -f bam --minaqual 2
/Volumes/Transcend/SK/p28_pangenome/Colwellia/sorted_bam/OIL50.sorted.bam ref_genome.gtf >
od_2_mapcounts.txt
!htseq-count -r pos -t CDS -f bam --minaqual 2
/Volumes/Transcend/SK/p28_pangenome/Colwellia/sorted_bam/OIL53.sorted.bam ref_genome.gtf >
d_2_mapcounts.txt
!htseq-count -r pos -t CDS -f bam --minaqual 2
/Volumes/Transcend/SK/p28_pangenome/Colwellia/sorted_bam/OIL61.sorted.bam ref_genome.gtf >
bc_3_mapcounts.txt
!htseq-count -r pos -t CDS -f bam --minaqual 2
/Volumes/Transcend/SK/p28_pangenome/Colwellia/sorted_bam/OIL64.sorted.bam ref_genome.gtf >
o_3_mapcounts.txt
!htseq-count -r pos -t CDS -f bam --minaqual 2
/Volumes/Transcend/SK/p28_pangenome/Colwellia/sorted_bam/OIL67.sorted.bam ref_genome.gtf >
od_3_mapcounts.txt
!htseq-count -r pos -t CDS -f bam --minaqual 2
/Volumes/Transcend/SK/p28_pangenome/Colwellia/sorted_bam/OIL70.sorted.bam ref_genome.gtf >
d_3_mapcounts.txt
!htseq-count -r pos -t CDS -f bam --minaqual 2
/Volumes/Transcend/SK/p28_pangenome/Colwellia/sorted_bam/OIL78.sorted.bam ref_genome.gtf >
```

```
bc_4_mapcounts.txt
!htseq-count -r pos -t CDS -f bam --minaqual 2
/Volumes/Transcend/SK/p28_pangenome/Colwellia/sorted_bam/OIL8.sorted.bam ref_genome.gtf >
o_0_mapcounts.txt
!htseq-count -r pos -t CDS -f bam --minaqual 2
/Volumes/Transcend/SK/p28_pangenome/Colwellia/sorted_bam/OIL81.sorted.bam ref_genome.gtf >
o_4_1_mapcounts.txt
!htseq-count -r pos -t CDS -f bam --minaqual 2
/Volumes/Transcend/SK/p28_pangenome/Colwellia/sorted_bam/OIL82.sorted.bam ref_genome.gtf >
o_4_2_mapcounts.txt
!htseq-count -r pos -t CDS -f bam --minaqual 2
/Volumes/Transcend/SK/p28_pangenome/Colwellia/sorted_bam/OIL84.sorted.bam ref_genome.gtf >
od_4_1_mapcounts.txt
!htseq-count -r pos -t CDS -f bam --minaqual 2
/Volumes/Transcend/SK/p28_pangenome/Colwellia/sorted_bam/OIL85.sorted.bam ref_genome.gtf >
od_4_2_mapcounts.txt
!htseq-count -r pos -t CDS -f bam --minaqual 2
/Volumes/Transcend/SK/p28_pangenome/Colwellia/sorted_bam/OIL87.sorted.bam ref_genome.gtf >
d_4_mapcounts.txt
!htseq-count -r pos -t CDS -f bam --minaqual 2
/Volumes/Transcend/SK/p28_pangenome/Colwellia/sorted_bam/OIL90.sorted.bam ref_genome.gtf >
odn_4_mapcounts.txt
!mkdir 96_htseq_output
!mv *mapcounts.txt 96_htseq_output
```

```
!cut -f4,5,9 ref_genome.gtf | sed 's/gene_id //g' | gawk '{print $3,$2-$1+1}' | tr ' ' '\t' > ref_genome.geneleng
read_files = ['OIL11_f.fa','OIL25_f.fa','OIL31_f.fa','OIL37_f.fa','OIL50_f.fa','OIL61_f.fa','OIL70_f.fa', 'OIL82_f.fa','OIL87_f.fa','OIL11_r.fa','OIL25_r.fa',
'OIL31_r.fa','OIL37_r.fa','OIL50_r.fa','OIL61_r.fa','OIL70_r.fa','OIL82_r.fa','OIL87_r.fa','OIL14_f.fa','OIL28_f.fa','OIL32_f.fa','OIL44_f.fa','OIL53_f.fa',
'OIL64_f.fa','OIL78_f.fa','OIL84_f.fa','OIL8_f.fa','OIL14_r.fa','OIL28_r.fa','OIL32_r.fa','OIL44_r.fa','OIL53_r.fa','OIL64_r.fa','OIL78_r.fa','OIL84_r.fa',
'OIL8_r.fa','OIL17_f.fa','OIL29_f.fa','OIL34_f.fa','OIL47_f.fa','OIL5_f.fa','OIL67_f.fa','OIL81_f.fa','OIL85_f.fa','OIL90_f.fa','OIL17_r.fa','OIL29_r.fa',
'OIL34_r.fa','OIL47_r.fa','OIL5_r.fa','OIL67_r.fa','OIL81_r.fa','OIL85_r.fa','OIL90_r.fa']
sample_ids = ['od_0','bc_1','od_1_1','odn_1','od_2','bc_3','d_3','o_4_2','d_4','od_0','bc_1','od_1_1','odn_1','od_2','bc_3','d_3','o_4_2','d_4','d_0','o_1_1',
'od_1_2','bc_2','d_2','o_3','bc_4','od_4_1','o_0','d_0','o_1_1','od_1_2','bc_2','d_2','o_3','bc_4','od_4_1','o_0','odn_0','o_1_2','d_1','o_2','bc_0','od_3',
'o_4_1','od_4_2','odn_4','odn_0','o_1_2','d_1','o_2','bc_0','od_3','o_4_1','od_4_2','odn_4']
```

The following steps will calculate TPM values for contigs or genes based on count files

TPM values are defined as in Wagner et al (Theory in Biosciences) 2012.

$$TPM_i = \frac{rg*rl*10^6}{f*T}$$

rg: reads mapped to gene g

rl: read length

f: feature length

$T = \sum_i \frac{rg*rl}{f}$ for all $i$ genes

For this calculation we need to know the average read length

In [ ]:

```
#30
# THIS TAKES TIME TO RUN!!!!!!!!!!!!!!!!!!!!!!!!!!!!!!!!!!!!
#root = '/Volumes/Transcend/SK/p01_clean/p03_splited/'
#reads_vec = []
#total_bp_in_lib_vec = []

#for i in read_files:
#    my_location = root + i
#    command1 = 'cat ' + my_location + ' | grep \'^>\' | wc -l'
#    runcommand1 = os.popen(command1)
#    num_reads = runcommand1.read()
#    runcommand1.close()
#    num_reads = num_reads.strip()
#    reads_vec.append(num_reads)
#    command2 = 'cat ' + my_location + ' | grep -v  \'^>\' | awk \'{ print length }\' |
     awk \'{s+=$1}END{print s}\\''
#    runcommand2 = os.popen(command2)
#    total_bp_in_lib = runcommand2.read()
#    runcommand2.close()
#    total_bp_in_lib = total_bp_in_lib.strip()
#    total_bp_in_lib_vec.append(total_bp_in_lib)
```

In [63]:

```
#31
```

```
reads_vec = ['5319504','2599528','8665216','4856793','6533258','2742491','4059579','2650493','7410098','5319504','2599528','8665216','4856793','6533258','2742491','4059579','2650493',
'7410098','3008480','7395105','6898923','3008495','4951454','4587833','3455976','8377386','4570606','3008480','7395105','6898923','3008495','4951454','4587833','3455976','8377386',
'4570606','6753873','6867899','4699125','2359617','4238895','5235701','6756836','2180731','9152171','6753873','6867899','4699125','2359617','4238895','5235701','6756836',
'2180731', ' 9152171']
total_bp_in_lib_vec = ['529556914','258358315','860860737','471734413','637162234','260148861','385237010','247448659','720970318','527979712','257355106','861328463','480966006',
'648081155','270020595','396129359','256912761','728241939','299481143','733713272','684447286','299191768','483932122','434358626','324037321','788966972','455633481','298087834',
'733573535','687327923','298745950','490543446','451526124','335033361','814356799','453712387','667248186','68465238','460414958','232691190','422225840','497983867','640662788',
'203200060','899578610','665617351','681864181','466017852','233762389','420716951','511511621','658014105','211690251','902781824']
read_length_d = {'library': read_files, 'sample_id' : sample_ids, 'num_reads_in_lib' : reads_vec, 'total_bp_in_li
read_length_df = pd.DataFrame(read_length_d)
```

In [64]:

```
read_length_df
```

Out[64]:

| | library | sample_id | num_reads_in_lib | total_bp_in_lib |
|---|---|---|---|---|
| 0 | OIL11_f.fa | od_0 | 5319504 | 529556914 |
| 1 | OIL25_f.fa | bc_1 | 2599528 | 258358315 |
| 2 | OIL31_f.fa | od_1_1 | 8665216 | 860860737 |
| 3 | OIL37_f.fa | odn_1 | 4856793 | 471734413 |
| 4 | OIL50_f.fa | od_2 | 6533258 | 637162234 |
| 5 | OIL61_f.fa | bc_3 | 2742491 | 260148861 |
| 6 | OIL70_f.fa | d_3 | 4059579 | 385237010 |
| 7 | OIL82_f.fa | o_4_2 | 2650493 | 247448659 |
| 8 | OIL87_f.fa | d_4 | 7410098 | 720970318 |
| 9 | OIL11_r.fa | od_0 | 5319504 | 527979712 |
| 10 | OIL25_r.fa | bc_1 | 2599528 | 257355106 |
| 11 | OIL31_r.fa | od_1_1 | 8665216 | 861328463 |
| 12 | OIL37_r.fa | odn_1 | 4856793 | 480966006 |
| 13 | OIL50_r.fa | od_2 | 6533258 | 648081155 |
| 14 | OIL61_r.fa | bc_3 | 2742491 | 270020595 |
| 15 | OIL70_r.fa | d_3 | 4059579 | 396129359 |
| 16 | OIL82_r.fa | o_4_2 | 2650493 | 256912761 |
| 17 | OIL87_r.fa | d_4 | 7410098 | 728241939 |
| 18 | OIL14_f.fa | d_0 | 3008480 | 299481143 |
| 19 | OIL28_f.fa | o_1_1 | 7395105 | 733713272 |
| 20 | OIL32_f.fa | od_1_2 | 6898923 | 684447286 |
| 21 | OIL44_f.fa | bc_2 | 3008495 | 299191768 |
| 22 | OIL53_f.fa | d_2 | 4951454 | 483932122 |
| 23 | OIL64_f.fa | o_3 | 4587833 | 434358626 |
| 24 | OIL78_f.fa | bc_4 | 3455976 | 324037321 |
| 25 | OIL84_f.fa | od_4_1 | 8377386 | 788966972 |
| 26 | OIL8_f.fa | o_0 | 4570606 | 455633481 |
| 27 | OIL14_r.fa | d_0 | 3008480 | 298087834 |
| 28 | OIL28_r.fa | o_1_1 | 7395105 | 733573535 |
| 29 | OIL32_r.fa | od_1_2 | 6898923 | 687327923 |
| 30 | OIL44_r.fa | bc_2 | 3008495 | 298745950 |
| 31 | OIL53_r.fa | d_2 | 4951454 | 490543446 |
| 32 | OIL64_r.fa | o_3 | 4587833 | 451526124 |
| 33 | OIL78_r.fa | bc_4 | 3455976 | 335033361 |
| 34 | OIL84_r.fa | od_4_1 | 8377386 | 814356799 |
| 35 | OIL8_r.fa | o_0 | 4570606 | 453712387 |
| 36 | OIL17_f.fa | odn_0 | 6753873 | 667248186 |
| 37 | OIL29_f.fa | o_1_2 | 6867899 | 684652368 |
| 38 | OIL34_f.fa | d_1 | 4699125 | 460414958 |
| 39 | OIL47_f.fa | o_2 | 2359617 | 232691190 |
| 40 | OIL5_f.fa | bc_0 | 4238895 | 422225840 |
| 41 | OIL67_f.fa | od_3 | 5235701 | 497983867 |

| | library | sample_id | num_reads_in_lib | total_bp_in_lib |
|---|---|---|---|---|
| 42 | OIL81_f.fa | o_4_1 | 6756836 | 640662788 |
| 43 | OIL85_f.fa | od_4_2 | 2180731 | 203200060 |
| 44 | OIL90_f.fa | odn_4 | 9152171 | 899578610 |
| 45 | OIL17_r.fa | odn_0 | 6753873 | 665617351 |
| 46 | OIL29_r.fa | o_1_2 | 6867899 | 681864181 |
| 47 | OIL34_r.fa | d_1 | 4699125 | 466017852 |
| 48 | OIL47_r.fa | o_2 | 2359617 | 233762389 |
| 49 | OIL5_r.fa | bc_0 | 4238895 | 420716951 |
| 50 | OIL67_r.fa | od_3 | 5235701 | 511511621 |
| 51 | OIL81_r.fa | o_4_1 | 6756836 | 658014105 |
| 52 | OIL85_r.fa | od_4_2 | 2180731 | 211690251 |
| 53 | OIL90_r.fa | odn_4 | 9152171 | 902781824 |

In [65]:

```
#32
read_length_df_v2 = read_length_df[['sample_id','num_reads_in_lib', 'total_bp_in_lib']]
read_length_df_v2[['num_reads_in_lib', 'total_bp_in_lib']] = read_length_df_v2[['num_reads_in_lib',
'total_bp_in_lib']].astype('int64')
read_length_df_v2 = read_length_df_v2.groupby(['sample_id']).sum()
read_length_df_v2['avg_read_length'] = read_length_df_v2['total_bp_in_lib']/read_length_df_v2['num_reads_in_lib']
read_length_df_v2
```

```
/Users/tito_miniconda/opt/miniconda3/envs/anvio-7/lib/python3.6/site-packages/pandas/core/frame.py:3494: SettingW
ithCopyWarning:
A value is trying to be set on a copy of a slice from a DataFrame.
Try using .loc[row_indexer,col_indexer] = value instead

See the caveats in the documentation: http://pandas.pydata.org/pandas-docs/stable/user_guide/indexing.html#return
ing-a-view-versus-a-copy
  self[k1] = value[k2]
```

Out[65]:

| | num_reads_in_lib | total_bp_in_lib | avg_read_length |
|---|---|---|---|
| **sample_id** | | | |
| bc_0 | 8477790 | 842942791 | 99.429544 |
| bc_1 | 5199056 | 515713421 | 99.193665 |
| bc_2 | 6016990 | 597937718 | 99.374890 |
| bc_3 | 5484982 | 530169456 | 96.658377 |
| bc_4 | 6911952 | 659070682 | 95.352323 |
| d_0 | 6016960 | 597568977 | 99.314102 |
| d_1 | 9398250 | 926432810 | 98.575034 |
| d_2 | 9902908 | 974475568 | 98.402971 |
| d_3 | 8119158 | 781366369 | 96.237365 |
| d_4 | 14820196 | 1449212257 | 97.786308 |
| o_0 | 9141212 | 909345868 | 99.477604 |
| o_1_1 | 14790210 | 1467286807 | 99.206624 |
| o_1_2 | 13735798 | 1366516549 | 99.485778 |
| o_2 | 4719234 | 466453579 | 98.840952 |
| o_3 | 9175666 | 885884750 | 96.547188 |
| o_4_1 | 13513672 | 1298676893 | 96.100963 |
| o_4_2 | 5300986 | 504361420 | 95.144832 |
| od_0 | 10639008 | 1057536626 | 99.401808 |
| od_1_1 | 17330432 | 1722189200 | 99.373703 |
| od_1_2 | 13797846 | 1371775209 | 99.419519 |
| od_2 | 13066516 | 1285243389 | 98.361598 |
| od_3 | 10471402 | 1009495488 | 96.404998 |
| od_4_1 | 16754772 | 1603323771 | 95.693559 |

| | num_reads_in_lib | total_bp_in_lib | avg_read_length |
|---|---|---|---|
| **sample_id** | | | |
| od_4_2 | 4361462 | 414890311 | 95.126430 |
| odn_0 | 13507746 | 1332865537 | 98.674163 |
| odn_1 | 9713586 | 952700419 | 98.079167 |
| odn_4 | 18304342 | 1802360434 | 98.466278 |

In [126…

```
#33
sample_names = pd.Series(read_length_df_v2.index.values)
my_lengths = r'/Users/tito_miniconda/JOYE_LAB_ANVIO_PROJECTS/SK_BACKUP/p28_pangenomes/Colwellia/
ref_genome.genelengths'
gene_lengths = pd.read_table(my_lengths, header=None, index_col=0, names=['gene_id','gene_length'])
root = r'/Users/tito_miniconda/JOYE_LAB_ANVIO_PROJECTS/SK_BACKUP/p28_pangenomes/Colwellia/96_htseq_output/'
my_tpm_df = pd.DataFrame()
for i in np.arange(sample_names.size):

    counts_file = sample_names[i] + '_mapcounts.txt'
    location = root + counts_file
    rg = pd.read_table(location, header=None, index_col=0, names=['gene_id', 'count'])
    rg_v2 = rg.drop(rg.tail(5).index)
    ## Intersect with genes in the gene length file
    rg_v3 = rg_v2.iloc[list(set(gene_lengths.index).intersection(set(rg_v2.index.astype('int64'))))]
    gene_lengths_v2 = gene_lengths.iloc[list(rg_v3.index)]
    rg_v3.index = rg_v3.index.astype('int64')
    gene_lengths_v2.index = gene_lengths_v2.index.astype('int64')
    ## Average read length for sample
    rl = read_length_df_v2.at[sample_names[i],'avg_read_length']
    ## Calculate T for sample
    T = np.sum(rl * rg_v3['count'].divide(gene_lengths_v2['gene_length']))
    ## Calculate TPM for sample
    tpm = (1e6*rl/T)*rg_v3['count'].divide(gene_lengths_v2['gene_length'])
    ## Create dataframe
    tpm_se = pd.DataFrame(tpm, columns=[sample_names[i]])
    ## Concatenate to results
    my_tpm_df = pd.concat([my_tpm_df,tpm_se],axis=1)
```

In [131…

```
#This is to check we are not getting a matrix full of zeros
my_tpm_df[my_tpm_df.sum(axis=1) > 0]
```

Out[131…

| | bc_0 | bc_1 | bc_2 | bc_3 | bc_4 | d_0 | d_1 | d_2 | |
|---|---|---|---|---|---|---|---|---|---|
| **gene_id** | | | | | | | | | |
| 1006 | 12652.843639 | 40094.407098 | 12308.615804 | 17819.007019 | 22465.582319 | 15559.033140 | 8057.822544 | 8826.814223 | 9854.70[illegible] |
| 1021 | 47626.876126 | 97517.752857 | 37421.321352 | 38695.936853 | 0.000000 | 40545.785513 | 32816.764084 | 29919.371442 | 33915.44[illegible] |
| 1035 | 0.000000 | 0.000000 | 0.000000 | 0.000000 | 0.000000 | 0.000000 | 2433.417362 | 1722.121889 | 1132.74[illegible] |
| 1051 | 0.000000 | 0.000000 | 0.000000 | 22548.743450 | 0.000000 | 0.000000 | 16158.589700 | 13193.236593 | 3224.30[illegible] |
| 1128 | 0.000000 | 0.000000 | 0.000000 | 0.000000 | 0.000000 | 4707.899327 | 199.849207 | 72.577287 | 170.06[illegible] |
| ... | ... | ... | ... | ... | ... | ... | ... | ... | |
| 812 | 0.000000 | 0.000000 | 0.000000 | 0.000000 | 0.000000 | 0.000000 | 290.397507 | 580.034563 | 123.56[illegible] |
| 877 | 0.000000 | 0.000000 | 0.000000 | 0.000000 | 0.000000 | 0.000000 | 1019.844963 | 506.818199 | 30.45[illegible] |
| 938 | 1125.795549 | 0.000000 | 0.000000 | 0.000000 | 0.000000 | 0.000000 | 2291.890484 | 1075.618072 | 780.14[illegible] |
| 951 | 0.000000 | 0.000000 | 0.000000 | 0.000000 | 0.000000 | 0.000000 | 1152.322024 | 2358.691632 | 2080.07[illegible] |
| 953 | 0.000000 | 0.000000 | 0.000000 | 0.000000 | 0.000000 | 0.000000 | 337.790864 | 490.688850 | 63.87[illegible] |

102 rows × 27 columns

In [132…

```
#34
my_tpm_df.to_csv('tpm.csv', sep='\t')
```

# DESeq in R

Please close this notebook and open the notebook `DE_Analysis.ipynb` to run the differential expression analysis.

# Load DE profiles and convert from gene to gene_cluster table

In [16]:

```
#35
!sqlite3 -header -csv SPLIT_PANs/T_signals/PAN.db "select * from gene_cluster_frequencies;" >
pan_split_gene_clusters.csv
!mv pan_split_gene_clusters.csv SPLIT_PANs/
pan_split_geneclusters_df = pd.read_csv(r'
/Users/tito_miniconda/JOYE_LAB_ANVIO_PROJECTS/SK_BACKUP/p28_pangenomes/Colwellia/SPLIT_PANs/pan_split_gene_clusters.csv')
DESEQ_df = pd.read_csv(r'/Users/tito_miniconda/JOYE_LAB_ANVIO_PROJECTS/SK_BACKUP/p28_pangenomes/Colwellia/Colwellia_DE_df.txt', sep='\\t')
sig_df = DESEQ_df[DESEQ_df.padj < 0.05]
sig_df.rename(columns={'Gene':'gene_caller_id'}, inplace=True)


#Building output scheme
DE_for_anvio_df = pan_split_geneclusters_df.iloc[:,0:9]
DE_for_anvio_df.columns= ['gene_cluster_id', 'A', 'B', 'C', 'D', 'E', 'F', 'G', 'H',]
DE_for_anvio_df.A = 0
DE_for_anvio_df.B = 0
DE_for_anvio_df.C = 0
DE_for_anvio_df.D = 0
DE_for_anvio_df.E = 0
DE_for_anvio_df.F = 0
DE_for_anvio_df.G = 0
DE_for_anvio_df.H = 0
DE_for_anvio_df.columns= ['gene_cluster_id', 'DE_d!A', 'DE_d!B', 'DE_o!A', 'DE_o!B', 'DE_od!A', 'DE_od!B',
'DE_od 'DE_odn!A', 'DE_odn!B',]
DE_for_anvio_df = DE_for_anvio_df.drop_duplicates()
```

```
/Users/tito_miniconda/opt/miniconda3/envs/anvio-7/lib/python3.6/site-packages/pandas/core/frame.py:4223: SettingW
ithCopyWarning:
A value is trying to be set on a copy of a slice from a DataFrame

See the caveats in the documentation: http://pandas.pydata.org/pandas-docs/stable/user_guide/indexing.html#return
ing-a-view-versus-a-copy
  return super().rename(**kwargs)
```

In [17]:

```
sig_df
```

Out[17]:

| | gene_caller_id | baseMean | log2FoldChange | lfcSE | stat | pvalue | padj | treatment | abs_diff_mean |
|---|---|---|---|---|---|---|---|---|---|
| 2 | 1 | 20.652821 | 7.058557 | 2.526693 | 2.793595 | 0.005213 | 0.014452 | d | 36 |
| 3 | 10 | 33.816041 | 4.022074 | 1.783657 | 2.254960 | 0.024136 | 0.046047 | d | 63 |
| 4 | 100 | 24.916768 | 5.162786 | 2.149618 | 2.401723 | 0.016318 | 0.033417 | d | 41 |
| 8 | 1003 | 44.869499 | 5.699356 | 2.038074 | 2.796443 | 0.005167 | 0.014370 | d | 84 |
| 10 | 1005 | 19.846114 | 6.892983 | 1.954851 | 3.526092 | 0.000422 | 0.003991 | d | 37 |
| ... | ... | ... | ... | ... | ... | ... | ... | ... | ... |
| 9255 | 955 | 24.697529 | 7.025976 | 2.371628 | 2.962512 | 0.003051 | 0.011798 | odn | 57 |
| 9258 | 96 | 40.131467 | 4.386142 | 1.807318 | 2.426879 | 0.015229 | 0.035144 | odn | 70 |
| 9264 | 972 | 13.717658 | 6.980861 | 2.469230 | 2.827141 | 0.004697 | 0.014939 | odn | 32 |
| 9267 | 977 | 11.138398 | 6.643899 | 2.921423 | 2.274200 | 0.022954 | 0.048819 | odn | 21 |
| 9271 | 99 | 32.684410 | 7.950460 | 2.387600 | 3.329896 | 0.000869 | 0.006517 | odn | 64 |

4519 rows × 9 columns

```
#35
#!sqlite3 -header -csv SPLIT_PANs/T_signals/PAN.db \"select * from gene_clusters;\" > pan_split_gene_clusters.csv
!sqlite3 -header -csv SPLIT_PANs/T_signals/PAN.db \"select * from gene_cluster_frequencies;\" >
pan_split_gene_clusters.csv
!mv pan_split_gene_clusters.csv SPLIT_PANs/
pan_split_geneclusters_df = pd.read_csv(r'/Users/tito_miniconda/JOYE_LAB_ANVIO_PROJECTS/SK_BACKUP/p28_pangenomes/
Colwellia/SPLIT_PANs/pan_split_gene_clusters.csv')
#pan_split_geneclusters_df = pan_split_geneclusters_df[pan_split_geneclusters_df.genome_name == 'C_psychrerythraea_1']
DESEQ_df = pd.read_csv(r'/Users/tito_miniconda/JOYE_LAB_ANVIO_PROJECTS/SK_BACKUP/p28_pangenomes/Colwellia/
Colwellia_DE_df.txt', sep='\\t')
#sig_df = DESEQ_df[((DESEQ_df.padj < 0.05) & (DESEQ_df.zeros_in_bc < 3)) | (DESEQ_df.t_test_p_value < 0.05)]
sig_df = DESEQ_df[DESEQ_df.padj < 0.05]
sig_df.rename(columns={'Gene':'gene_caller_id'}, inplace=True)
```

In [18]:

```
DE_for_anvio_df
```

Out[18]:

| | gene_cluster_id | DE_d!A | DE_d!B | DE_o!A | DE_o!B | DE_od!A | DE_od!B | DE_odn!A | DE_odn!B |
|---|---|---|---|---|---|---|---|---|---|
| 0 | GC_00001106 | 0 | 0 | 0 | 0 | 0 | 0 | 0 | 0 |

| | gene_cluster_id | DE_d!A | DE_d!B | DE_o!A | DE_o!B | DE_od!A | DE_od!B | DE_odn!A | DE_odn!B |
|---|---|---|---|---|---|---|---|---|---|
| 1 | GC_00000435 | 0 | 0 | 0 | 0 | 0 | 0 | 0 | 0 |
| 2 | GC_00000717 | 0 | 0 | 0 | 0 | 0 | 0 | 0 | 0 |
| 3 | GC_00002004 | 0 | 0 | 0 | 0 | 0 | 0 | 0 | 0 |
| 4 | GC_00003876 | 0 | 0 | 0 | 0 | 0 | 0 | 0 | 0 |
| ... | ... | ... | ... | ... | ... | ... | ... | ... | ... |
| 2726 | GC_00008247 | 0 | 0 | 0 | 0 | 0 | 0 | 0 | 0 |
| 2727 | GC_00000041 | 0 | 0 | 0 | 0 | 0 | 0 | 0 | 0 |
| 2728 | GC_00005846 | 0 | 0 | 0 | 0 | 0 | 0 | 0 | 0 |
| 2729 | GC_00001351 | 0 | 0 | 0 | 0 | 0 | 0 | 0 | 0 |
| 2730 | GC_00002791 | 0 | 0 | 0 | 0 | 0 | 0 | 0 | 0 |

2731 rows × 9 columns

In [19]:
```
#36
#Merging annotation data with DE data
!sqlite3 -header -csv SPLIT_PANs/T_signals/PAN.db "select * from gene_clusters;" > pan_split_gene_clusters_v2.csv
!mv pan_split_gene_clusters_v2.csv SPLIT_PANs/
pan_split_geneclusters_df_v2 = pd.read_csv(r'/Users/tito_miniconda/JOYE_LAB_ANVIO_PROJECTS/SK_BACKUP/p28_pangenomes/Colwellia/
SPLIT_PANs/pan_split_gene_clusters_v2.csv')
pan_split_geneclusters_df_v2 = pan_split_geneclusters_df_v2[pan_split_geneclusters_df_v2.genome_name == 'C_MAG2']
```

In [20]:
```
#This number should be bigger than next cell
len(pan_split_geneclusters_df_v2.gene_caller_id.unique())
```

Out[20]: 2874

In [21]:
```
len(sig_df.gene_caller_id.unique())
```

Out[21]: 1658

In [22]:
```
#37
temp_df = pd.merge(pan_split_geneclusters_df_v2,sig_df,on='gene_caller_id',how='left')
#Print description
temp_df.to_csv(r'/Users/tito_miniconda/JOYE_LAB_ANVIO_PROJECTS/SK_BACKUP/p28_pangenomes/Colwellia/
Colwellia_DE_w_description.txt', index = False, sep='\\t')
```

In [23]:
```
#38
for index,row in DE_for_anvio_df.iterrows():
    #print(row[0])
    #my_genecluster_id = 'GC_00001516'
    my_genecluster_id = row[0]
    temp2_df = temp_df[temp_df.gene_cluster_id == my_genecluster_id]
    if not temp2_df.empty:
        up_df = temp2_df[temp2_df.log2FoldChange > 0]
        if not up_df.empty:
            for index2,row2 in up_df.iterrows():
                if row2[10] == 'd':
                    DE_for_anvio_df.at[index,'DE_d!B'] = DE_for_anvio_df.loc[index,'DE_d!B'] + 1
                if row2[10] == 'o':
                    DE_for_anvio_df.at[index,'DE_o!B'] = DE_for_anvio_df.loc[index,'DE_o!B'] + 1
                if row2[10] == 'od':
                    DE_for_anvio_df.at[index,'DE_od!B'] = DE_for_anvio_df.loc[index,'DE_od!B'] + 1
                if row2[10] == 'odn':
                    DE_for_anvio_df.at[index,'DE_odn!B'] = DE_for_anvio_df.loc[index,'DE_odn!B'] + 1
        down_df = temp2_df[temp2_df.log2FoldChange < 0]
        if not down_df.empty:
            for index3,row2 in down_df.iterrows():
                if row2[10] == 'd':
                    DE_for_anvio_df.at[index,'DE_d!A'] = DE_for_anvio_df.loc[index,'DE_d!A'] + 1
                if row2[10] == 'o':
                    DE_for_anvio_df.at[index,'DE_o!A'] = DE_for_anvio_df.loc[index,'DE_o!A'] + 1
                if row2[10] == 'od':
                    DE_for_anvio_df.at[index,'DE_od!A'] = DE_for_anvio_df.loc[index,'DE_od!A'] + 1
                if row2[10] == 'odn':
                    DE_for_anvio_df.at[index,'DE_odn!A'] = DE_for_anvio_df.loc[index,'DE_odn!A'] + 1
```

In [24]:
```
DE_for_anvio_df
```

Out[24]:

| | gene_cluster_id | DE_d!A | DE_d!B | DE_o!A | DE_o!B | DE_od!A | DE_od!B | DE_odn!A | DE_odn!B |
|---|---|---|---|---|---|---|---|---|---|
| 0 | GC_00001106 | 0 | 1 | 0 | 1 | 0 | 1 | 0 | 1 |
| 1 | GC_00000435 | 0 | 0 | 0 | 0 | 0 | 0 | 0 | 0 |
| 2 | GC_00000717 | 0 | 1 | 0 | 0 | 0 | 1 | 0 | 1 |
| 3 | GC_00002004 | 0 | 1 | 0 | 0 | 0 | 0 | 0 | 0 |
| 4 | GC_00003876 | 0 | 1 | 0 | 0 | 0 | 0 | 0 | 1 |
| ... | ... | ... | ... | ... | ... | ... | ... | ... | ... |
| 2726 | GC_00008247 | 0 | 1 | 0 | 0 | 0 | 1 | 0 | 1 |
| 2727 | GC_00000041 | 0 | 0 | 0 | 0 | 0 | 0 | 0 | 0 |
| 2728 | GC_00005846 | 0 | 1 | 0 | 0 | 0 | 1 | 0 | 1 |
| 2729 | GC_00001351 | 0 | 0 | 0 | 0 | 0 | 0 | 0 | 0 |
| 2730 | GC_00002791 | 0 | 0 | 0 | 0 | 0 | 0 | 0 | 0 |

2731 rows × 9 columns

In [25]:
```
DE_for_anvio_df[DE_for_anvio_df.sum(axis=1) > 0]
```

Out[25]:

| | gene_cluster_id | DE_d!A | DE_d!B | DE_o!A | DE_o!B | DE_od!A | DE_od!B | DE_odn!A | DE_odn!B |
|---|---|---|---|---|---|---|---|---|---|
| 0 | GC_00001106 | 0 | 1 | 0 | 1 | 0 | 1 | 0 | 1 |
| 2 | GC_00000717 | 0 | 1 | 0 | 0 | 0 | 1 | 0 | 1 |
| 3 | GC_00002004 | 0 | 1 | 0 | 0 | 0 | 0 | 0 | 0 |
| 4 | GC_00003876 | 0 | 1 | 0 | 0 | 0 | 0 | 0 | 1 |
| 9 | GC_00001049 | 0 | 1 | 0 | 0 | 0 | 1 | 0 | 1 |
| ... | ... | ... | ... | ... | ... | ... | ... | ... | ... |
| 2722 | GC_00031283 | 0 | 1 | 0 | 0 | 0 | 1 | 0 | 1 |
| 2724 | GC_00020653 | 0 | 1 | 0 | 0 | 0 | 0 | 0 | 1 |
| 2725 | GC_00000674 | 0 | 0 | 0 | 0 | 0 | 1 | 0 | 0 |
| 2726 | GC_00008247 | 0 | 1 | 0 | 0 | 0 | 1 | 0 | 1 |
| 2728 | GC_00005846 | 0 | 1 | 0 | 0 | 0 | 1 | 0 | 1 |

1598 rows × 9 columns

In [26]:
```
#It is necessary to check for empty columns. Empty columns will generate errors in Anvio stackbars
DE_for_anvio_df.sum()
#   DE_odn!A has 0 DE genes.
```

Out[26]:
```
gene_cluster_id    GC_00001106GC_00000435GC_00000717GC_00002004GC...
DE_d!A                                                             4
DE_d!B                                                          1457
DE_o!A                                                             4
DE_o!B                                                           634
DE_od!A                                                            3
DE_od!B                                                         1137
DE_odn!A                                                           0
DE_odn!B                                                        1225
dtype: object
```

In [27]:
```
DE_for_anvio_df.columns= ['gene_cluster_id', 'DE_d!A', 'DE_d!B', 'DE_o!A', 'DE_o!B', 'DE_od!A', 'DE_od!B',
'DE_odn_A', 'DE_odn_B',]
```

In [28]:
```
DE_for_anvio_df.DE_odn_B.max()
```

Out[28]:
```
7
```

In [29]:
```
#DE table able to be loaded into anvio
DE_for_anvio_df.to_csv(r'/Users/tito_miniconda/JOYE_LAB_ANVIO_PROJECTS/SK_BACKUP/p28_pangenomes/Colwellia/
Colwellia_DE_for_anvio.txt', index = False, sep='\\t')
```

In [30]:

```
#39
!anvi-import-misc-data Colwellia_DE_for_anvio.txt -p SPLIT_PANs/T_signals/PAN.db --target-data-table items
 --just-do-it
```

```
                                                                              [0m
* Colwellia_DE_for_anvio.txt successfully loaded


New data for 'items' in data group 'default'
===============================================
Data key "DE_d!A" ............................: Predicted type: stackedbar
Data key "DE_d!B" ............................: Predicted type: stackedbar
Data key "DE_o!A" ............................: Predicted type: stackedbar
Data key "DE_o!B" ............................: Predicted type: stackedbar
Data key "DE_od!A" ...........................: Predicted type: stackedbar
Data key "DE_od!B" ...........................: Predicted type: stackedbar
Data key "DE_odn_A" ..........................: Predicted type: int
Data key "DE_odn_B" ..........................: Predicted type: int

                                                                      209m ETA: None
NEW DATA
===============================================
Database .....................................: pan
Data group ...................................: default
Data table ...................................: items
New data keys ................................: DE_d!A, DE_d!B, DE_o!A, DE_o!B,
                                                DE_od!A, DE_od!B, DE_odn_A,
                                                DE_odn_B.
```

In [31]:

```
#40
#This cells is not compatible with Marinobacter. It loads another version of the json state so it bypass an
error of a stackedbar of no downregulated genes
!anvi-import-state -p SPLIT_PANs/T_signals/PAN.db -s pan-state_v2.json -n v2
```

```
Done .........................................: State "v2" is added to the
                                                database
```

This is a good time to check with

```
anvi-display-pan -p SPLIT_PANs/T_signals/PAN.db -g COLWELLIA_GENOMES.db
```

If everything looks right, it is adviced to go ahead and select the core by:

- Create a new bin called "Core"
- Go to search and choose "Search gene clusters using filters"
- Set "min number of genomes gene cluster occurs" to 78
- Append selection to bin

In [32]:

```
Image(filename='Figure_2.png')
```

Out[32]:

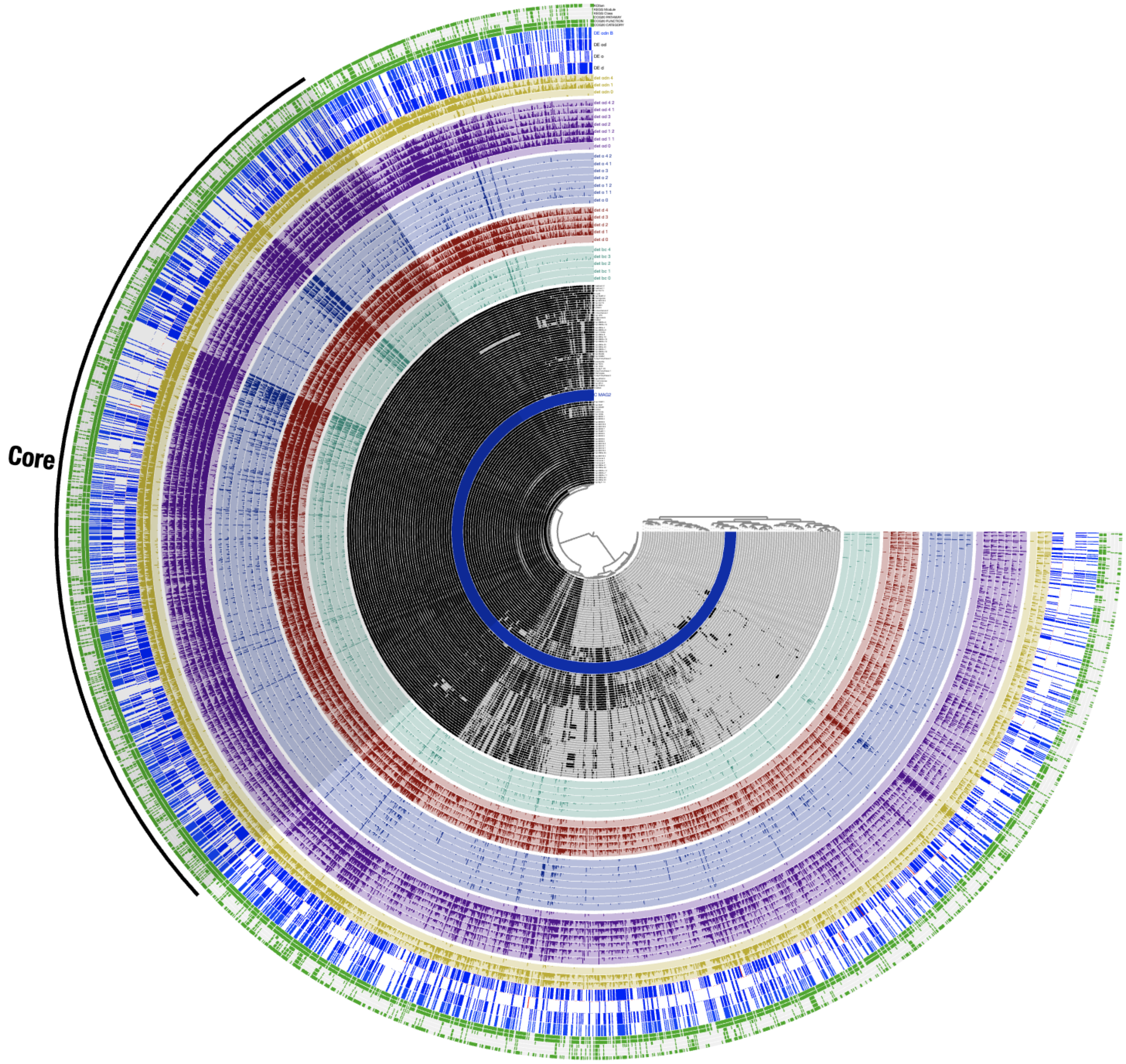


## Barplot of up down DE genes

The following barplot will be added to the anvio plot. Anvio can add a layer to samples but not to the DE layer. So we do it here and later we add in inkscape.

In [2]:
```
#41
DE_for_anvio_df = pd.read_csv(r'/Users/tito_miniconda/JOYE_LAB_ANVIO_PROJECTS/SK_BACKUP/p28_pangenomes/
Colwellia/Colwellia_DE_for_anvio.txt', sep = '\\t')
col_vec = DE_for_anvio_df.columns
col_vec = col_vec[1:9]
d = col_vec.str.split('!').tolist()
barplot_data_df = pd.DataFrame(d)
barplot_data_df = barplot_data_df.rename(columns={0: "Treatment", 1: "DE_type"})
n = barplot_data_df.shape[0]
d = np.zeros(n)
barplot_data_df['DE_total'] = d.tolist()
for index,row in DE_for_anvio_df.iterrows():
    barplot_data_df['DE_total'] = row[1:9].tolist() + barplot_data_df.DE_total
```

In [3]:
```
barplot_data_df.iloc[6,1] = "A"
barplot_data_df.iloc[7,1] = "B"
barplot_data_df.iloc[6,0] = "DE_odn"
barplot_data_df.iloc[7,0] = "DE_odn"
```

In [4]:

```
barplot_data_df
```

Out[4]:

| | Treatment | DE_type | DE_total |
|---|---|---|---|
| 0 | DE_d | A | 4.0 |
| 1 | DE_d | B | 1457.0 |
| 2 | DE_o | A | 4.0 |
| 3 | DE_o | B | 634.0 |
| 4 | DE_od | A | 3.0 |
| 5 | DE_od | B | 1137.0 |
| 6 | DE_odn | A | 0.0 |
| 7 | DE_odn | B | 1225.0 |

In [5]:

```
#42
barplot_data_df = barplot_data_df.replace(to_replace=r'^A$', value='B_DownReg', regex=True)
barplot_data_df = barplot_data_df.replace(to_replace=r'^B$', value='A_UpReg', regex=True)

barplot_4_anvio = alt.Chart(barplot_data_df).mark_bar().encode(
    x='Treatment',
    y='DE_total',
    color=alt.Color('DE_type', scale=alt.Scale(domain=['A_UpReg', 'B_DownReg'], range=['blue', 'red']))
).properties(
    width=100,
    height=100
)
```

In [6]:

```
#43
barplot_4_anvio.save('barplot_4_anvio.svg')
```

After spending some time in Inkscape this is the current stage of the figure:

In [3]:

```
Image(filename='Figure_3.png')
```

Out[3]:

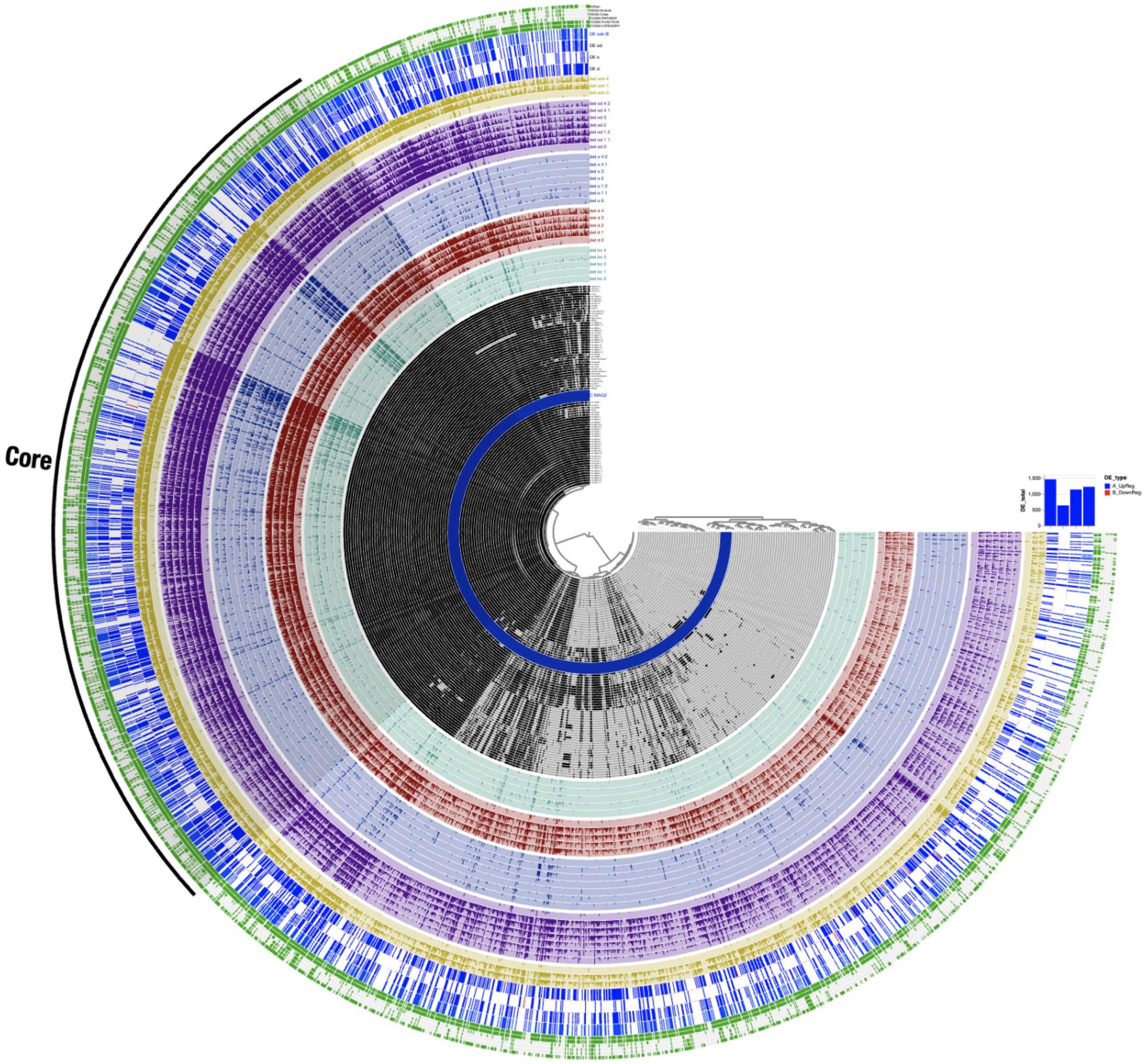


## DE genes functional (Supplementary) annotation table

In [25]:

```
#44
!anvi-export-functions -c 02_CONTIGS/C_MAG2-contigs.db -o C_MAG2-contigs_annotations.txt
```

```
Annotation sources ...........................: KOfam, Transfer_RNAs,
                                                KEGG_Class, COG20_FUNCTION,
                                                COG20_PATHWAY, KEGG_Module,
                                                COG20_CATEGORY.
Number of functions reported .................: 9,046     [0m
Output file ..................................: C_MAG2-contigs_annotations.txt
```

In [2]:

```
#45
ANNOT_df = pd.read_csv(r'/Users/tito_miniconda/JOYE_LAB_ANVIO_PROJECTS/SK_BACKUP/p28_pangenomes/Colwellia/
C_MAG2-contigs_annotations.txt', sep = '\\t')
DESEQ_df = pd.read_csv(r'/Users/tito_miniconda/JOYE_LAB_ANVIO_PROJECTS/SK_BACKUP/p28_pangenomes/
Colwellia/Colwellia_DE_df.txt', sep='\\t')
sig_df = DESEQ_df[DESEQ_df.padj < 0.05]
sig_df.rename(columns={'Gene':'gene_callers_id'}, inplace=True)
temp_df = pd.merge(sig_df,ANNOT_df,on='gene_callers_id',how='left')
temp_df.to_csv('DIFFEX_ANNOT.csv')
#From DIFFEX_ANNOT.csv we are generating a supplementary table for the paper.
temp_df["new_id"] =  temp_df["gene_callers_id"].astype(str) + temp_df["treatment"]
/Users/tito_miniconda/opt/miniconda3/envs/anvio-7/lib/python3.6/site-packages/pandas/core/frame.py:4223: SettingW
ithCopyWarning:
A value is trying to be set on a copy of a slice from a DataFrame
```

```
See the caveats in the documentation: http://pandas.pydata.org/pandas-docs/stable/user_guide/indexing.html#return
ing-a-view-versus-a-copy
  return super().rename(**kwargs)
```

In [3]:

```python
#46

## INICIALIZACION ##

ids = np.unique(temp_df.new_id.values)

i = ids[0]

test = temp_df[temp_df.new_id == i]

test2 = test.loc[:, "gene_callers_id":"abs_diff_mean"]

test2 = test2.drop_duplicates()

test3 = copy(test2)

test3 = test3.applymap(lambda x: None)

test3.columns = ["COG20_CATEGORY", "COG20_CATEGORY_description", "COG20_CATEGORY_e_value",
                 "COG20_FUNCTION", "COG20_FUNCTION_description", "COG20_FUNCTION_e_value",
                 "COG20_PATHWAY", "COG20_PATHWAY_description", "COG20_PATHWAY_e_value"]

test4 = copy(test3)

test4.columns = ["KOfam", "KOfam_description", "KOfam_e_value",
                 "KEGG_Module", "KEGG_Module_description", "KEGG_Module_e_value",
                 "KEGG_Class", "KEGG_Class_description", "KEGG_Class_e_value"]

test5 = pd.concat([test2, test4, test3], axis=1)

diffex_annot_df = test5.drop(test5.index[range(1)])


## LOOP ##

for i in ids:
    test = temp_df[temp_df.new_id == i]
    test2 = test.loc[:, "gene_callers_id":"abs_diff_mean"]
    test2 = test2.drop_duplicates()
    test3 = copy(test2)

    test3 = test3.applymap(lambda x: None)

    test3.columns = ["COG20_CATEGORY", "COG20_CATEGORY_description", "COG20_CATEGORY_e_value",
                     "COG20_FUNCTION", "COG20_FUNCTION_description", "COG20_FUNCTION_e_value",
                     "COG20_PATHWAY", "COG20_PATHWAY_description", "COG20_PATHWAY_e_value"]

    test4 = copy(test3)

    test4.columns = ["KOfam", "KOfam_description", "KOfam_e_value",
                     "KEGG_Module", "KEGG_Module_description", "KEGG_Module_e_value",
                     "KEGG_Class", "KEGG_Class_description", "KEGG_Class_e_value"]

    test5 = pd.concat([test2, test4, test3], axis=1)
    if test5.shape[0] > 1:
        print(test5)

    try:
        test5.iloc[0,9] = test[test.source == 'KOfam'].accession.values[0]
    except IndexError:
        test5.iloc[0,9] = None

    try:
        test5.iloc[0,10] = test[test.source == 'KOfam'].function.values[0]
    except IndexError:
        test5.iloc[0,10] = None

    try:
        test5.iloc[0,11] = test[test.source == 'KOfam'].e_value.values[0]
    except IndexError:
        test5.iloc[0,11] = None

    try:
        test5.iloc[0,12] = test[test.source == 'KEGG_Module'].accession.values[0]
    except IndexError:
        test5.iloc[0,12] = None
```

```
    try:
        test5.iloc[0,13] = test[test.source == 'KEGG_Module'].function.values[0]
    except IndexError:
        test5.iloc[0,13] = None

    try:
        test5.iloc[0,14] = test[test.source == 'KEGG_Module'].e_value.values[0]
    except IndexError:
        test5.iloc[0,14] = None

    try:
        test5.iloc[0,15] = test[test.source == 'KEGG_Class'].accession.values[0]
    except IndexError:
        test5.iloc[0,15] = None

    try:
        test5.iloc[0,16] = test[test.source == 'KEGG_Class'].function.values[0]
    except IndexError:
        test5.iloc[0,16] = None

    try:
        test5.iloc[0,17] = test[test.source == 'KEGG_Class'].e_value.values[0]
    except IndexError:
        test5.iloc[0,17] = None

    try:
        test5.iloc[0,18] = test[test.source == 'COG20_CATEGORY'].accession.values[0]
    except IndexError:
        test5.iloc[0,18] = None

    try:
        test5.iloc[0,19] = test[test.source == 'COG20_CATEGORY'].function.values[0]
    except IndexError:
        test5.iloc[0,19] = None

    try:
        test5.iloc[0,20] = test[test.source == 'COG20_CATEGORY'].e_value.values[0]
    except IndexError:
        test5.iloc[0,20] = None

    try:
        test5.iloc[0,21] = test[test.source == 'COG20_FUNCTION'].accession.values[0]
    except IndexError:
        test5.iloc[0,21] = None

    try:
        test5.iloc[0,22] = test[test.source == 'COG20_FUNCTION'].function.values[0]
    except IndexError:
        test5.iloc[0,22] = None

    try:
        test5.iloc[0,23] = test[test.source == 'COG20_FUNCTION'].e_value.values[0]
    except IndexError:
        test5.iloc[0,23] = None

    try:
        test5.iloc[0,24] = test[test.source == 'COG20_PATHWAY'].accession.values[0]
    except IndexError:
        test5.iloc[0,24] = None

    try:
        test5.iloc[0,25] = test[test.source == 'COG20_PATHWAY'].function.values[0]
    except IndexError:
        test5.iloc[0,25] = None

    try:
        test5.iloc[0,26] = test[test.source == 'COG20_PATHWAY'].e_value.values[0]
    except IndexError:
        test5.iloc[0,26] = None


    frames = [diffex_annot_df, test5]

    diffex_annot_df = pd.concat(frames)

diffex_annot_df.to_csv('DIFFEX_ANNOT_v2.csv')
```

## Selecting and splitting only GC containing DE genes.

Anvio diagram is getting too difficult to label names. So we are going to select and split bins that only contain DE genes. Selection was done using the search box and now we proceed to split again.

In [4]:

```
#47
!anvi-split -p SPLIT_PANs/T_signals/PAN.db -g COLWELLIA_GENOMES.db -C DE_Genes -o SPLIT_DE_Genes
```

```
Genomes storage ...............................................: Initialized[48;5;239m ETA: 0s
                                                                 (storage hash:
                                                                 hashdd453a5a)
Num genomes in storage ........................................: 78
Num genomes will be used ......................................: 78
Pan DB ........................................................: Initialized:
                                                                 SPLIT_PANs/T_signals/PAN.db
                                                                 (v. 14)
Gene cluster homogeneity estimates ............................: Functional:
                                                                 [YES]; Geometric:
                                                                 [YES]; Combined:
                                                                 [YES]
                                                                                 [0m
* Gene clusters are initialized for all 2731 gene clusters in the database.

                                                                          [0m
WARNING
==============================================================
Anvi'o is about to start splitting your bins into individual, self-contained
anvi'o profiles. This is quite a tricky operation, and even if it finishes
successfully, you must double check everyting in the resulting profiles to make
sure things worked as expected. Although we are doing our best to test all
these, variation between projects make it impossible to be 100% sure.

Collections ...............................: The collection 'DEFAULT' that
                                              describes 1598 splits in a
                                              single bin was successfully
                                              added to the to the database at
                                              'SPLIT_DE_Genes/DE_Genes/PAN.db'.
                                              Here is a full list of the bin
                                              names in this collection:
                                              ALL_SPLITS.
New items order ...........................: "frequency:euclidean:ward" (type
                                              newick) has been added to the
                                              database...
                                                                          [0m
WARNING
===============================================
Clustering for "frequency:euclidean:ward" is already in the database. It will be
replaced with the new content.

New items order ...........................: "frequency:euclidean:ward" (type
                                              newick) has been added to the
                                              database...
New items order ...........................: "presence-absence:euclidean:ward"
                                              (type newick) has been added to
                                              the database...
Num bins processed ............................................: 1
Output directory ..............................................: /Users/tito_miniconda/JOYE_LAB_ANVIO_PROJECTS/SK_B
ACKUP/p28_pangenomes/Colwellia/SPLIT_DE_Genes
```

```
anvi-display-pan -p SPLIT_DE_Genes/DE_genes/PAN.db -g COLWELLIA_GENOMES.db
```

## Static HTML Summary

Now our CA-metatranscriptome is ready to generate an static HTML output.

In [2]:

```
#47
!anvi-summarize -p SPLIT_DE_Genes/DE_genes/PAN.db -g COLWELLIA_GENOMES.db -o SPLIT_DE_signals-SUMMARY -C
core_v2
```

```
Genomes storage ...............................................: Initialized[48;5;239m ETA: 0s
                                                                 (storage hash:
                                                                 hashdd453a5a)
Num genomes in storage ........................................: 78
Num genomes will be used ......................................: 78
Pan DB ........................................................: Initialized:
                                                                 SPLIT_DE_Genes/DE_genes/PAN.db
                                                                 (v. 14)
Gene cluster homogeneity estimates ............................: Functional:
                                                                 [YES]; Geometric:
                                                                 [YES]; Combined:
                                                                 [YES]
                                                                                 [0m
* Gene clusters are initialized for all 1598 gene clusters in the database.
```

```
                                                                       [0m[38;5;0m ETA: 0s
Misc data reported for layers ................................: default
                                                                       [0m
Misc data reported for items ................................: default
                                                                       [0m
HTML Output ..................................................: /Users/tito_miniconda/JOYE_LAB_ANVIO_PROJECTS/SK_B
ACKUP/p28_pangenomes/Colwellia/SPLIT_DE_signals-SUMMARY/index.html
```
