# Supplementary material for: Species-specific responses of marine bacteria to environmental perturbation
Source: ISME Commun. 2023 Sep 22;3:99. doi: 10.1038/s43705-023-00310-z (PMC10516948; doi:10.1038/s43705-023-00310-z)
Supplement: Supplementary file 3 — Data Set 4 [file 43705_2023_310_MOESM3_ESM.pdf]

In [1]:

```
#1
#import sys
#!{sys.executable} -m pip install altair vega_datasets
```

In [1]:

```
#2
import altair as alt
from IPython.display import Image
import numpy as np
import os
import pandas as pd
import re
from scipy import stats
from copy import copy
```

In [3]:

```
#3
def isNaN(num):
    return num != num

def unique(list1):
    x = np.array(list1)
    print(np.unique(x))
```

Marinobacter CA-Metatranscriptome

First we followed http://merenlab.org/2019/03/14/ncbi-genome-download-magic/

In [4]:

```
#4
!ncbi-genome-download bacteria -l 'all' -g 'Marinobacter' -m metadata.txt
#Careful that Marinobacterium gets downloaded here too.
```

Process metadata.txt to download NCBI genomes

In [5]:

```
#5
!anvi-script-process-genbank-metadata -m metadata.txt --output-dir NCBI_genomes
```

```
Input metadata file ...........................: metadata.txt
Output directory ..............................: /Users/tito_miniconda/JOYE_LAB_A
NVIO_PROJECTS/SK_BACKUP/p28_pangenomes/Marinobacter/NCBI_genomes
Num entries in metadata .......................: 152
Output FASTA descriptor .......................: Marinobacter.txt;5;0m ETA: 0s
```

In [6]:

```
#6
!anvi-run-workflow -w contigs --get-default-config default_config.txt
```

```
WARNING
===============================================
If you publish results from this workflow, please do not forget to cite
snakemake (doi:10.1093/bioinformatics/bts480)
```

```
WARNING
===============================================
We are initiating parameters for the contigs workflow

Default config file ...........................: Stored for workflow 'contigs' as
                                                  'default_config.txt'.
```

Before running the next line, make sure you add in the `fasta.txt` file those genomes that were not automatically downloaded in the previous line (e.g. SAGs, MAGs, or unpublished genomes).

Here we include 15 **Marinobacter MAGs** from Parks et al 2017 and 2 unpublished Marinobacter genomes *Marinobacter TT1* and *Marinobacter TK36*

Here it is needed to run the next lines to reduce probability of errors from Anvio

In [11]:

```
#7

!mkdir GENOMES_OK

#!ls NCBI_genomes | grep fa | awk '{ print "awk %{ if ($0 ~ /^>/) print $0;
else {gsub(/[RKWYMSVB]/, %%N%% , $0); print $0 }}% NCBI_genomes/\" $0 \" > GENOMES_OK/\" $0}'
!bash fix1.sh

#!mv fasta.txt fasta.txt_v0

!echo -e "name\tpath" > fasta.txt

!ls GENOMES_OK | sed -e 's/\.fa//g' | awk '{print $0 "\t/Users/tito_miniconda/
JOYE_LAB_ANVIO_PROJECTS/SK_BACKUP/p28_pangenomes/Marinobacter/GENOMES_OK/\" $0 \".fa\"}' >> fasta.txt
```

Run this, if anvio is newly installed.

```
anvi-setup-scg-taxonomy
```

and

```
anvi-setup-ncbi-cogs
```

Then, the following line was run.

```
anvi-run-workflow -w contigs -c default_config.txt
```

Now creating `external_genomes.txt` file

In [12]:

```
#8
!rm external_genomes.txt
!echo -e 'name\tcontigs_db_path' > external_genomes.txt
!ls ./02_CONTIGS/ | grep '.db' | sed -e "s/-contigs.db//" | awk '{print $0"\t/Users/tito_miniconda/
JOYE_LAB_ANVIO_PROJECTS/SK_BACKUP/p28_pangenomes/Marinobacter/02_CONTIGS/\"$0\"-contigs.db\"}' >>
external_genomes.txt\n"
```

```
rm: external_genomes.txt: No such file or directory
```

Now creating the database of Marinobacter genomes.

In [13]:

```
#9
```

```
!anvi-gen-genomes-storage -e external_genomes.txt -o MARINOBACTER_GENOMES.db
```

```
                                                                        ETA: N
one
WARNING
===============================================
Good news! Anvi'o found all these functions that are common to all of your
genomes and will use them for downstream analyses and is very proud of you:
'KEGG_Class, KEGG_Module, COG20_PATHWAY, KOfam, Transfer_RNAs, COG20_CATEGORY,
COG20_FUNCTION'.

Internal genomes ............................: 0 have been initialized.
External genomes ............................: 180 found.9m ETA: 0s
                                                                      [0m
JUST FYI
===============================================
Some of your genomes had gene calls identified by gene callers other than the
anvi'o default, 'prodigal', and will not be processed. Use the `--debug` flag if
this sounds important and you would like to see more of this message.


 [0m
* M_adhaerens_1 is stored with 3,896 genes (15 of which were partial)
* M_adhaerens_2 is stored with 3,153 genes (21 of which were partial)    [0m
* M_adhaerens_3 is stored with 4,093 genes (2 of which were partial)     [0m
* M_adhaerens_4 is stored with 3,749 genes (8 of which were partial)     [0m
* M_adhaerens_5 is stored with 4,388 genes (266 of which were partial)   [0m
* M_adhaerens_6 is stored with 4,353 genes (4 of which were partial)     [0m
* M_alexandrii is stored with 3,979 genes (16 of which were partial)     [0m
* M_algicola is stored with 4,106 genes (72 of which were partial)       [0m
* M_antarcticus is stored with 3,440 genes (10 of which were partial)    [0m
* M_aromaticivorans is stored with 3,778 genes (36 of which were partial)[0m
* M_bohaiensis is stored with 4,291 genes (7 of which were partial)      [0m
* M_caseinilyticus is stored with 3,847 genes (34 of which were partial) [0m
* M_changyiensis is stored with 3,977 genes (14 of which were partial)   [0m
* M_confluentis_1 is stored with 3,534 genes (110 of which were partial) [0m
* M_confluentis_2 is stored with 3,432 genes (16 of which were partial)  [0m
* M_daepoensis_1 is stored with 3,515 genes (18 of which were partial)   [0m
* M_daepoensis_2 is stored with 3,524 genes (17 of which were partial)   [0m
* M_daqiaonensis_1 is stored with 3,554 genes (0 of which were partial)  [0m
* M_daqiaonensis_2 is stored with 3,558 genes (6 of which were partial)  [0m
* M_excellens_1 is stored with 3,665 genes (1 of which were partial)     [0m
* M_excellens_2 is stored with 4,046 genes (44 of which were partial)    [0m
* M_flavimaris_1 is stored with 4,148 genes (51 of which were partial)   [0m
* M_flavimaris_2 is stored with 4,120 genes (25 of which were partial)   [0m
* M_fonticola is stored with 4,005 genes (1 of which were partial)       [0m
* M_fuscus is stored with 3,868 genes (95 of which were partial)         [0m
* M_gelidimuriae is stored with 3,684 genes (166 of which were partial)  [0m
* M_gudaonensis is stored with 3,459 genes (4 of which were partial)     [0m
* M_guineae is stored with 4,153 genes (20 of which were partial)        [0m
* M_halodurans is stored with 4,989 genes (287 of which were partial)    [0m
* M_halophilus_1 is stored with 3,564 genes (10 of which were partial)   [0m
* M_halophilus_2 is stored with 3,546 genes (3 of which were partial)    [0m
* M_halotolerans is stored with 3,471 genes (1 of which were partial)    [0m
* M_hydrocarbonoclasticus_01 is stored with 3,655 genes (1 of which were partia
l)
* M_hydrocarbonoclasticus_02 is stored with 3,929 genes (13 of which were partia
l)
* M_hydrocarbonoclasticus_03 is stored with 4,007 genes (22 of which were partia
l)
* M_hydrocarbonoclasticus_04 is stored with 4,100 genes (42 of which were partia
l)
* M_hydrocarbonoclasticus_05 is stored with 4,005 genes (21 of which were partia
l)
* M_hydrocarbonoclasticus_06 is stored with 3,697 genes (6 of which were partia
```

```
l)
* M_hydrocarbonoclasticus_07 is stored with 3,679 genes (14 of which were partia
l)
* M_hydrocarbonoclasticus_08 is stored with 3,850 genes (42 of which were partia
l)
* M_hydrocarbonoclasticus_09 is stored with 3,953 genes (29 of which were partia
l)
* M_hydrocarbonoclasticus_10 is stored with 3,583 genes (32 of which were partia
l)
* M_hydrocarbonoclasticus_11 is stored with 4,453 genes (0 of which were partia
l)
* M_lipolyticus_1 is stored with 3,684 genes (166 of which were partial) [0m
* M_lipolyticus_2 is stored with 3,462 genes (50 of which were partial)  [0m
* M_lipolyticus_3 is stored with 3,641 genes (14 of which were partial)  [0m
* M_litoralis_1 is stored with 3,148 genes (3 of which were partial)     [0m
* M_litoralis_2 is stored with 3,201 genes (9 of which were partial)     [0m
* M_lutaoensis_1 is stored with 3,507 genes (27 of which were partial)   [0m
* M_lutaoensis_2 is stored with 3,268 genes (79 of which were partial)   [0m
* M_manganoxydans is stored with 4,219 genes (92 of which were partial)  [0m
* M_maritimus is stored with 3,974 genes (8 of which were partial)       [0m
* M_maroccanus is stored with 3,997 genes (56 of which were partial)     [0m
* M_mobilis is stored with 3,614 genes (22 of which were partial)        [0m
* M_nanhaiticus is stored with 4,834 genes (6 of which were partial)     [0m
* M_nitratireducens is stored with 3,512 genes (16 of which were partial)[0m
* M_orientalis_1 is stored with 3,612 genes (10 of which were partial)   [0m
* M_orientalis_2 is stored with 3,643 genes (11 of which were partial)   [0m
* M_oulmenensis is stored with 3,404 genes (14 of which were partial)    [0m
* M_pelagius_1 is stored with 3,830 genes (24 of which were partial)     [0m
* M_pelagius_2 is stored with 3,515 genes (15 of which were partial)     [0m
* M_persicus_1 is stored with 3,313 genes (68 of which were partial)     [0m
* M_persicus_2 is stored with 3,312 genes (68 of which were partial)     [0m
* M_persicus_3 is stored with 3,319 genes (75 of which were partial)     [0m
* M_persicus_4 is stored with 2,944 genes (22 of which were partial)     [0m
* M_persicus_5 is stored with 2,945 genes (21 of which were partial)     [0m
* M_piscensis is stored with 3,022 genes (6 of which were partial)       [0m
* M_profundi is stored with 3,632 genes (36 of which were partial)       [0m
* M_psychrophilus is stored with 3,597 genes (0 of which were partial)   [0m
* M_salarius_1 is stored with 5,369 genes (1 of which were partial)      [0m
* M_salarius_2 is stored with 4,272 genes (3 of which were partial)      [0m
* M_salarius_3 is stored with 4,168 genes (2 of which were partial)      [0m
* M_salarius_4 is stored with 4,222 genes (135 of which were partial)    [0m
* M_salarius_5 is stored with 4,070 genes (214 of which were partial)    [0m
* M_salarius_6 is stored with 4,149 genes (64 of which were partial)     [0m
* M_salarius_7 is stored with 4,154 genes (57 of which were partial)     [0m
* M_salexigens_1 is stored with 3,460 genes (24 of which were partial)   [0m
* M_salexigens_2 is stored with 3,736 genes (13 of which were partial)   [0m
* M_salicampi is stored with 3,991 genes (17 of which were partial)      [0m
* M_salinus is stored with 3,780 genes (2 of which were partial)         [0m
* M_salsuginis_1 is stored with 3,782 genes (6 of which were partial)    [0m
* M_salsuginis_2 is stored with 4,349 genes (78 of which were partial)   [0m
* M_salsuginis_3 is stored with 4,008 genes (1 of which were partial)    [0m
* M_salsuginis_4 is stored with 8,035 genes (1,052 of which were partial)[0m
* M_santoriniensis is stored with 3,729 genes (34 of which were partial) [0m
* M_sediminum is stored with 3,375 genes (11 of which were partial)      [0m
* M_segnicrescens is stored with 3,934 genes (110 of which were partial) [0m
* M_shengliensis_1 is stored with 3,780 genes (30 of which were partial) [0m
* M_shengliensis_2 is stored with 4,356 genes (16 of which were partial) [0m
* M_similis is stored with 4,590 genes (0 of which were partial)         [0m
* M_sp_1_3A is stored with 3,712 genes (29 of which were partial)        [0m
* M_sp_1_4A is stored with 3,682 genes (18 of which were partial)        [0m
* M_sp_3_2 is stored with 4,084 genes (8 of which were partial)          [0m
* M_sp_AC is stored with 4,440 genes (125 of which were partial)         [0m
* M_sp_ANT is stored with 3,822 genes (8 of which were partial)          [0m
* M_sp_Arc7 is stored with 3,958 genes (2 of which were partial)         [0m
* M_sp_BSs20148 is stored with 3,694 genes (1 of which were partial)     [0m
```

```
* M_sp_BW6 is stored with 3,914 genes (104 of which were partial)       [0m
* M_sp_C18 is stored with 4,591 genes (20 of which were partial)        [0m
* M_sp_C1S70 is stored with 3,848 genes (80 of which were partial)      [0m
* M_sp_CAU is stored with 3,761 genes (5 of which were partial)         [0m
* M_sp_CHFG3 is stored with 4,243 genes (15 of which were partial)      [0m
* M_sp_CLL7 is stored with 3,977 genes (14 of which were partial)       [0m
* M_sp_CP1 is stored with 4,395 genes (2 of which were partial)         [0m
* M_sp_DS40M8 is stored with 4,610 genes (1,224 of which were partial)  [0m
* M_sp_DSM_11874 is stored with 3,678 genes (11 of which were partial)  [0m
* M_sp_DSM_26291 is stored with 4,154 genes (57 of which were partial)  [0m
* M_sp_DSM_26671 is stored with 4,458 genes (107 of which were partial) [0m
* M_sp_DY40 is stored with 3,515 genes (16 of which were partial)       [0m
* M_sp_EC is stored with 4,022 genes (5 of which were partial)          [0m
* M_sp_ELB17 is stored with 4,653 genes (90 of which were partial)      [0m
* M_sp_EN3 is stored with 3,722 genes (79 of which were partial)        [0m
* M_sp_ES is stored with 3,360 genes (85 of which were partial)         [0m
* M_sp_EVN1 is stored with 4,017 genes (67 of which were partial)       [0m
* M_sp_EhC06 is stored with 4,235 genes (23 of which were partial)      [0m
* M_sp_EhN04 is stored with 4,236 genes (26 of which were partial)      [0m
* M_sp_F3R08 is stored with 3,913 genes (34 of which were partial)      [0m
* M_sp_F3R11 is stored with 3,051 genes (20 of which were partial)      [0m
* M_sp_F4206 is stored with 3,695 genes (6 of which were partial)       [0m
* M_sp_F4218 is stored with 3,779 genes (6 of which were partial)       [0m
* M_sp_HL_58 is stored with 3,895 genes (2 of which were partial)       [0m
* M_sp_JB02H27 is stored with 4,519 genes (30 of which were partial)    [0m
* M_sp_JB05H06 is stored with 4,520 genes (29 of which were partial)    [0m
* M_sp_JH2 is stored with 3,340 genes (4 of which were partial)         [0m
* M_sp_JSM is stored with 4,314 genes (9 of which were partial)         [0m
* M_sp_LPB0319 is stored with 3,358 genes (1 of which were partial)     [0m
* M_sp_LQ44 is stored with 4,066 genes (0 of which were partial)        [0m
* M_sp_LV10MA510 is stored with 4,245 genes (1 of which were partial)   [0m
* M_sp_LV10R510_11A is stored with 4,279 genes (1 of which were partial) [0m
* M_sp_LV10R510_8 is stored with 4,244 genes (0 of which were partial)  [0m
* M_sp_LV10R520 is stored with 4,187 genes (1 of which were partial)    [0m
* M_sp_LZ_6 is stored with 4,356 genes (16 of which were partial)       [0m
* M_sp_LZ_8 is stored with 3,979 genes (16 of which were partial)       [0m
* M_sp_MC3 is stored with 4,436 genes (66 of which were partial)        [0m
* M_sp_MCTG268 is stored with 4,098 genes (21 of which were partial)    [0m
* M_sp_MW3 is stored with 4,436 genes (67 of which were partial)        [0m
* M_sp_N1 is stored with 3,971 genes (1 of which were partial)          [0m
* M_sp_N4 is stored with 3,997 genes (56 of which were partial)         [0m
* M_sp_NP_4 is stored with 4,240 genes (5 of which were partial)        [0m
* M_sp_NP_6 is stored with 4,095 genes (6 of which were partial)        [0m
* M_sp_P4B1 is stored with 3,452 genes (2 of which were partial)        [0m
* M_sp_PJ_16 is stored with 4,021 genes (2 of which were partial)       [0m
* M_sp_PT19DW is stored with 4,085 genes (9 of which were partial)      [0m
* M_sp_R17 is stored with 4,184 genes (41 of which were partial)        [0m
* M_sp_THAF197a is stored with 3,908 genes (1 of which were partial)    [0m
* M_sp_THAF39 is stored with 3,965 genes (1 of which were partial)      [0m
* M_sp_TK36 is stored with 4,458 genes (107 of which were partial)      [0m
* M_sp_TT1 is stored with 4,154 genes (57 of which were partial)        [0m
* M_sp_UBA1922 is stored with 3,923 genes (681 of which were partial)   [0m
* M_sp_UBA2498 is stored with 4,269 genes (240 of which were partial)   [0m
* M_sp_UBA2504 is stored with 4,502 genes (219 of which were partial)   [0m
* M_sp_UBA2678 is stored with 4,949 genes (327 of which were partial)   [0m
* M_sp_UBA2688 is stored with 3,951 genes (26 of which were partial)    [0m
* M_sp_UBA2698 is stored with 3,802 genes (27 of which were partial)    [0m
* M_sp_UBA3182 is stored with 3,400 genes (107 of which were partial)   [0m
* M_sp_UBA3604 is stored with 3,824 genes (33 of which were partial)    [0m
* M_sp_UBA3607 is stored with 2,826 genes (17 of which were partial)    [0m
* M_sp_UBA4153 is stored with 3,626 genes (575 of which were partial)   [0m
* M_sp_UBA5687 is stored with 3,508 genes (239 of which were partial)   [0m
* M_sp_UBA6499 is stored with 2,873 genes (129 of which were partial)   [0m
* M_sp_UBA6604 is stored with 3,239 genes (660 of which were partial)   [0m
* M_sp_UBA7807 is stored with 2,990 genes (147 of which were partial)   [0m
```

```
* M_sp_UBA856 is stored with 2,843 genes (88 of which were partial)      [0m
* M_sp_W62 is stored with 3,612 genes (10 of which were partial)         [0m
* M_sp_X15 is stored with 3,345 genes (1 of which were partial)          [0m
* M_sp_YJ is stored with 4,989 genes (287 of which were partial)         [0m
* M_sp_YWL01 is stored with 5,183 genes (1,051 of which were partial)    [0m
* M_sp_ZYF650 is stored with 3,920 genes (145 of which were partial)     [0m
* M_sp_bablab_1 is stored with 3,408 genes (144 of which were partial)   [0m
* M_sp_bablab_2 is stored with 3,866 genes (85 of which were partial)    [0m
* M_sp_bablab_3 is stored with 3,416 genes (149 of which were partial)   [0m
* M_sp_es_042 is stored with 3,571 genes (0 of which were partial)       [0m
* M_sp_es_048 is stored with 3,754 genes (3 of which were partial)       [0m
* M_sp_lvr2a5a20 is stored with 4,529 genes (2 of which were partial)    [0m
* M_subterrani is stored with 4,193 genes (3 of which were partial)      [0m
* M_vinifirmus is stored with 3,579 genes (54 of which were partial)     [0m
* M_vulgaris_1 is stored with 3,560 genes (61 of which were partial)     [0m
* M_vulgaris_2 is stored with 3,514 genes (20 of which were partial)     [0m
* M_zhanjiangensis is stored with 3,726 genes (10 of which were partial) [0m
* M_zhejiangensis is stored with 3,668 genes (14 of which were partial)  [0m

The new genomes storage ......................: MARINOBACTER_GENOMES.db (v7,
                                                signature: hash220097ad)
Number of genomes ............................: 180 (internal: 0, external: 180)
Number of gene calls .........................: 701,633
Number of partial gene calls .................: 12,783
```

Now we can run the pangenomic analysis

```
anvi-pan-genome -g MARINOBACTER_GENOMES.db --project-name "Marinobacter"
--output-dir MARINOBACTER --num-threads 6 --minbit 0.5 --mcl-inflation 10
--use-ncbi-blast --enforce-hierarchical-clustering
```

To display the pangenome.

```
anvi-display-pan -p MARINOBACTER/Marinobacter-PAN.db -g
MARINOBACTER_GENOMES.db
```

Selection of a genomic reference

The anvio pangenome analysis doesn't offer to generate a *consensus* reference from a set of genomes. Therefore, if we have 27 metatranscriptomic libraries and 180 genomes in the pangenome, this translates into 27 x 180 = 4860 mapping procedures (and ultimately 4860 BAM files), which is not feasible to plot or to process in a single figure. Therefore we need to chose only one genome as mapping reference for the metapangenomic analysis. We are going to choose the genome that recruits the largest amount of reads from our transcriptomic libraries.

Anvio requires that contig names are the same in the contig database as well as BAM files. To prevent future errors, we are going to export fasta files from the contig databases and they are going to be used for downstream processes.

In [ ]:

```
#10
#!ls 02_CONTIGS/*contigs.db | cut -d'/' -f2 | sed -e 's/-contigs.db//'| awk
'{print \"!anvi-export-contigs -c 02_CONTIGS/\" $0 \"-contigs.db -o \" $0 \"_contigs.fa\"}'
#!mkdir NCBI_genomes_v2
#!mv *.fa NCBI_genomes_v2
```

```
#Next two lines copy a list of output files from a previous matching of mapping files
#!mkdir 98_SCREENING
#!cp ../Marinobacter/98_SCREENING/* ./980_SCREENING
```

In [16]:

```
!ls 98_SCREENING/
```

```
map_OIL11_e.txt map_OIL31_e.txt map_OIL50_e.txt map_OIL70_e.txt map_OIL87_e.txt
map_OIL14_e.txt map_OIL32_e.txt map_OIL53_e.txt map_OIL78_e.txt map_OIL8_e.txt
map_OIL17_e.txt map_OIL34_e.txt map_OIL5_e.txt  map_OIL81_e.txt map_OIL90_e.txt
map_OIL25_e.txt map_OIL37_e.txt map_OIL61_e.txt map_OIL82_e.txt
map_OIL28_e.txt map_OIL44_e.txt map_OIL64_e.txt map_OIL84_e.txt
map_OIL29_e.txt map_OIL47_e.txt map_OIL67_e.txt map_OIL85_e.txt
```

In [17]:

```
#11
map_e_files = ['map_OIL11_e.txt','map_OIL32_e.txt','map_OIL53_e.txt','map_OIL81_e.txt','map_OIL14_e.txt','map_OIL34_e.txt',
'map_OIL61_e.txt','map_OIL82_e.txt','map_OIL17_e.txt','map_OIL37_e.txt','map_OIL64_e.txt','map_OIL84_e.txt','map_OIL25_e.txt',
'map_OIL44_e.txt','map_OIL67_e.txt','map_OIL85_e.txt','map_OIL28_e.txt','map_OIL47_e.txt','map_OIL70_e.txt','map_OIL87_e.txt',
'map_OIL29_e.txt','map_OIL5_e.txt','map_OIL78_e.txt','map_OIL90_e.txt', 'map_OIL31_e.txt', 'map_OIL50_e.txt', 'map_OIL8_e.txt']
row_names = ['od_0','od_1_2','d_2','o_4_1','d_0','d_1','bc_3','o_4_2','odn_0','odn_1','o_3','od_4_1','bc_1','bc_2','od_3','od_4_2',
'o_1_1','o_2','d_3','d_4','o_1_2','bc_0','bc_4','odn_4','od_1_1','od_2','o_0']
data_mapped = []
data_rates = []
totalreads_vec = []
for e_file in map_e_files:

    #file = map_e_files[0]
    root = r'/Users/tito_miniconda/JOYE_LAB_ANVIO_PROJECTS/SK_BACKUP/p28_pangenomes/Marinobacter/98_SCREENING/
    location = root + e_file
    with open(location) as f:
        lines = f.readlines()
    lines = [x.strip() for x in lines]
    j = 0

    mapped_vec = []
    rates_vec = []
    for i in lines:
        if j == 0:
            fields = i.split(' ')
            total_reads = int(fields[0]) * 2
        if j == 3:
            fields = i.split(' ')
            l1 = int(fields[0]) * 2
        if j == 4:
            fields = i.split(' ')
            l2 = int(fields[0]) * 2
        if j == 7:
            fields = i.split(' ')
            l3 = int(fields[0]) * 2
        if j == 12:
            fields = i.split(' ')
            l4 = int(fields[0])
        if j == 13:
            fields = i.split(' ')
            l5 = int(fields[0])
            mapped = l1 + l2 + l3 + l4 + l5
            mapped_vec.append(mapped)
            rate = mapped * 100 / total_reads
            rates_vec.append(rate)
        if j == 14:
            j = -1
        j = j +1
```

```
    totalreads_vec.append(total_reads)
    data_mapped.append(mapped_vec)
    data_rates.append(rates_vec)
```

In [19]:

```
#12
location_script = r'/Users/tito_miniconda/JOYE_LAB_ANVIO_PROJECTS/SK_BACKUP/p28_pangenomes/Marinobacter/99_SCRIPTS/temp.txt'
with open(location_script) as f:
    lines = f.readlines()

lines = [x.strip() for x in lines]

species = []
for i in lines:
    if re.match(r"^bowtie2", i):
        fields = i.split(' ')
        fields0 = fields[3].split('/')
        fields1 = fields0[1].split('_in')
        species.append(fields1[0])
```

In [20]:

```
#13
# Creating pandas DataFrames
mapped_df = pd.DataFrame(data_mapped, columns = species, index=row_names)
maprates_df = pd.DataFrame(data_rates, columns = species, index=row_names)
```

Based on the average of mapped reads per library:

In [21]:

```
#14
mapped_df.mean(axis = 0).sort_values(ascending=False).head(10)
```

Out[21]:

```
Marinobacter_sp__C18_GCF_001924925                      63195.703704
Marinobacter_sp__NP_6_GCF_003997005                     58878.814815
Marinobacter_salarius_GCF_900114695_1-contigs.fa        57842.444444
Marinobacter_sp__DSM_26291_GCF_900114695                57842.444444
Marinobacter_sp__TT1.fa                                 57842.407407
Marinobacter_sp__EC_HK377_GCF_902498775                 57531.370370
Marinobacter_sp__N1_GCF_902506385                       57522.296296
Marinobacter_MAG5.fa                                    56721.148148
Marinobacter_salarius_GCF_016461915_1-contigs.fa        56190.888889
Marinobacter_salarius_GCF_018424725_1-contigs.fa        55557.111111
dtype: float64
```


```
Marinobacter_sp__C18_GCF_001924925                      63195.703704
Marinobacter_sp__NP_6_GCF_003997005                     58878.814815
Marinobacter_salarius_GCF_900114695_1-contigs.fa        57842.444444
Marinobacter_sp__DSM_26291_GCF_900114695                57842.444444
Marinobacter_sp__TT1.fa                                 57842.407407
Marinobacter_sp__EC_HK377_GCF_902498775                 57531.370370
Marinobacter_sp__N1_GCF_902506385                       57522.296296
Marinobacter_MAG5.fa                                    56721.148148
Marinobacter_salarius_GCF_016461915_1-contigs.fa        56190.888889
Marinobacter_salarius_GCF_018424725_1-contigs.fa        55557.111111
dtype: float64
```


Based on the average of the greatest (maximum) number of mapped reads in a library :

In [22]:

```
#15
mapped_df.max(axis = 0).sort_values(ascending=False).head(10)
```

Out[22]:

```
Marinobacter_sp__C18_GCF_001924925                   626644
Marinobacter_sp__NP_6_GCF_003997005                  597868
Marinobacter_sp__N1_GCF_902506385                    582802
Marinobacter_sp__EC_HK377_GCF_902498775              582778
Marinobacter_salarius_GCF_016461915_1-contigs.fa     571215
Marinobacter_MAG5.fa                                 568106
Marinobacter_salarius_GCF_900114695_1-contigs.fa     565753
Marinobacter_sp__DSM_26291_GCF_900114695             565753
Marinobacter_sp__TT1.fa                              565752
Marinobacter_salarius_GCF_002116735                  556510
dtype: int64
```


```
Marinobacter_sp__C18_GCF_001924925                   626644
Marinobacter_sp__NP_6_GCF_003997005                  597868
Marinobacter_sp__N1_GCF_902506385                    582802
Marinobacter_sp__EC_HK377_GCF_902498775              582778
Marinobacter_salarius_GCF_016461915_1-contigs.fa     571215
Marinobacter_MAG5.fa                                 568106
Marinobacter_salarius_GCF_900114695_1-contigs.fa     565753
Marinobacter_sp__DSM_26291_GCF_900114695             565753
Marinobacter_sp__TT1.fa                              565752
Marinobacter_salarius_GCF_002116735                  556510
dtype: int64
```


Based on the average of mapping rates per library:

In [23]:

```
#16
maprates_df.mean(axis = 0).sort_values(ascending=False).head(10)
```

Out[23]:

```
Marinobacter_sp__C18_GCF_001924925                   0.964301
Marinobacter_sp__NP_6_GCF_003997005                  0.903556
Marinobacter_sp__EC_HK377_GCF_902498775              0.884463
Marinobacter_sp__N1_GCF_902506385                    0.884388
Marinobacter_sp__DSM_26291_GCF_900114695             0.881725
Marinobacter_salarius_GCF_900114695_1-contigs.fa     0.881725
Marinobacter_sp__TT1.fa                              0.881724
Marinobacter_MAG5.fa                                 0.867590
Marinobacter_salarius_GCF_016461915_1-contigs.fa     0.864312
Marinobacter_salarius_GCF_018424725_1-contigs.fa     0.849280
dtype: float64
```


```
Marinobacter_sp__C18_GCF_001924925                   0.964301
Marinobacter_sp__NP_6_GCF_003997005                  0.903556
Marinobacter_sp__EC_HK377_GCF_902498775              0.884463
Marinobacter_sp__N1_GCF_902506385                    0.884388
Marinobacter_sp__DSM_26291_GCF_900114695             0.881725
Marinobacter_salarius_GCF_900114695_1-contigs.fa     0.881725
Marinobacter_sp__TT1.fa                              0.881724
Marinobacter_MAG5.fa                                 0.867590
Marinobacter_salarius_GCF_016461915_1-contigs.fa     0.864312
```

```
Marinobacter_salarius_GCF_018424725_1-contigs.fa    0.849280
dtype: float64
```

The largest yield of reads recruitment is obtained using the genome of ***Marinobacter sp C18***

Now I export the dataframes to prepare an excel sheet.

In [24]:
```
#17
mapped_df.to_csv('00_Marinobacter_mapping_reads.csv')
maprates_df.to_csv('01_Marinobacter_mapping_reads_rates.csv')
```

anvi BAM profile and downstream analysis

Now that we identified the best possible genomic reference, we can proceed to map. First we need the right contigs to do the mapping.

In [25]:
```
#18
!anvi-export-contigs -c 02_CONTIGS/M_sp_C18-contigs.db -o M_sp_C18_contigs_in_db.fa
###incluster###scp M_sp_C18_contigs_in_db.fa tdp56207@sapelo2.gacrc.uga.edu:/scratch/tdp56207/SK/p28/Marinobacter
###incluster###sbatch run
###incluster###Transfer to local the RAW-bam.tgz files
```

```
Export mode ................................: contigs
Output FASTA .................................: M_sp_C18_contigs_in_db.fa
```

Check and run `runcell19.sh`

```
#!ls *.tgz | awk '{print "tar xzvf " $0}'



tar xzvf OIL11-RAW.bam.tgz
tar xzvf OIL14-RAW.bam.tgz
tar xzvf OIL17-RAW.bam.tgz
tar xzvf OIL25-RAW.bam.tgz
tar xzvf OIL28-RAW.bam.tgz
tar xzvf OIL29-RAW.bam.tgz
tar xzvf OIL31-RAW.bam.tgz
tar xzvf OIL32-RAW.bam.tgz
tar xzvf OIL34-RAW.bam.tgz
tar xzvf OIL37-RAW.bam.tgz
tar xzvf OIL44-RAW.bam.tgz
tar xzvf OIL47-RAW.bam.tgz
tar xzvf OIL5-RAW.bam.tgz
tar xzvf OIL50-RAW.bam.tgz
tar xzvf OIL53-RAW.bam.tgz
tar xzvf OIL61-RAW.bam.tgz
tar xzvf OIL64-RAW.bam.tgz
tar xzvf OIL67-RAW.bam.tgz
tar xzvf OIL70-RAW.bam.tgz
tar xzvf OIL78-RAW.bam.tgz
```

```
tar xzvf OIL8-RAW.bam.tgz
tar xzvf OIL81-RAW.bam.tgz
tar xzvf OIL82-RAW.bam.tgz
tar xzvf OIL84-RAW.bam.tgz
tar xzvf OIL85-RAW.bam.tgz
tar xzvf OIL87-RAW.bam.tgz
tar xzvf OIL90-RAW.bam.tgz



#!ls *.bam | awk '{print "anvi-init-bam "$0" -o " $0 ".bam" }' |
sed -e 's/-RAW.bam.bam/.bam/'


anvi-init-bam OIL11-RAW.bam -o OIL11.bam
anvi-init-bam OIL14-RAW.bam -o OIL14.bam
anvi-init-bam OIL17-RAW.bam -o OIL17.bam
anvi-init-bam OIL25-RAW.bam -o OIL25.bam
anvi-init-bam OIL28-RAW.bam -o OIL28.bam
anvi-init-bam OIL29-RAW.bam -o OIL29.bam
anvi-init-bam OIL31-RAW.bam -o OIL31.bam
anvi-init-bam OIL32-RAW.bam -o OIL32.bam
anvi-init-bam OIL34-RAW.bam -o OIL34.bam
anvi-init-bam OIL37-RAW.bam -o OIL37.bam
anvi-init-bam OIL44-RAW.bam -o OIL44.bam
anvi-init-bam OIL47-RAW.bam -o OIL47.bam
anvi-init-bam OIL5-RAW.bam -o OIL5.bam
anvi-init-bam OIL50-RAW.bam -o OIL50.bam
anvi-init-bam OIL53-RAW.bam -o OIL53.bam
anvi-init-bam OIL61-RAW.bam -o OIL61.bam
anvi-init-bam OIL64-RAW.bam -o OIL64.bam
anvi-init-bam OIL67-RAW.bam -o OIL67.bam
anvi-init-bam OIL70-RAW.bam -o OIL70.bam
anvi-init-bam OIL78-RAW.bam -o OIL78.bam
anvi-init-bam OIL8-RAW.bam -o OIL8.bam
anvi-init-bam OIL81-RAW.bam -o OIL81.bam
anvi-init-bam OIL82-RAW.bam -o OIL82.bam
anvi-init-bam OIL84-RAW.bam -o OIL84.bam
anvi-init-bam OIL85-RAW.bam -o OIL85.bam
anvi-init-bam OIL87-RAW.bam -o OIL87.bam
anvi-init-bam OIL90-RAW.bam -o OIL90.bam



#!ls *.bam | awk '{print "anvi-init-bam "$0" -o " $0 ".bam" }' |
sed -e 's/-RAW.bam.bam/.bam/'

anvi-profile -i OIL11.bam --sample-name od_0 -c
02_CONTIGS/M_sp_C18-contigs.db
anvi-profile -i OIL14.bam --sample-name d_0  -c
02_CONTIGS/M_sp_C18-contigs.db
anvi-profile -i OIL17.bam --sample-name odn_0    -c
02_CONTIGS/M_sp_C18-contigs.db
```

```
anvi-profile -i OIL25.bam --sample-name bc_1    -c
02_CONTIGS/M_sp_C18-contigs.db
anvi-profile -i OIL28.bam --sample-name o_1_1    -c
02_CONTIGS/M_sp_C18-contigs.db
anvi-profile -i OIL29.bam --sample-name o_1_2    -c
02_CONTIGS/M_sp_C18-contigs.db
anvi-profile -i OIL31.bam --sample-name od_1_1    -c
02_CONTIGS/M_sp_C18-contigs.db
anvi-profile -i OIL32.bam --sample-name od_1_2 -c
02_CONTIGS/M_sp_C18-contigs.db
anvi-profile -i OIL34.bam --sample-name d_1    -c
02_CONTIGS/M_sp_C18-contigs.db
anvi-profile -i OIL37.bam --sample-name odn_1    -c
02_CONTIGS/M_sp_C18-contigs.db
anvi-profile -i OIL44.bam --sample-name bc_2    -c
02_CONTIGS/M_sp_C18-contigs.db
anvi-profile -i OIL47.bam --sample-name o_2    -c
02_CONTIGS/M_sp_C18-contigs.db
anvi-profile -i OIL50.bam --sample-name od_2    -c
02_CONTIGS/M_sp_C18-contigs.db
anvi-profile -i OIL53.bam --sample-name d_2 -c 02_CONTIGS/M_sp_C18-
contigs.db
anvi-profile -i OIL5.bam --sample-name bc_0    -c
02_CONTIGS/M_sp_C18-contigs.db
anvi-profile -i OIL61.bam --sample-name bc_3    -c
02_CONTIGS/M_sp_C18-contigs.db
anvi-profile -i OIL64.bam --sample-name o_3    -c
02_CONTIGS/M_sp_C18-contigs.db
anvi-profile -i OIL67.bam --sample-name od_3    -c
02_CONTIGS/M_sp_C18-contigs.db
anvi-profile -i OIL70.bam --sample-name d_3    -c
02_CONTIGS/M_sp_C18-contigs.db
anvi-profile -i OIL78.bam --sample-name bc_4    -c
02_CONTIGS/M_sp_C18-contigs.db
anvi-profile -i OIL81.bam --sample-name o_4_1 -c
02_CONTIGS/M_sp_C18-contigs.db
anvi-profile -i OIL82.bam --sample-name o_4_2    -c
02_CONTIGS/M_sp_C18-contigs.db
anvi-profile -i OIL84.bam --sample-name od_4_1    -c
02_CONTIGS/M_sp_C18-contigs.db
anvi-profile -i OIL85.bam --sample-name od_4_2    -c
02_CONTIGS/M_sp_C18-contigs.db
anvi-profile -i OIL87.bam --sample-name d_4    -c
02_CONTIGS/M_sp_C18-contigs.db
anvi-profile -i OIL8.bam --sample-name o_0    -c
02_CONTIGS/M_sp_C18-contigs.db
anvi-profile -i OIL90.bam --sample-name odn_4    -c
02_CONTIGS/M_sp_C18-contigs.db
```

In [2]:

```
#20
!anvi-merge OIL*ANVIO_PROFILE/PROFILE.db -c 02_CONTIGS/M_sp_C18-contigs.db
 -o SAMPLES-MERGED
```

```
[0m
```

```
WARNING
===============================================
Anvi'o just set the normalization values for each sample based on how many
mapped reads they contained. This information will only be used to calculate the
normalized coverage table. Here are those values: od_0: 0.22, d_0: 0.37, odn_0:
0.24, bc_1: 0.21, o_1_1: 0.12, o_1_2: 0.10, od_1_1: 0.13, od_1_2: 0.31, d_1:
0.31, odn_1: 0.31, bc_2: 0.29, o_2: 0.01, bc_0: 0.22, od_2: 0.27, d_2: 0.54,
bc_3: 0.56, o_3: 0.00, od_3: 0.85, d_3: 0.83, bc_4: 1.00, o_0: 0.21, o_4_1:
0.02, o_4_2: 0.00, od_4_1: 0.17, od_4_2: 0.90, d_4: 0.09, odn_4: 0.23

profiler_version ..............................: 35        ;5;0m..) ETA: \u221
e:\u221e:\u221e
output_dir ....................................: /Users/tito_miniconda/JOYE_LAB_A
NVIO_PROJECTS/SK_BACKUP/p28_pangenomes/Marinobacter/SAMPLES-MERGED
sample_id .....................................: SAMPLES_MERGED
description ...................................: None
profile_db ....................................: /Users/tito_miniconda/JOYE_LAB_A
NVIO_PROJECTS/SK_BACKUP/p28_pangenomes/Marinobacter/SAMPLES-MERGED/PROFILE.db
merged ........................................: True
contigs_db_hash ...............................: hashe147b970
num_runs_processed ............................: 27
merged_sample_ids .............................: bc_0, bc_1, bc_2, bc_3, bc_4,
                                                 d_0, d_1, d_2, d_3, d_4, o_0,
                                                 o_1_1, o_1_2, o_2, o_3, o_4_1,
                                                 o_4_2, od_0, od_1_1, od_1_2,
                                                 od_2, od_3, od_4_1, od_4_2,
                                                 odn_0, odn_1, odn_4
Common layer additional data keys .............: default
total_reads_mapped ............................: 8297, 8607, 6383, 3299, 1850,
                                                 5056, 6021, 3430, 2226, 21708,
                                                 8801, 15724, 19142, 304200,
                                                 479156, 113551, 626644, 8530,
                                                 14463, 5878, 6792, 2176, 10617,
                                                 2060, 7719, 5928, 8026
cmd_line ......................................: /Users/tito_miniconda/opt/minico
nda3/envs/anvio-7/bin/anvi-merge OIL11.bam-ANVIO_PROFILE/PROFILE.db OIL14.bam-AN
VIO_PROFILE/PROFILE.db OIL17.bam-ANVIO_PROFILE/PROFILE.db OIL25.bam-ANVIO_PROFIL
E/PROFILE.db OIL28.bam-ANVIO_PROFILE/PROFILE.db OIL29.bam-ANVIO_PROFILE/PROFILE.
db OIL31.bam-ANVIO_PROFILE/PROFILE.db OIL32.bam-ANVIO_PROFILE/PROFILE.db OIL34.b
am-ANVIO_PROFILE/PROFILE.db OIL37.bam-ANVIO_PROFILE/PROFILE.db OIL44.bam-ANVIO_P
ROFILE/PROFILE.db OIL47.bam-ANVIO_PROFILE/PROFILE.db OIL5.bam-ANVIO_PROFILE/PROF
ILE.db OIL50.bam-ANVIO_PROFILE/PROFILE.db OIL53.bam-ANVIO_PROFILE/PROFILE.db OIL
61.bam-ANVIO_PROFILE/PROFILE.db OIL64.bam-ANVIO_PROFILE/PROFILE.db OIL67.bam-ANV
IO_PROFILE/PROFILE.db OIL70.bam-ANVIO_PROFILE/PROFILE.db OIL78.bam-ANVIO_PROFIL
E/PROFILE.db OIL8.bam-ANVIO_PROFILE/PROFILE.db OIL81.bam-ANVIO_PROFILE/PROFILE.d
b OIL82.bam-ANVIO_PROFILE/PROFILE.db OIL84.bam-ANVIO_PROFILE/PROFILE.db OIL85.ba
m-ANVIO_PROFILE/PROFILE.db OIL87.bam-ANVIO_PROFILE/PROFILE.db OIL90.bam-ANVIO_PR
OFILE/PROFILE.db -c 02_CONTIGS/M_sp_C18-contigs.db -o SAMPLES-MERGED
clustering_performed ..........................: True
                                                                          ;5;0m
..) ETA: \u221e:\u221e:\u221e
WARNING
===============================================
Codon frequencies were not profiled, hence, these tables will be empty in the
merged profile database.


 [0m0m..) ETA: \u221e:\u221e:\u221e
* Anvi'o hierarchical clustering of contigs...

New items order ...............................: "tnf:euclidean:ward" (type
                                                 newick) has been added to the
                                                 database...
New items order ...............................: "tnf-cov:euclidean:ward" (type
                                                 newick) has been added to the
```

```
New items order ..............................: database...
                                                "cov:euclidean:ward" (type
                                                newick) has been added to the
                                                database...

* Additional data and layer orders...

Auxiliary Data ...............................: Found:        [0m
                                                /Users/tito_miniconda/JOYE_LAB_A
NVIO_PROJECTS/SK_BACKUP/p28_pangenomes/Marinobacter/SAMPLES-MERGED/AUXILIARY-DAT
A.db
                                                (v. 2)
Profile Super ................................: Initialized with all 238 splits:
                                                /Users/tito_miniconda/JOYE_LAB_A
NVIO_PROJECTS/SK_BACKUP/p28_pangenomes/Marinobacter/SAMPLES-MERGED/PROFILE.db
                                                (v. 35)
                                                                         [0m
Layer orders added
===============================================
* std_coverage
* mean_coverage
* mean_coverage_Q2Q3
* max_normalized_ratio
* relative_abundance
* detection
* abundance
* variability
                                                                         209m ET
A: Nonem
Data groups added
===============================================
* default (w/4 items)

* Happy \u2618
```

In [3]:

```
#21
!anvi-export-gene-coverage-and-detection -p SAMPLES-MERGED/PROFILE.db -c
 02_CONTIGS/M_sp_C18-contigs.db -O gene_cov_n_detection.txt
```

```
Auxiliary Data ...............................: Found:        [0m
                                                SAMPLES-MERGED/AUXILIARY-DATA.db
                                                (v. 2)
Profile Super ................................: Initialized with all 238 splits:
                                                SAMPLES-MERGED/PROFILE.db (v.
                                                35)
Gene coverages ...............................: gene_cov_n_detection.txt-GENE-CO
VERAGES.txt
Gene detection ...............................: gene_cov_n_detection.txt-GENE-DE
TECTION.txt
```

Here we need to go to display pangenome and we generate a single bin called "EVERYTHING" and then we summarize it

```
anvi-display-pan -p MARINOBACTER/Marinobacter-PAN.db -g
MARINOBACTER_GENOMES.db
`
```

In [4]:

```
#22
!anvi-summarize -p MARINOBACTER/Marinobacter-PAN.db -g MARINOBACTER_GENOMES.db
-C everything -o SUMMARY
#Decompress SUMMARY/Marinobacter_gene_clusters_summary.txt.gz
```

```
Genomes storage ...........................................: Initialized[48;5;
239m ETA: 0s
                                                              (storage hash:
                                                              hash220097ad)
Num genomes in storage ....................................: 180
Num genomes will be used ..................................: 180
Pan DB ....................................................: Initialized:
                                                              MARINOBACTER/Mari
nobacter-PAN.db
                                                              (v. 14)
Gene cluster homogeneity estimates ........................: Functional:
                                                              [YES]; Geometric:
                                                              [YES]; Combined:
                                                              [YES]

 [0m
* Gene clusters are initialized for all 44473 gene clusters in the database.


 [0m[38;5;0m ETA: 0s
Misc data reported for layers .............................: default

 [0m
Misc data reported for items ..............................: default

 [0m
HTML Output ...............................................: /Users/tito_minic
onda/JOYE_LAB_ANVIO_PROJECTS/SK_BACKUP/p28_pangenomes/Marinobacter/SUMMARY/inde
x.html
```

The following R script was used to incorporate gene coverage and gene detection profiles from the metatranscriptomes into the pangenome.

```
#!/usr/bin/env Rscript
rm(list=ls());
graphics.off();
setwd("/Users/tito_miniconda/JOYE_LAB_ANVIO_PROJECTS/SK_BACKUP/
p28_pangenomes/Marinobacter\")
#Libraries
library("dplyr")

#Input data

#Gene clusters from the pangenome
gene_clusters_df <- read.table(file =
'SUMMARY/Marinobacter_gene_clusters_summary.txt', header = TRUE,
sep = "\t", quote = "")

#Gene coverage profiles from the metatranscriptomic profiles
gene_coverages_df <- read.table(file='gene_cov_n_detection.txt-
GENE-COVERAGES.txt', header=TRUE, sep="\t", quote="")

#Gene detection profiles from the metatranscriptomic profiles
gene_detection_df <- read.table(file = 'gene_cov_n_detection.txt-
GENE-DETECTION.txt', header=TRUE, sep="\t", quote="")

#Let's remember that gene_clusters_df includes entries for all of
```

```
the genomes stored in the pangenome.
#At some point we need to subset those gene_callers_id entries that
belong to the reference genome of the
#transcriptomic merged profiles.

ref_genome =  'M_sp_C18'

#Creating zero dataframes to store processed data



gene_cluster_names <- gene_clusters_df[,colnames(gene_clusters_df)
%in% c('gene_cluster_id')]
gene_cluster_names <- unique(gene_cluster_names)
samples_names <- colnames(gene_coverages_df)
samples_names <- samples_names[2:length(samples_names)]
num_cols = length(samples_names)
num_rows = length(gene_cluster_names)
out_coverage_df = data.frame(matrix(0,ncol = num_cols, nrow =
num_rows))
colnames(out_coverage_df ) <- samples_names
rownames(out_coverage_df ) <- gene_cluster_names
out_detection_df <- out_coverage_df

#Loop to calculate the maximum coverage and maximum detection
observed on each gene cluster.
for (gene_cluster in unique(gene_clusters_df$gene_cluster_id)){
  df <- gene_clusters_df[gene_clusters_df$gene_cluster_id ==
gene_cluster, ]
  df2 <- df[df$genome_name == ref_genome,]
  if(length(df2$gene_callers_id) > 0){
    for(my_gene_callers_id in df2$gene_callers_id){
      #For coverages
      df3 <- gene_coverages_df[gene_coverages_df$key ==
my_gene_callers_id, ]
      x1 <- as.vector(df3)
      x2 <- x1[2:length(x1)]
      #For detections
      df4 <- gene_detection_df[gene_detection_df$key ==
my_gene_callers_id, ]
      x3 <- as.vector(df4)
      x4 <- x3[2:length(x3)]

      for(i in 1:length(x2)){
        #For coverages
        the_max = max(x2[i],out_coverage_df[gene_cluster,i])
        out_coverage_df[gene_cluster,i] <- the_max
        #For detections
        the_max2 = max(x4[i],out_detection_df[gene_cluster,i])
        out_detection_df[gene_cluster,i] <- the_max2
      }

    }
```

```
    }

  }

  #Adding prefixes and merging dataframes into one
  colnames(out_coverage_df) <-
  paste('cov',colnames(out_coverage_df),sep='_')
  colnames(out_detection_df) <-
  paste('det',colnames(out_detection_df),sep='_')

  out_coverage_df <- tibble::rownames_to_column(out_coverage_df,
  "gene_cluster_id")
  out_detection_df <- tibble::rownames_to_column(out_detection_df,
  "gene_cluster_id")

  output_df <- merge(out_coverage_df,
  out_detection_df,by="gene_cluster_id")

  #Export data frame into an anvio friendly table
  write.table(output_df, "gene_clusters_additional_data.txt",
  quote=FALSE, sep="\t", na="", row.names=FALSE)
```

In [6]:

```
#23
#Next line removes the columns of coverage. Detection columns are enough to be uploaded to anvio.
!cat gene_clusters_additional_data.txt | awk '{print $1, "\t", $29, "\t", $30, "\t", $31, "\t", $32, "\t", $33,
 "\t", $34, "\t",$35, "\t", $36, "\t", $37, "\t", $38, "\t", $39, "\t", $40, "\t", $41, "\t", $42, "\t", $43,
 "\t", $44, "\t", $45, "\t", $46, "\t", $47, "\t", $48, "\t", $49, "\t", $50, "\t", $51, "\t", $52, "\t", $53,
 "\t", $54, "\t", $55}' | tr -d \" \" > gene_clusters_additional_data_v2.txt\n"
```

Now let's import our table into the Pangenomic anvio database

In [7]:

```
#24
!anvi-import-misc-data gene_clusters_additional_data_v2.txt -p
 MARINOBACTER/Marinobacter-PAN.db --target-data-table items --just-do-it
```

```
[0m
* gene_clusters_additional_data_v2.txt successfully loaded


New data for 'items' in data group 'default'
===============================================
Data key "det_bc_0" ..........................: Predicted type: float
Data key "det_bc_1" ..........................: Predicted type: float
Data key "det_bc_2" ..........................: Predicted type: float
Data key "det_bc_3" ..........................: Predicted type: float
Data key "det_bc_4" ..........................: Predicted type: float
Data key "det_d_0" ...........................: Predicted type: float
Data key "det_d_1" ...........................: Predicted type: float
Data key "det_d_2" ...........................: Predicted type: float
Data key "det_d_3" ...........................: Predicted type: float
Data key "det_d_4" ...........................: Predicted type: float
Data key "det_o_0" ...........................: Predicted type: float
Data key "det_o_1_1" .........................: Predicted type: float
Data key "det_o_1_2" .........................: Predicted type: float
Data key "det_o_2" ...........................: Predicted type: float
Data key "det_o_3" ...........................: Predicted type: float
Data key "det_o_4_1" .........................: Predicted type: float
Data key "det_o_4_2" .........................: Predicted type: float
```

```
Data key "det_od_0" ..........................: Predicted type: float
Data key "det_od_1_1" ........................: Predicted type: float
Data key "det_od_1_2" ........................: Predicted type: float
Data key "det_od_2" ..........................: Predicted type: float
Data key "det_od_3" ..........................: Predicted type: float
Data key "det_od_4_1" ........................: Predicted type: float
Data key "det_od_4_2" ........................: Predicted type: float
Data key "det_odn_0" .........................: Predicted type: float
Data key "det_odn_1" .........................: Predicted type: float
Data key "det_odn_4" .........................: Predicted type: float

                                                                    209m ET
A: None
NEW DATA
===============================================
Database .....................................: pan
Data group ...................................: default
Data table ...................................: items
New data keys ................................: det_bc_0, det_bc_1, det_bc_2,
                                                det_bc_3, det_bc_4, det_d_0,
                                                det_d_1, det_d_2, det_d_3,
                                                det_d_4, det_o_0, det_o_1_1,
                                                det_o_1_2, det_o_2, det_o_3,
                                                det_o_4_1, det_o_4_2, det_od_0,
                                                det_od_1_1, det_od_1_2,
                                                det_od_2, det_od_3, det_od_4_1,
                                                det_od_4_2, det_odn_0,
                                                det_odn_1, det_odn_4.


\u2713 anvi-import-misc-data took 0:00:03.154345
```

In [11]:

```
#25
#Import state

!anvi-import-state -p MARINOBACTER/Marinobacter-PAN.db -s pan_state.json -n v1
```

```
Done .........................................: State "v1" is added to the
                                                database
```

Filtering pangenome by mapping recovery

First we want to collapse or split those gene clusters that did not recover transcriptomic reads on *any* library. To do this:

1. Create a bin called `no_signals` and select everything. Tip: Choose a yellow color for this.
2. Create a bin called `T_signals`. Tip: Choose a black color for this.
3. Having the can `T_signals` selected, use the expression `Det X` > 0, where `X` is any of the treatments, and then click on append to selected bin. You will have to loop manually through each treatments.
4. Save the bin collection as `bins_by_recovery`.

This would look like this before splitting.

In [14]:

```
Image(filename='Figure_1.jpg')
```

Out[14]:

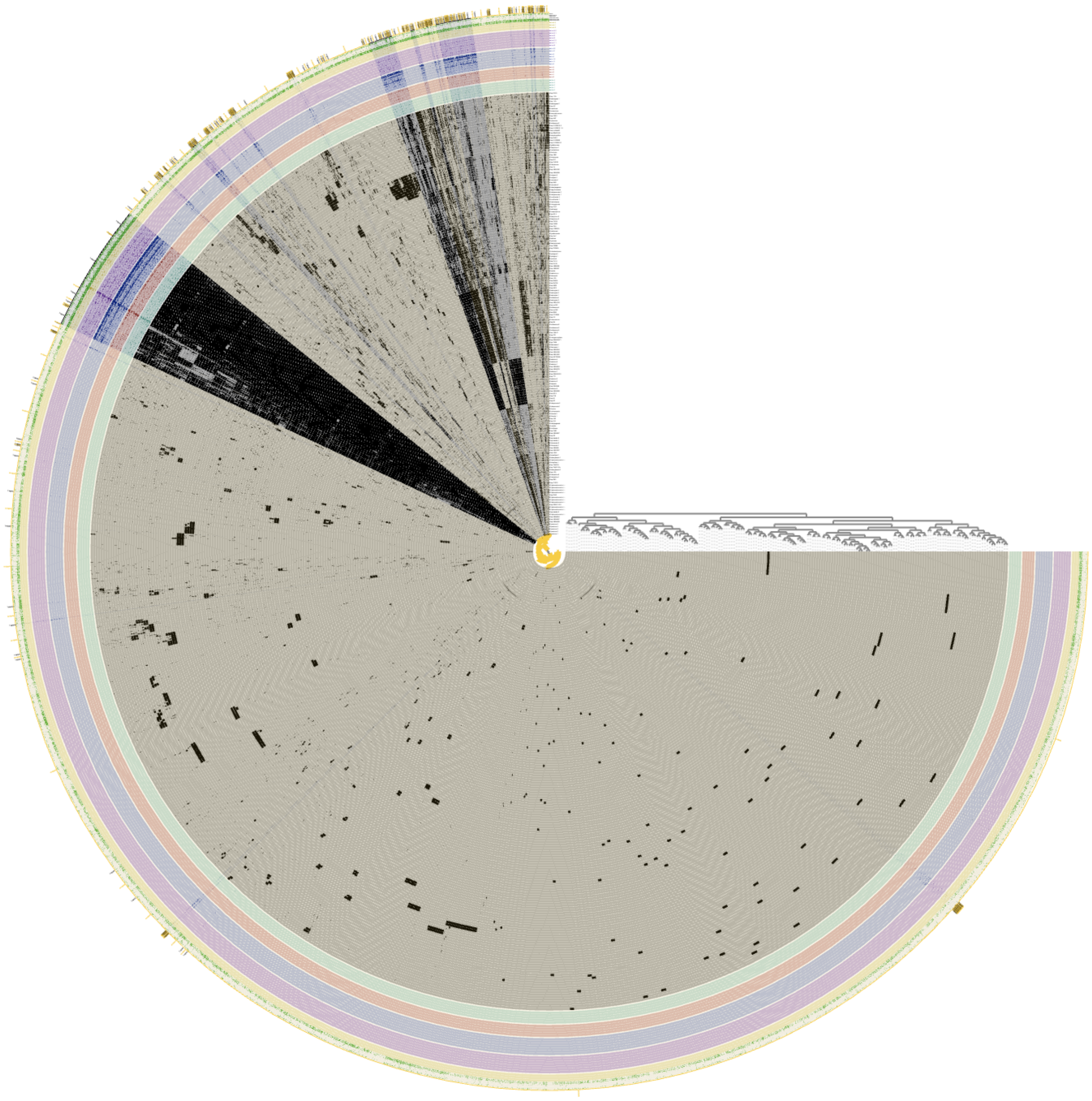

We are going to proceed to split by recovery:

In [15]:

```
#26
!anvi-split -p MARINOBACTER/Marinobacter-PAN.db -g MARINOBACTER_GENOMES.db
 -C bins_by_recovery -o SPLIT_PANs
```

```
Genomes storage ..............................................: Initialized[48;5;
239m ETA: 0s
                                                                (storage hash:
                                                                hash220097ad)
Num genomes in storage .......................................: 180
Num genomes will be used .....................................: 180
Pan DB .......................................................: Initialized:
                                                                MARINOBACTER/Mari
nobacter-PAN.db
                                                                (v. 14)
Gene cluster homogeneity estimates ...........................: Functional:
                                                                [YES]; Geometric:
                                                                [YES]; Combined:
                                                                [YES]
```

```
 [0m
* Gene clusters are initialized for all 44473 gene clusters in the database.

                                                                          [0m
WARNING
==============================================================
Anvi'o is about to start splitting your bins into individual, self-contained
anvi'o profiles. This is quite a tricky operation, and even if it finishes
successfully, you must double check everyting in the resulting profiles to make
sure things worked as expected. Although we are doing our best to test all
these, variation between projects make it impossible to be 100% sure.

Collections ................................: The collection 'DEFAULT' that
                                              describes 3910 splits in a
                                              single bin was successfully
                                              added to the to the database at
                                              'SPLIT_PANs/T_signals/PAN.db'.
                                              Here is a full list of the bin
                                              names in this collection:
                                              ALL_SPLITS.
New items order ............................: "frequency:euclidean:ward" (type
                                              newick) has been added to the
                                              database...
                                                                          [0m
WARNING
===============================================
Clustering for "frequency:euclidean:ward" is already in the database. It will be
replaced with the new content.

New items order ............................: "frequency:euclidean:ward" (type
                                              newick) has been added to the
                                              database...
New items order ............................: "presence-absence:euclidean:war
d"

                                              (type newick) has been added to
                                              the database...

WARNING
==============================================================
It seems you have more than 20,000 splits in this particular bin. This is the
soft limit for anvi'o to attempt to create a hierarchical clustering of your
splits (which becomes the center tree in all anvi'o displays). If you want a
hierarchical clustering to be done anyway, you can re-run the splitting process
only for this bin by adding these parameters to your run: '--bin-id no_signals
--enforce-hierarchical-clustering'. If you feel like you are lost, don't
hesitate to get in touch with anvi'o developers.

Collections ................................: The collection 'DEFAULT' that
                                              describes 40563 splits in a
                                              single bin was successfully
                                              added to the to the database at
                                              'SPLIT_PANs/no_signals/PAN.db'.
                                              Here is a full list of the bin
                                              names in this collection:
                                              ALL_SPLITS.
Num bins processed ..............................................: 2
Output directory ................................................: /Users/tito_minic
onda/JOYE_LAB_ANVIO_PROJECTS/SK_BACKUP/p28_pangenomes/Marinobacter/SPLIT_PANs
```

From now on we are interested on the fraction of the pangenome that recovered transcriptomic signals.

```
anvi-display-pan -p SPLIT_PANs/T_signals/PAN.db -g
MARINOBACTER_GENOMES.db
```

Differentially Expressed Genes Layers

Counting mapped reads per gene using htseq

This section I am following https://metagenomics-workshop.readthedocs.io/en/latest/annotation/quantification.html. First we need to extract the a GFF file from the annotation of anvio.

In [16]:

```
#27
!anvi-get-sequences-for-gene-calls -c 02_CONTIGS/M_sp_C18-contigs.db -o
 ref_genome.gff --export-gff3
```

```
Contigs DB ...................................: Initialized:  [0m
                                                02_CONTIGS/M_sp_C18-contigs.db
                                                (v. 20)

WARNING
===============================================
You did not provide any gene caller ids. As a result, anvi'o will give you back
sequences for every 4638 gene call stored in the contigs database.

                                                                          [0m
THE MORE YOU KNOW \U0001f308
===============================================
Someone asked the Contigs Superclass to initialize only a subset of contig
sequences. Usually this is a good thing and means that some good code somewhere
is looking after you. Just FYI, this class will only know about 42 contig
sequences instead of all the things in the database.

Output .......................................: ref_genome.gff
```

In [17]:

```
#28
#Convert gff to gtf format
!cat ref_genome.gff | grep -v "#" | grep "ID=" | cut -f1 -d ';' | sed 's/ID=//g' | cut -f1,4,5,7,9 | awk -v
 OFS='\t' '{print $1, "PRODIGAL","CDS",$2,$3,".",$4,".","gene_id " $5}' >  ref_genome.gtf
```

Run

```
bash runcellA.sh
```

Next run in separate consoles the test .py files.

```
python test11.py

...

python test90.py
```

In [154…

```
#29
#Move results to sam2counts folder
!mkdir sam2counts
!mv OIL*_mapcounts.csv sam2counts

!cut -f4,5,9 ref_genome.gtf | sed 's/gene_id //g' | gawk '{print $3,$2-$1+1}' |
 | tr ' ' '\t' > ref_genome.genelengths
```

```
read_files = ['OIL11_f.fa','OIL25_f.fa','OIL31_f.fa','OIL37_f.fa','OIL50_f.fa','OIL61_f.fa','OIL70_f.fa','OIL82_f.fa','OIL87_f.fa',
'OIL11_r.fa','OIL25_r.fa','OIL31_r.fa','OIL37_r.fa','OIL50_r.fa','OIL61_r.fa','OIL70_r.fa','OIL82_r.fa','OIL87_r.fa','OIL14_f.fa','OIL28_f.fa',
'OIL32_f.fa','OIL44_f.fa','OIL53_f.fa','OIL64_f.fa','OIL78_f.fa','OIL84_f.fa','OIL8_f.fa','OIL14_r.fa','OIL28_r.fa','OIL32_r.fa','OIL44_r.fa',
'OIL53_r.fa','OIL64_r.fa','OIL78_r.fa','OIL84_r.fa','OIL8_r.fa','OIL17_f.fa','OIL29_f.fa','OIL34_f.fa','OIL47_f.fa','OIL5_f.fa','OIL67_f.fa',
'OIL81_f.fa','OIL85_f.fa','OIL90_f.fa','OIL17_r.fa','OIL29_r.fa','OIL34_r.fa','OIL47_r.fa','OIL5_r.fa','OIL67_r.fa','OIL81_r.fa','OIL85_r.fa',
'OIL90_r.fa']
sample_ids  = ['od_0','bc_1','od_1_1','odn_1',  'od_2','bc_3','d_3',  'o_4_2',  'd_4','od_0','bc_1','od_1_1','odn_1',  'od_2','bc_3','d_3',  'o_4_2',
'd_4',
 'd_0', 'o_1_1', 'od_1_2','bc_2','d_2', 'o_3', 'bc_4','od_4_1','o_0','d_0', 'o_1_1', 'od_1_2','bc_2','d_2', 'o_3', 'bc_4','od_4_1','o_0','odn_0',
 'o_1_2', 'd_1', 'o_2', 'bc_0', 'od_3','o_4_1', 'od_4_2','odn_4','odn_0', 'o_1_2', 'd_1', 'o_2', 'bc_0', 'od_3','o_4_1', 'od_4_2','odn_4']
```

The following steps will calculate TPM values for contigs or genes based on count files

TPM values are defined as in Wagner et al (Theory in Biosciences) 2012.

$$TPM_i = \frac{rg*rl*10^6}{f*T}$$

rg: reads mapped to gene g

rl: read length

f: feature length

$T = \sum_i \frac{rg*rl}{f}$ for all i genes

For this calculation we need to know the average read length

```
reads_vec = ['5319504','2599528','8665216','4856793','6533258','2742491','4059579','2650493','7410098','5319504','2599528','8665216','4856793',
'6533258','2742491','4059579','2650493','7410098','3008480','7395105','6898923','3008495','4951454','4587833','3455976','8377386','4570606',
'3008480','7395105','6898923','3008495','4951454','4587833','3455976','8377386','4570606','6753873','6867899','4699125','2359617','4238895',
'5235701','6756836','2180731','9152171','6753873','6867899','4699125','2359617','4238895','5235701','6756836','2180731','9152171']
total_bp_in_lib_vec = ['529556914','258358315','860860737','471734413','637162234','260148861','385237010','247448659','720970318','527979712',
'257355106','861328463','480966006','648081155','270020595','396129359','256912761','728241939','299481143','733713272','684447286','299191768',
'483932122','434358626','324037321','788966972','455633481','298087834','733573535','687327923','298745950','490543446','451526124','335033361',
'814356799','453712387','667248186','684652368','460414958','232691190','422225840','497983867','640662788','203200060','899578610','665617351',
'681864181','466017852','233762389','420716951','511511621','658014105','211690251','902781824']

read_length_d = {'library': read_files, 'sample_id' : sample_ids, 'num_reads_in_lib' : reads_vec, 'total_bp_in_lib': total_bp_in_lib_vec }
read_length_df = pd.DataFrame(read_length_d)
```

In [156…

```
read_length_df
```

Out[156…

	library	sample_id	num_reads_in_lib	total_bp_in_lib
0	OIL11_f.fa	od_0	5319504	529556914
1	OIL25_f.fa	bc_1	2599528	258358315
2	OIL31_f.fa	od_1_1	8665216	860860737
3	OIL37_f.fa	odn_1	4856793	471734413
4	OIL50_f.fa	od_2	6533258	637162234
5	OIL61_f.fa	bc_3	2742491	260148861
6	OIL70_f.fa	d_3	4059579	385237010
7	OIL82_f.fa	o_4_2	2650493	247448659
8	OIL87_f.fa	d_4	7410098	720970318
9	OIL11_r.fa	od_0	5319504	527979712
10	OIL25_r.fa	bc_1	2599528	257355106
11	OIL31_r.fa	od_1_1	8665216	861328463
12	OIL37_r.fa	odn_1	4856793	480966006
13	OIL50_r.fa	od_2	6533258	648081155
14	OIL61_r.fa	bc_3	2742491	270020595

	library	sample_id	num_reads_in_lib	total_bp_in_lib
15	OIL70_r.fa	d_3	4059579	396129359
16	OIL82_r.fa	o_4_2	2650493	256912761
17	OIL87_r.fa	d_4	7410098	728241939
18	OIL14_f.fa	d_0	3008480	299481143
19	OIL28_f.fa	o_1_1	7395105	733713272
20	OIL32_f.fa	od_1_2	6898923	684447286
21	OIL44_f.fa	bc_2	3008495	299191768
22	OIL53_f.fa	d_2	4951454	483932122
23	OIL64_f.fa	o_3	4587833	434358626
24	OIL78_f.fa	bc_4	3455976	324037321
25	OIL84_f.fa	od_4_1	8377386	788966972
26	OIL8_f.fa	o_0	4570606	455633481
27	OIL14_r.fa	d_0	3008480	298087834
28	OIL28_r.fa	o_1_1	7395105	733573535
29	OIL32_r.fa	od_1_2	6898923	687327923
30	OIL44_r.fa	bc_2	3008495	298745950
31	OIL53_r.fa	d_2	4951454	490543446
32	OIL64_r.fa	o_3	4587833	451526124
33	OIL78_r.fa	bc_4	3455976	335033361
34	OIL84_r.fa	od_4_1	8377386	814356799
35	OIL8_r.fa	o_0	4570606	453712387
36	OIL17_f.fa	odn_0	6753873	667248186
37	OIL29_f.fa	o_1_2	6867899	684652368
38	OIL34_f.fa	d_1	4699125	460414958
39	OIL47_f.fa	o_2	2359617	232691190
40	OIL5_f.fa	bc_0	4238895	422225840
41	OIL67_f.fa	od_3	5235701	497983867
42	OIL81_f.fa	o_4_1	6756836	640662788
43	OIL85_f.fa	od_4_2	2180731	203200060
44	OIL90_f.fa	odn_4	9152171	899578610
45	OIL17_r.fa	odn_0	6753873	665617351
46	OIL29_r.fa	o_1_2	6867899	681864181
47	OIL34_r.fa	d_1	4699125	466017852
48	OIL47_r.fa	o_2	2359617	233762389
49	OIL5_r.fa	bc_0	4238895	420716951

	library	sample_id	num_reads_in_lib	total_bp_in_lib
50	OIL67_r.fa	od_3	5235701	511511621
51	OIL81_r.fa	o_4_1	6756836	658014105
52	OIL85_r.fa	od_4_2	2180731	211690251
53	OIL90_r.fa	odn_4	9152171	902781824

In [157…

```
#32
read_length_df_v2 = read_length_df[['sample_id','num_reads_in_lib', 'total_bp_in_lib']]
read_length_df_v2[['num_reads_in_lib', 'total_bp_in_lib']] = read_length_df_v2[['num_reads_in_lib', 'total_bp_in_lib']].astype('int64')
read_length_df_v2 = read_length_df_v2.groupby(['sample_id']).sum()
read_length_df_v2['avg_read_length'] = read_length_df_v2['total_bp_in_lib']/read_length_df_v2['num_reads_in_lib']
read_length_df_v2
```

```
/Users/tito_miniconda/opt/miniconda3/envs/anvio-7/lib/python3.6/site-packages/pa
ndas/core/frame.py:3494: SettingWithCopyWarning:
A value is trying to be set on a copy of a slice from a DataFrame.
Try using .loc[row_indexer,col_indexer] = value instead

See the caveats in the documentation: http://pandas.pydata.org/pandas-docs/stabl
e/user_guide/indexing.html#returning-a-view-versus-a-copy
  self[k1] = value[k2]
```

Out[157…

	num_reads_in_lib	total_bp_in_lib	avg_read_length
**sample_id**			
bc_0	8477790	842942791	99.429544
bc_1	5199056	515713421	99.193665
bc_2	6016990	597937718	99.374890
bc_3	5484982	530169456	96.658377
bc_4	6911952	659070682	95.352323
d_0	6016960	597568977	99.314102
d_1	9398250	926432810	98.575034
d_2	9902908	974475568	98.402971
d_3	8119158	781366369	96.237365
d_4	14820196	1449212257	97.786308
o_0	9141212	909345868	99.477604
o_1_1	14790210	1467286807	99.206624
o_1_2	13735798	1366516549	99.485778
o_2	4719234	466453579	98.840952
o_3	9175666	885884750	96.547188
o_4_1	13513672	1298676893	96.100963
o_4_2	5300986	504361420	95.144832
od_0	10639008	1057536626	99.401808

	num_reads_in_lib	total_bp_in_lib	avg_read_length
sample_id			
od_1_1	17330432	1722189200	99.373703
od_1_2	13797846	1371775209	99.419519
od_2	13066516	1285243389	98.361598
od_3	10471402	1009495488	96.404998
od_4_1	16754772	1603323771	95.693559
od_4_2	4361462	414890311	95.126430
odn_0	13507746	1332865537	98.674163
odn_1	9713586	952700419	98.079167
odn_4	18304342	1802360434	98.466278

In [ ]:

```
#32B
r'/Users/tito_miniconda/JOYE_LAB_ANVIO_PROJECTS/SK_BACKUP/p28_pangenomes/Marinobacter/sam2counts/OIL11_mapcounts.csv'
```

In [162…

```
#33
sample_names = pd.Series(read_length_df_v2.index.values)
my_lengths = r'/Users/tito_miniconda/JOYE_LAB_ANVIO_PROJECTS/SK_BACKUP/p28_pange
gene_lengths = pd.read_table(my_lengths, header=None, index_col=0, names=['gene_id','gene_length'])
root = r'/Users/tito_miniconda/JOYE_LAB_ANVIO_PROJECTS/SK_BACKUP/p28_pangenomes/Marinobacter/sam2counts/'
my_tpm_df = pd.DataFrame()
for i in np.arange(sample_names.size):
    counts_file = sample_names[i] + '_mapcounts.txt'
    location = root + counts_file
    rg = pd.read_table(location, header=None, index_col=0, names=['gene_id', 'count'])
    rg_v2 = rg.drop(rg.tail(5).index)
    ## Intersect with genes in the gene length file
    rg_v3 = rg_v2.iloc[list(set(gene_lengths.index).intersection(set(rg_v2.index.astype('int64'))))]
    gene_lengths_v2 = gene_lengths.iloc[list(rg_v3.index)]
    rg_v3.index = rg_v3.index.astype('int64')
    gene_lengths_v2.index = gene_lengths_v2.index.astype('int64')
    ## Average read length for sample
    rl = read_length_df_v2.at[sample_names[i],'avg_read_length']
    ## Calculate T for sample
    T = np.sum(rl * rg_v3['count'].divide(gene_lengths_v2['gene_length']))
    ## Calculate TPM for sample
    tpm = (1e6*rl/T)*rg_v3['count'].divide(gene_lengths_v2['gene_length'])
    ## Create dataframe
    tpm_se = pd.DataFrame(tpm, columns=[sample_names[i]])
    ## Concatenate to results
    my_tpm_df = pd.concat([my_tpm_df,tpm_se],axis=1)
```

In [163…

```
#This is to check we are not getting a matrix full of zeros
my_tpm_df[my_tpm_df.sum(axis=1) > 0].head()
```

Out[163…

	bc_0	bc_1	bc_2	bc_3	bc_4	d_0	d_1	d_2	d_3	d_4	...	od_0	od_1_1	od_1_2	o[illegible]

gene_id	bc_0	bc_1	bc_2	bc_3	bc_4	d_0	d_1	d_2	d_3	d_4	...	od_0	od_1_1	od_1_2	o[illegible]
**gene_id**															
0	0.0	0.0	0.0	0.0	0.0	0.0	0.0	0.0	0.0	0.0	...	0.0	0.0	0.0	
1	0.0	0.0	0.0	0.0	0.0	0.0	0.0	0.0	0.0	0.0	...	0.0	0.0	0.0	
2	0.0	0.0	0.0	0.0	0.0	0.0	0.0	0.0	0.0	0.0	...	0.0	0.0	0.0	
3	0.0	0.0	0.0	0.0	0.0	0.0	0.0	0.0	0.0	0.0	...	0.0	0.0	0.0	
4	0.0	0.0	0.0	0.0	0.0	0.0	0.0	0.0	0.0	0.0	...	0.0	0.0	0.0	

5 rows × 27 columns

In [164…

```
#34
my_tpm_df.to_csv('tpm.csv', sep='\t')
```

DESeq in R

Please close this notebook and open the notebook `DE_Analysis.ipynb` to run the differential expression analysis.

Load DE profiles and convert from gene to gene_cluster table

In [7]:

```
#35
!sqlite3 -header -csv SPLIT_PANs/T_signals/PAN.db "select * from gene_cluster_frequencies;" > pan_split_gene_clusters.csv
!mv pan_split_gene_clusters.csv SPLIT_PANs/
pan_split_geneclusters_df = pd.read_csv(r'/Users/tito_miniconda/JOYE_LAB_ANVIO_PROJECTS/SK_BACKUP/p28_pangenomes/Marinobacter/SPLIT_PANs/pan_split_gene_clusters.csv')
!DESEQ_df = pd.read_csv(r'/Users/tito_miniconda/JOYE_LAB_ANVIO_PROJECTS/SK_BACKUP/p28_pangenomes/Marinobacter/Marinobacter_DE_df.txt', sep='\t')
!sig_df = DESEQ_df[DESEQ_df.padj < 0.05]
sig_df.rename(columns={'Gene':'gene_caller_id'}, inplace=True)


#Building output scheme
DE_for_anvio_df = pan_split_geneclusters_df.iloc[:,0:9]
DE_for_anvio_df.columns= ['gene_cluster_id', 'A', 'B', 'C', 'D', 'E', 'F', 'G',
DE_for_anvio_df.A = 0
DE_for_anvio_df.B = 0
DE_for_anvio_df.C = 0
DE_for_anvio_df.D = 0
DE_for_anvio_df.E = 0
DE_for_anvio_df.F = 0
DE_for_anvio_df.G = 0
DE_for_anvio_df.H = 0
DE_for_anvio_df.columns= ['gene_cluster_id', 'DE_d!A', 'DE_d!B', 'DE_o!A', 'DE_o!B', 'DE_od!A', 'DE_od!B', 'DE_odn!A', 'DE_odn!B',]
DE_for_anvio_df = DE_for_anvio_df.drop_duplicates()
```

```
/Users/tito_miniconda/opt/miniconda3/envs/anvio-7/lib/python3.6/site-packages/pa
ndas/core/frame.py:4223: SettingWithCopyWarning:
A value is trying to be set on a copy of a slice from a DataFrame
```

```
See the caveats in the documentation: http://pandas.pydata.org/pandas-docs/stabl
e/user_guide/indexing.html#returning-a-view-versus-a-copy
  return super().rename(**kwargs)
```

In [8]:

```
sig_df
```

Out[8]:

	gene_caller_id	baseMean	log2FoldChange	lfcSE	stat	pvalue	p
141	1295	26663.209963	-15.935259	4.450030	-3.580933	3.423694e-04	1.68705
142	1296	24375.410822	-15.711408	4.450032	-3.530628	4.145739e-04	1.99831
147	1301	23768.078325	-15.765563	4.450031	-3.542798	3.959055e-04	1.91354
155	1309	23768.078325	-15.765563	4.450031	-3.542798	3.959055e-04	1.91354
166	1320	26992.153937	-15.951716	4.450030	-3.584631	3.375547e-04	1.67264
...	...	...	...	...	...	...	
10454	4580	20092.421643	30.000000	3.720604	8.063207	7.431873e-16	6.58327
10458	4585	38406.626162	30.000000	2.881701	10.410519	2.220073e-25	5.83857
10459	4587	32362.607941	30.000000	3.090652	9.706690	2.823583e-22	4.39393
10461	4589	5736.375222	30.000000	4.491412	6.679414	2.398996e-11	2.71249
10779	833	24813.668119	30.000000	4.503417	6.661609	2.708459e-11	2.71249

1352 rows × 9 columns

In [9]:

```
DE_for_anvio_df
```

Out[9]:

	gene_cluster_id	DE_d!A	DE_d!B	DE_o!A	DE_o!B	DE_od!A	DE_od!B	DE_odn!A	DE_odn!
0	GC_00001020	0	0	0	0	0	0	0	
1	GC_00002235	0	0	0	0	0	0	0	
2	GC_00003652	0	0	0	0	0	0	0	
3	GC_00002180	0	0	0	0	0	0	0	
4	GC_00000793	0	0	0	0	0	0	0	
...	...	...	...	...	...	...	...	...	.
3905	GC_00004053	0	0	0	0	0	0	0	
3906	GC_00001176	0	0	0	0	0	0	0	
3907	GC_00006024	0	0	0	0	0	0	0	

	gene_cluster_id	DE_d!A	DE_d!B	DE_o!A	DE_o!B	DE_od!A	DE_od!B	DE_odn!A	DE_odn!
3908	GC_00001166	0	0	0	0	0	0	0	
3909	GC_00002689	0	0	0	0	0	0	0	

3910 rows × 9 columns

In [10]:

```
#36
#Merging annotation data with DE data
!sqlite3 -header -csv SPLIT_PANs/T_signals/PAN.db "select * from gene_clusters;" > pan_split_gene_clusters_v2.csv
!mv pan_split_gene_clusters_v2.csv SPLIT_PANs/
pan_split_geneclusters_df_v2 = pd.read_csv(r'/Users/tito_miniconda/JOYE_LAB_ANVIO_PROJECTS/SK_BACKUP/p28_pangenomes/Marinobacter/SPLIT_PANs/pan_split_gene_clusters_v2.csv')
pan_split_geneclusters_df_v2 = pan_split_geneclusters_df_v2[pan_split_geneclusters_df_v2.genome_name == 'M_sp_C18']
```

In [11]:

```
#This number should be bigger than next cell
len(pan_split_geneclusters_df_v2.gene_caller_id.unique())
```

Out[11]: 4087

In [12]:

```
len(sig_df.gene_caller_id.unique())
```

Out[12]: 968

In [13]:

```
#37
temp_df = pd.merge(pan_split_geneclusters_df_v2,sig_df,on='gene_caller_id',how='left')

#Print description
temp_df.to_csv(r'/Users/tito_miniconda/JOYE_LAB_ANVIO_PROJECTS/SK_BACKUP/p28_pangenomes/Marinobacter/Marinobacter_DE_w_description.txt', index = False, sep='\t')
```

In [14]:

```
#38
for index,row in DE_for_anvio_df.iterrows():
    #print(row[0])
    #my_genecluster_id = 'GC_00001516'
    my_genecluster_id = row[0]
    temp2_df = temp_df[temp_df.gene_cluster_id == my_genecluster_id]
    if not temp2_df.empty:
        up_df = temp2_df[temp2_df.log2FoldChange > 0]
        if not up_df.empty:
            for index2,row2 in up_df.iterrows():
                if row2[10] == 'd':
                    DE_for_anvio_df.at[index,'DE_d!B'] = DE_for_anvio_df.loc[index,'DE_d!B'] + 1
                if row2[10] == 'o':
                    DE_for_anvio_df.at[index,'DE_o!B'] = DE_for_anvio_df.loc[index,'DE_o!B'] + 1
                if row2[10] == 'od':
                    DE_for_anvio_df.at[index,'DE_od!B'] = DE_for_anvio_df.loc[index,'DE_od!B'] + 1
                if row2[10] == 'odn':
                    DE_for_anvio_df.at[index,'DE_odn!B'] = DE_for_anvio_df.loc[index,'DE_odn!B'] + 1
        down_df = temp2_df[temp2_df.log2FoldChange < 0]
        if not down_df.empty:
            for index2,row2 in down_df.iterrows():
                if row2[10] == 'd':
                    DE_for_anvio_df.at[index,'DE_d!A'] = DE_for_anvio_df.loc[index,'DE_d!A'] + 1
```

```
                if row2[10] == 'o':
                    DE_for_anvio_df.at[index,'DE_o!A'] = DE_for_anvio_df.loc[index,'DE_o!A'] + 1
                if row2[10] == 'od':
                    DE_for_anvio_df.at[index,'DE_od!A'] = DE_for_anvio_df.loc[index,'DE_od!A'] + 1
                if row2[10] == 'odn':
                    DE_for_anvio_df.at[index,'DE_odn!A'] = DE_for_anvio_df.loc[index,'DE_odn!A'] + 1
```

In [15]:

```
DE_for_anvio_df
```

Out[15]:

	gene_cluster_id	DE_d!A	DE_d!B	DE_o!A	DE_o!B	DE_od!A	DE_od!B	DE_odn!A	DE_odn!
0	GC_00001020	0	0	0	0	0	0	0	
1	GC_00002235	0	0	0	0	0	0	0	
2	GC_00003652	0	0	0	0	0	0	0	
3	GC_00002180	0	0	0	0	0	0	0	
4	GC_00000793	0	0	0	0	0	0	0	
...	...	...	...	...	...	...	...	...	.
3905	GC_00004053	0	0	1	0	0	0	0	
3906	GC_00001176	0	0	0	0	0	0	0	
3907	GC_00006024	0	0	0	0	0	0	0	
3908	GC_00001166	0	0	0	0	0	0	0	
3909	GC_00002689	0	0	0	0	0	0	0	

3910 rows × 9 columns

In [16]:

```
DE_for_anvio_df[DE_for_anvio_df.sum(axis=1) > 0]
```

Out[16]:

	gene_cluster_id	DE_d!A	DE_d!B	DE_o!A	DE_o!B	DE_od!A	DE_od!B	DE_odn!A	DE_odn!
5	GC_00000940	0	0	1	0	0	0	0	
6	GC_00000499	0	0	1	0	0	0	0	
11	GC_00002217	0	0	1	0	0	0	0	
23	GC_00008050	1	0	0	0	1	0	0	
26	GC_00003033	0	0	0	0	0	0	0	
...	...	...	...	...	...	...	...	...	.
3888	GC_00002351	0	0	1	0	0	0	0	
3891	GC_00001925	0	0	1	0	0	0	0	
3897	GC_00008612	0	0	0	0	0	0	0	
3901	GC_00000775	1	0	0	0	0	0	0	
3905	GC_00004053	0	0	1	0	0	0	0	

826 rows × 9 columns

In [17]:
```
#It is necessary to check for empty columns. Empty columns will generate errors
DE_for_anvio_df.sum()
#   DE_odn!A has 0 DE genes.
```

Out[17]:
```
gene_cluster_id    GC_00001020GC_00002235GC_00003652GC_00002180GC...
DE_d!A                                                            92
DE_d!B                                                           177
DE_o!A                                                           345
DE_o!B                                                           141
DE_od!A                                                          167
DE_od!B                                                          133
DE_odn!A                                                           2
DE_odn!B                                                         155
dtype: object
```

In [18]:
```
#DE table able to be loaded into anvio
DE_for_anvio_df.to_csv(r'/Users/tito_miniconda/JOYE_LAB_ANVIO_PROJECTS/SK_BACKUP/p28_pangenomes/Marinobacter/Marinobacter_DE_for_anvio.txt', index = False, sep='\t')
```

In [19]:
```
#39
!anvi-import-misc-data Marinobacter_DE_for_anvio.txt -p SPLIT_PANs/T_signals/PAN.db --target-data-table items --just-do-it
```

```
[0m
* Marinobacter_DE_for_anvio.txt successfully loaded


New data for 'items' in data group 'default'
===============================================
Data key "DE_d!A" ............................: Predicted type: stackedbar
Data key "DE_d!B" ............................: Predicted type: stackedbar
Data key "DE_o!A" ............................: Predicted type: stackedbar
Data key "DE_o!B" ............................: Predicted type: stackedbar
Data key "DE_od!A" ...........................: Predicted type: stackedbar
Data key "DE_od!B" ...........................: Predicted type: stackedbar
Data key "DE_odn!A" ..........................: Predicted type: stackedbar
Data key "DE_odn!B" ..........................: Predicted type: stackedbar


WARNING
===============================================
The following keys in your data dict will replace the ones that are already in
your pan database items table and default data group: DE_d!A, DE_d!B, DE_o!A,
DE_o!B, DE_od!A, DE_od!B, DE_odn!A, DE_odn!B.


WARNING
===============================================
Data from the table 'items' for the following data keys in data group 'default'
removed from the database: 'DE_d!A, DE_d!B, DE_o!A, DE_o!B, DE_od!A, DE_od!B,
DE_odn!A, DE_odn!B'. #SAD.

                                                                      209m ET
A: None
NEW DATA
===============================================
Database .....................................: pan
Data group ...................................: default
Data table ...................................: items
New data keys ................................: DE_d!A, DE_d!B, DE_o!A, DE_o!B,
                                                DE_od!A, DE_od!B, DE_odn!A,
```

DE_odn!B.

In [179…

```
#40
!anvi-import-state -p SPLIT_PANs/T_signals/PAN.db -s pan_state.json -n v1
```

```
WARNING
===============================================
Previous entries for "v1" is being removed from "states"

Done .......................................: State "v1" is added to the
                                              database
```

This is a good time to check with

```
anvi-display-pan -p SPLIT_PANs/T_signals/PAN.db -g
MARINOBACTER_GENOMES.db
```

If everything looks right, it is adviced to go ahead and select the core by:

- Create a new bin called "Core"
- Go to search and choose "Search gene clusters using filters"
- Set "min number of genomes gene cluster occurs" to 78
- Append selection to bin

In [26]:

```
Image(filename='Figure_2.png')
```

Out[26]:

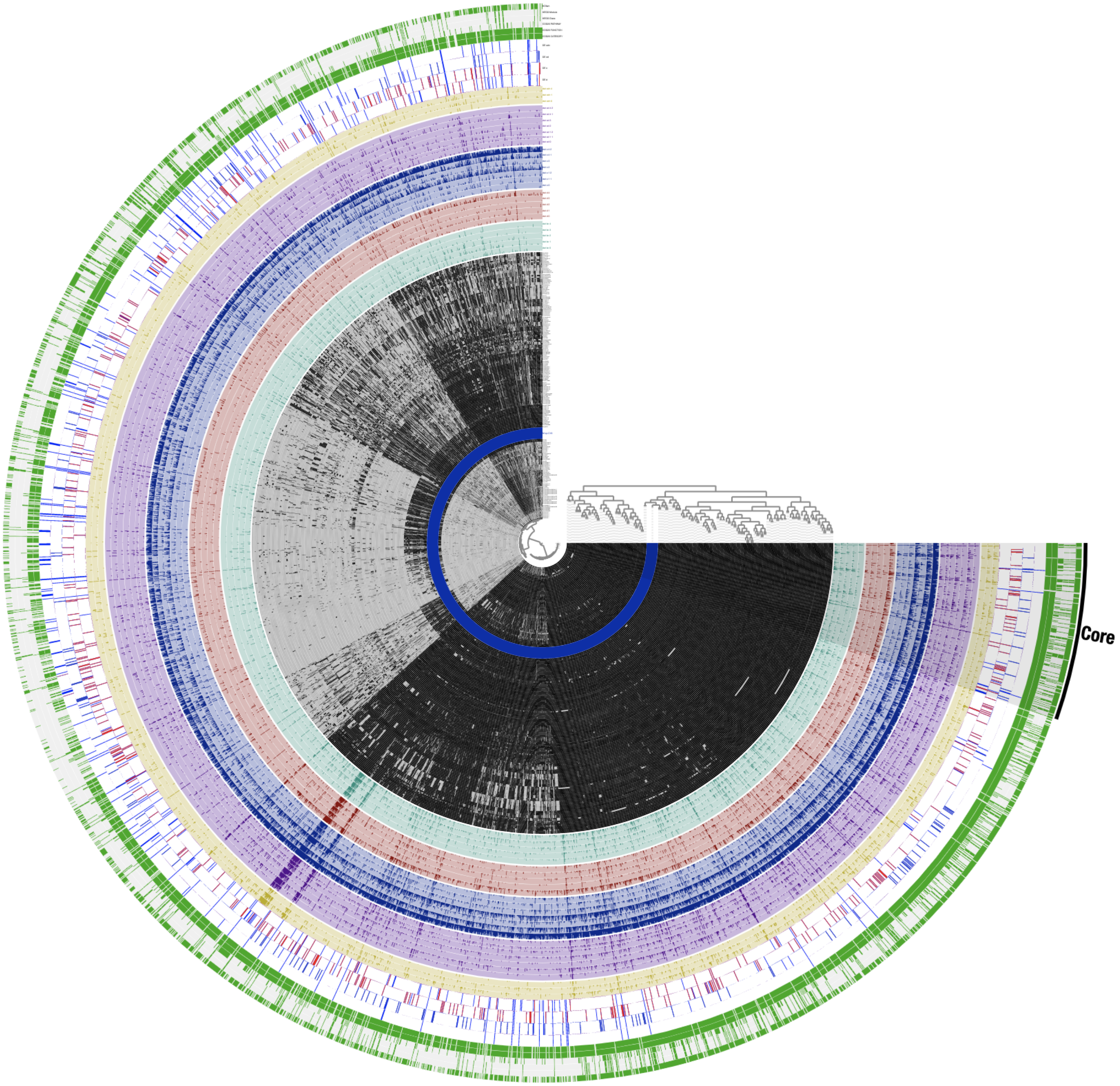

Barplot of up down DE genes

The following barplot will be added to the anvio plot. Anvio can add a layer to samples but not to the DE layer. So we do it here and later we add in inkscape.

In [20]:

```
#41
DE_for_anvio_df = pd.read_csv(r'/Users/tito_miniconda/JOYE_LAB_ANVIO_PROJECTS/SK_BACKUP/p28_pangenomes/Marinobacter/Marinobacter_DE_for_anvio.txt', sep = '\t')
col_vec = DE_for_anvio_df.columns
col_vec = col_vec[1:9]
d = col_vec.str.split('!').tolist()
barplot_data_df = pd.DataFrame(d)
barplot_data_df = barplot_data_df.rename(columns={0: "Treatment", 1: "DE_type"})
n = barplot_data_df.shape[0]
d = np.zeros(n)
barplot_data_df['DE_total'] = d.tolist()
for index,row in DE_for_anvio_df.iterrows():

    barplot_data_df['DE_total'] = row[1:9].tolist() + barplot_data_df.DE_total
```

In [21]:

```
barplot_data_df
```

Out[21]:

	Treatment	DE_type	DE_total
0	DE_d	A	92.0
1	DE_d	B	177.0
2	DE_o	A	345.0
3	DE_o	B	141.0
4	DE_od	A	167.0
5	DE_od	B	133.0
6	DE_odn	A	2.0
7	DE_odn	B	155.0

In [22]:

```
#42
barplot_data_df = barplot_data_df.replace(to_replace=r'^A$', value='B_DownReg', regex=True)
barplot_data_df = barplot_data_df.replace(to_replace=r'^B$', value='A_UpReg', regex=True)
barplot_4_anvio = alt.Chart(barplot_data_df).mark_bar().encode(

    x='Treatment',
    y='DE_total',
    color=alt.Color('DE_type', scale=alt.Scale(domain=['A_UpReg', 'B_DownReg'], range=['blue', 'red']))

).properties(
    width=100,
    height=100
)
```

In [23]:

```
#43
barplot_4_anvio.save('barplot_4_anvio.svg')
```

DE genes functional (Supplementary) annotation table

In [27]:

```
#44
!anvi-export-functions -c 02_CONTIGS/M_sp_C18-contigs.db -o M_sp_C18-contigs_annotations.txt
```

```
Annotation sources ............................: KOfam, COG20_PATHWAY,
                                                 KEGG_Module, Transfer_RNAs,
                                                 COG20_FUNCTION, KEGG_Class,
                                                 COG20_CATEGORY.
Number of functions reported ..................: 12,083    [0m
Output file ...................................: M_sp_C18-contigs_annotations.txt
```

In [2]:

```
#45
ANNOT_df = pd.read_csv(r'/Users/tito_miniconda/JOYE_LAB_ANVIO_PROJECTS/SK_BACKUP/p28_pangenomes/Marinobacter/M_sp_C18-contigs_annotations.txt', sep = '\t')
DESEQ_df = pd.read_csv(r'/Users/tito_miniconda/JOYE_LAB_ANVIO_PROJECTS/SK_BACKUP/p28_pangenomes/Marinobacter/Marinobacter_DE_df.txt', sep='\t')
sig_df = DESEQ_df[DESEQ_df.padj < 0.05]
sig_df.rename(columns={'Gene':'gene_callers_id'}, inplace=True)
temp_df = pd.merge(sig_df,ANNOT_df,on='gene_callers_id',how='left')
temp_df.to_csv('DIFFEX_ANNOT.csv')
```

```
#From DIFFEX_ANNOT.csv we are generating a supplementary table for the paper.
temp_df["new_id"] =  temp_df["gene_callers_id"].astype(str) + temp_df["treatment"]
```

```
/Users/tito_miniconda/opt/miniconda3/envs/anvio-7/lib/python3.6/site-packages/pa
ndas/core/frame.py:4223: SettingWithCopyWarning:
A value is trying to be set on a copy of a slice from a DataFrame

See the caveats in the documentation: http://pandas.pydata.org/pandas-docs/stabl
e/user_guide/indexing.html#returning-a-view-versus-a-copy
  return super().rename(**kwargs)
```

In [3]:

```
#46

## INICIALIZACION ##

ids = np.unique(temp_df.new_id.values)

i = ids[0]

test = temp_df[temp_df.new_id == i]

test2 = test.loc[:, "gene_callers_id":"abs_diff_mean"]

test2 = test2.drop_duplicates()

test3 = copy(test2)

test3 = test3.applymap(lambda x: None)

test3.columns = ["COG20_CATEGORY", "COG20_CATEGORY_description", "COG20_CATEGORY_e_value",
                 "COG20_FUNCTION", "COG20_FUNCTION_description", "COG20_FUNCTION_e_value",
                 "COG20_PATHWAY", "COG20_PATHWAY_description", "COG20_PATHWAY_e_value"]
test4 = copy(test3)

test4.columns = ["KOfam", "KOfam_description", "KOfam_e_value",


                 "KEGG_Module", "KEGG_Module_description", "KEGG_Module_e_value"
                 "KEGG_Class", "KEGG_Class_description", "KEGG_Class_e_value"]

test5 = pd.concat([test2, test4, test3], axis=1)

diffex_annot_df = test5.drop(test5.index[range(1)])


## LOOP ##

for i in ids:
    test = temp_df[temp_df.new_id == i]
    test2 = test.loc[:, "gene_callers_id":"abs_diff_mean"]
    test2 = test2.drop_duplicates()
    test3 = copy(test2)

    test3 = test3.applymap(lambda x: None)

    test3.columns = ["COG20_CATEGORY", "COG20_CATEGORY_description", "COG20_CATEGORY_e_value",
                     "COG20_FUNCTION", "COG20_FUNCTION_description", "COG20_FUNCTION_e_value",
                     "COG20_PATHWAY", "COG20_PATHWAY_description", "COG20_PATHWAY_e_value"]
    test4 = copy(test3)
```

```
test4.columns = ["KOfam", "KOfam_description", "KOfam_e_value",
                 "KEGG_Module", "KEGG_Module_description", "KEGG_Module_e_value",
                 "KEGG_Class", "KEGG_Class_description", "KEGG_Class_e_value"]


test5 = pd.concat([test2, test4, test3], axis=1)
if test5.shape[0] > 1:
    print(test5)

try:
    test5.iloc[0,9] = test[test.source == 'KOfam'].accession.values[0]
except IndexError:
    test5.iloc[0,9] = None

try:
    test5.iloc[0,10] = test[test.source == 'KOfam'].function.values[0]
except IndexError:
    test5.iloc[0,10] = None

try:
    test5.iloc[0,11] = test[test.source == 'KOfam'].e_value.values[0]
except IndexError:
    test5.iloc[0,11] = None

try:
    test5.iloc[0,12] = test[test.source == 'KEGG_Module'].accession.values[0]
except IndexError:
    test5.iloc[0,12] = None

try:
    test5.iloc[0,13] = test[test.source == 'KEGG_Module'].function.values[0]
except IndexError:
    test5.iloc[0,13] = None

try:
    test5.iloc[0,14] = test[test.source == 'KEGG_Module'].e_value.values[0]
except IndexError:
    test5.iloc[0,14] = None

try:
    test5.iloc[0,15] = test[test.source == 'KEGG_Class'].accession.values[0]
except IndexError:
    test5.iloc[0,15] = None

try:
    test5.iloc[0,16] = test[test.source == 'KEGG_Class'].function.values[0]
except IndexError:
    test5.iloc[0,16] = None

try:
    test5.iloc[0,17] = test[test.source == 'KEGG_Class'].e_value.values[0]
except IndexError:
    test5.iloc[0,17] = None

try:
    test5.iloc[0,18] = test[test.source == 'COG20_CATEGORY'].accession.values[0]
except IndexError:
    test5.iloc[0,18] = None

try:
```

```
        test5.iloc[0,19] = test[test.source == 'COG20_CATEGORY'].function.values[0]
except IndexError:
        test5.iloc[0,19] = None

    try:
        test5.iloc[0,20] = test[test.source == 'COG20_CATEGORY'].e_value.values[0]
    except IndexError:
        test5.iloc[0,20] = None

    try:
        test5.iloc[0,21] = test[test.source == 'COG20_FUNCTION'].accession.values[0]
    except IndexError:
        test5.iloc[0,21] = None

    try:
        test5.iloc[0,22] = test[test.source == 'COG20_FUNCTION'].function.values[0]
    except IndexError:
        test5.iloc[0,22] = None

    try:
        test5.iloc[0,23] = test[test.source == 'COG20_FUNCTION'].e_value.values[0]
    except IndexError:
        test5.iloc[0,23] = None

    try:
        test5.iloc[0,24] = test[test.source == 'COG20_PATHWAY'].accession.values[0]
    except IndexError:
        test5.iloc[0,24] = None

    try:
        test5.iloc[0,25] = test[test.source == 'COG20_PATHWAY'].function.values[0]
    except IndexError:
        test5.iloc[0,25] = None

    try:
        test5.iloc[0,26] = test[test.source == 'COG20_PATHWAY'].e_value.values[0]
    except IndexError:
        test5.iloc[0,26] = None


    frames = [diffex_annot_df, test5]

    diffex_annot_df = pd.concat(frames)

diffex_annot_df.to_csv('DIFFEX_ANNOT_v2.csv')
```

Selecting and splitting only GC containing DE genes.

Anvio diagram is getting too difficult to label names. So we are going to select and split bins that only contain DE genes. Selection was done using the search box and now we proceed to split again.

In [32]:

```
#47
!anvi-split -p SPLIT_PANs/T_signals/PAN.db -g MARINOBACTER_GENOMES.db -C DE_Genes -o SPLIT_DE_Genes
```

```
Genomes storage ....................................: Initialized[48;5;
```

```
239m ETA: 0s
                                                               (storage hash:
                                                               hash220097ad)
Num genomes in storage ......................................: 180
Num genomes will be used ....................................: 180
Pan DB ......................................................: Initialized:
                                                               SPLIT_PANs/T_sign
als/PAN.db
                                                               (v. 14)
Gene cluster homogeneity estimates ..........................: Functional:
                                                               [YES]; Geometric:
                                                               [YES]; Combined:
                                                               [YES]

 [0m
* Gene clusters are initialized for all 3910 gene clusters in the database.

                                                                         [0m
WARNING
==============================================================
Anvi'o is about to start splitting your bins into individual, self-contained
anvi'o profiles. This is quite a tricky operation, and even if it finishes
successfully, you must double check everyting in the resulting profiles to make
sure things worked as expected. Although we are doing our best to test all
these, variation between projects make it impossible to be 100% sure.

Collections ...................................: The collection 'DEFAULT' that
                                                 describes 826 splits in a single
                                                 bin was successfully added to
                                                 the to the database at
                                                 'SPLIT_DE_Genes/DE_Genes/PAN.d
b'.
                                                 Here is a full list of the bin
                                                 names in this collection:
                                                 ALL_SPLITS.
New items order ...............................: "frequency:euclidean:ward" (type
                                                 newick) has been added to the
                                                 database...
                                                                         [0m
WARNING
===============================================
Clustering for "frequency:euclidean:ward" is already in the database. It will be
replaced with the new content.

New items order ...............................: "frequency:euclidean:ward" (type
                                                 newick) has been added to the
                                                 database...
New items order ...............................: "presence-absence:euclidean:war
d"
                                                 (type newick) has been added to
                                                 the database...
Num bins processed ..........................................: 1
Output directory ............................................: /Users/tito_minic
onda/JOYE_LAB_ANVIO_PROJECTS/SK_BACKUP/p28_pangenomes/Marinobacter/SPLIT_DE_Gene
s
```

anvi-display-pan -p SPLIT_DE_Genes/DE_genes/PAN.db -g MARINOBACTER_GENOMES.db
`

In [33]:

```
Image(filename='Figure_3.png')
```

Out[33]:

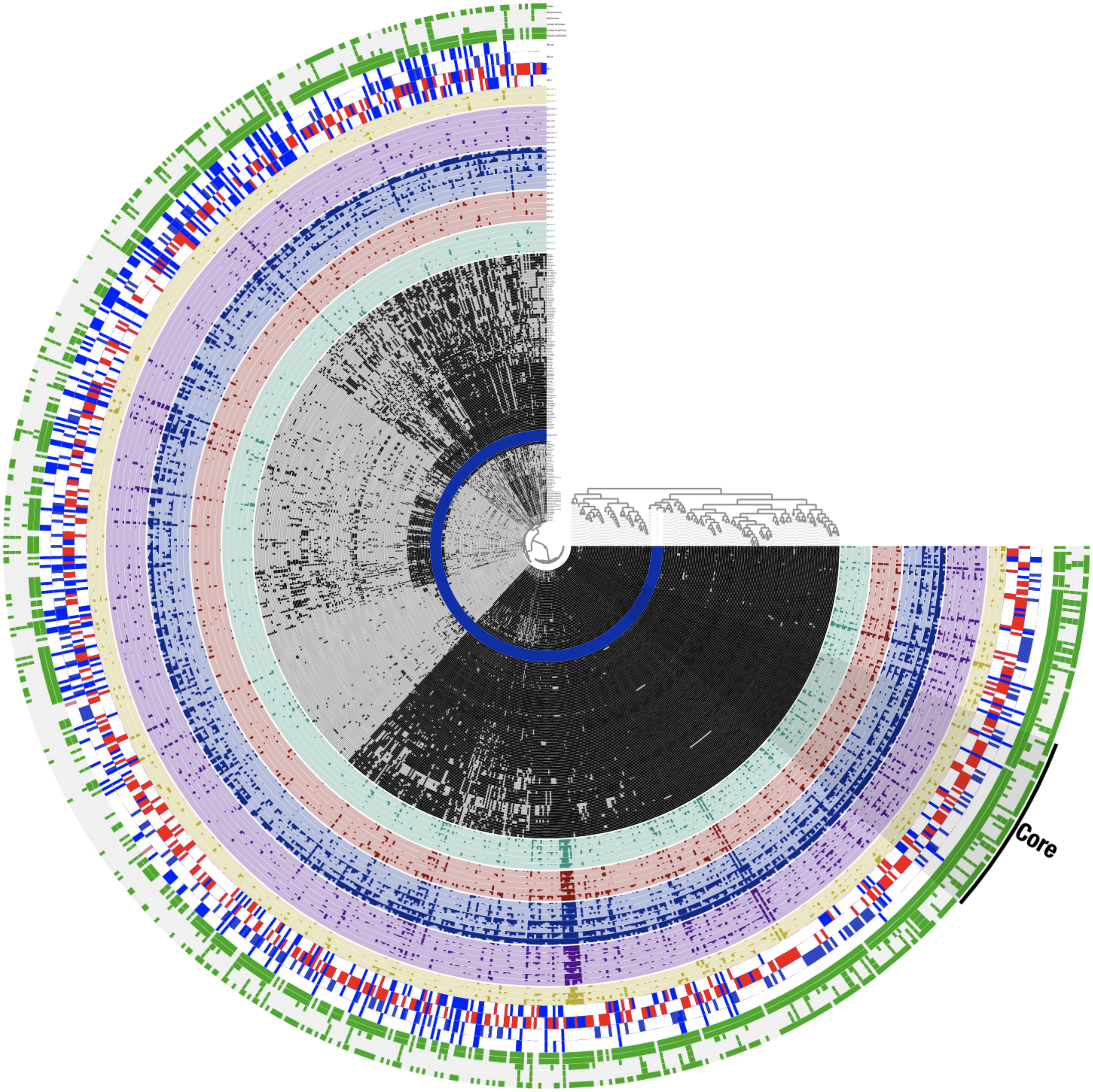

Static HTML Summary

Now our CA-Metatranscriptome is ready to generate an static HTML output.

anvi-summarize -p SPLIT_PANs/T_signals/PAN.db -g MARINOBACTER_GENOMES.db -o SPLIT_T_signals-SUMMARY -C DEFAULT

In [ ]:

```
!anvi-summarize -p SPLIT_DE_Genes/DE_genes/PAN.db -g MARINOBACTER_GENOMES.db -o SPLIT_DE_signals-SUMMARY -C core_acc
```
